# Synthesis and nucleophilic aromatic substitution of 3-fluoro-5-nitro-1-(pentafluorosulfanyl)benzene

**DOI:** 10.3762/bjoc.12.21

**Published:** 2016-02-03

**Authors:** Javier Ajenjo, Martin Greenhall, Camillo Zarantonello, Petr Beier

**Affiliations:** 1 Institute of Organic Chemistry and Biochemistry, v.v.i., Academy of Sciences of the Czech Republic, Flemingovo nám. 2, 166 10 Prague 6, Czech Republic; 2 F2 Chemicals Ltd, Lea Lane, Lea Town, Preston, PR4 0RZ, UK

**Keywords:** direct fluorination, fluorine, nucleophilic aromatic substitution, pentafluorosulfanyl group, vicarious nucleophilic substitution

## Abstract

3-Fluoro-5-nitro-1-(pentafluorosulfanyl)benzene was prepared by three different ways: as a byproduct of direct fluorination of 1,2-bis(3-nitrophenyl)disulfane, by direct fluorination of 4-nitro-1-(pentafluorosulfanyl)benzene, and by fluorodenitration of 3,5-dinitro-1-(pentafluorosulfanyl)benzene. The title compound was subjected to a nucleophilic aromatic substitution of the fluorine atom with oxygen, sulfur and nitrogen nucleophiles affording novel (pentafluorosulfanyl)benzenes with 3,5-disubstitution pattern. Vicarious nucleophilic substitution of the title compound with carbon, oxygen, and nitrogen nucleophiles provided 3-fluoro-5-nitro-1-(pentafluorosulfanyl)benzenes substituted in position four.

## Introduction

Organic compounds with a pentafluorosulfanyl (SF_5_) group are promising candidates in the development of new agrochemicals, pharmaceuticals and advanced materials [1–3]. This is due to an unusual combination of properties such as high stability [4], lipophilicity [5] and strong electron-withdrawing character [6] similar but more extreme than the trifluoromethyl group. Currently, the limiting factor for a more widespread use of SF_5_ compounds is the low accessibility of basic building blocks and the lack of understanding their chemical behavior. For aromatic SF_5_ compounds, two main synthetic approaches exist. The first one is a direct fluorination of nitro-substituted diaryl disulfides leading to 3- or 4-nitro-1-(pentafluorosulfanyl)benzenes [7–10]. This reaction is conducted in tens of kilogram scale in industry. The second method is known as Umemoto’s synthesis and starts with aromatic thiols or diaryl disulfides which are converted to arylsulfur chlorotetrafluorides [11–12]. Fluorination in the second step affords arylsulfur pentafluorides [11,13]. Umemoto’s synthesis does not require handling the elemental fluorine, gives better yields, and displays a wider substrate scope than the direct fluorination method. However, nitro(pentafluorosulfanyl)benzenes are widely-available primary industrial products and starting materials to other SF_5_-benzenes which were prepared by reduction followed by condensation or diazotization chemistry [8,14–16], S_E_Ar [17], S_N_Ar [18–24], or metal-catalyzed cross-coupling reactions [25–27]. Synthetic methods to novel SF_5_-containing building blocks are sought after and drive the development of applications of these compounds. In this work, we explore S_N_Ar chemistry of 3-fluoro-5-nitro-1-(pentafluorosulfanyl)benzene which was initially obtained as a minor byproduct of direct fluorination of 1,2-bis(3-nitrophenyl)disulfane but can be also prepared by other methods, and show that substitution for aromatic fluorine or hydrogen atoms provide a range of novel structurally diverse SF_5_-benzenes.

## Results and Discussion

**Scheme 1:**
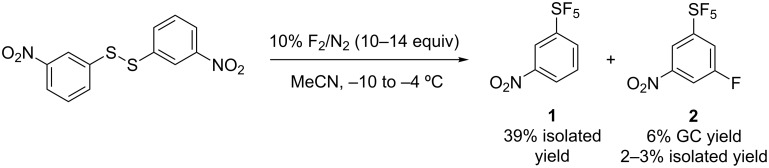
Direct fluorination of 1,2-bis(3-nitrophenyl)disulfane.

**Scheme 2:**
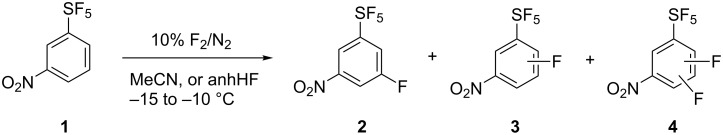
Direct fluorination of 3-nitro-1-(pentafluorosulfanyl)benzene (**1**).

**Figure 1:**
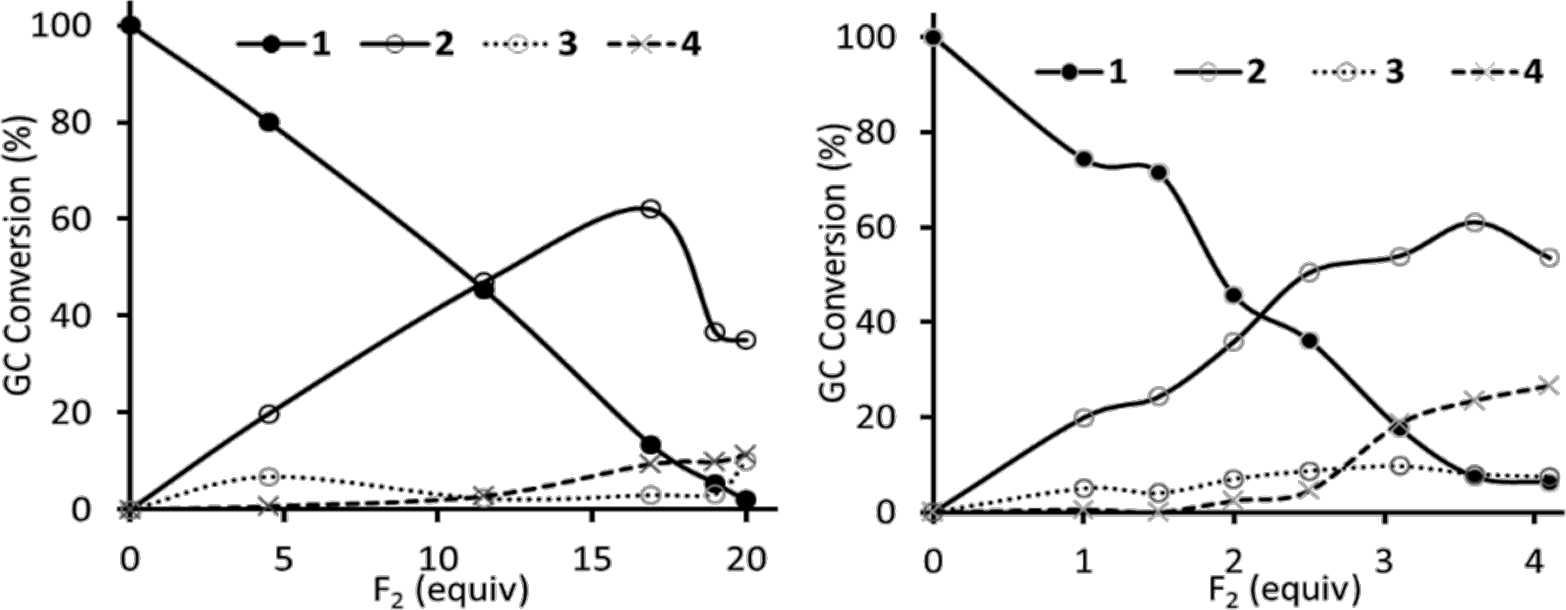
Conversion vs added fluorine equivalents for the fluorination of **1** in MeCN (left) and anhydrous HF (right).

**Scheme 3:**
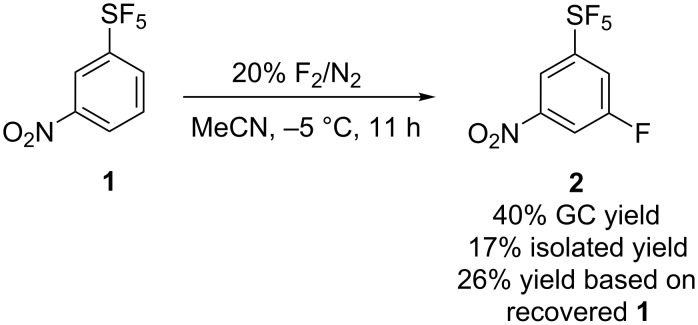
Preparative fluorination of 3-nitro-1-(pentafluorosulfanyl)benzene (**1**).

Direct fluorination of 1,2-bis(3-nitrophenyl)disulfane on kilogram scale led to the main product 3-nitro-1-(pentafluorosulfanyl)benzene (**1**) which was isolated in 39% yield [8] and a byproduct 3-fluoro-5-nitro-1-(pentafluorosulfanyl)benzene (**2**) in 6% GC yield (2–3% isolated yield, Scheme 1). Compound **2** was isolated from the mixture by distillation; redistillation afforded **2** in 99% purity (detected by GC). However, for investigations of S_N_Ar of **2**, a more efficient process for its synthesis was required. With higher excess of fluorine, the **2**:**1** ratio increased. Therefore, the synthesis of **2** by the direct fluorination of **1** was investigated (Scheme 2 and Figure 1). The reaction progress was monitored by GC analysis. In acetonitrile, the reaction required more than 16 equivalents of F_2_ to reach maximal conversion of **2** (around 60%). With higher amounts of fluorine gas the conversion of **2** sharply decreased and the amounts of (poly)fluorinated aromatic byproducts **3** and **4** increased (Figure 1, left). The amount of non-volatile material (tar) in the final product mixture (after 20 equiv of F_2_ added) was 28% by weight as determined by Kugelrohr distillation. When the direct fluorination was performed in anhydrous HF a markedly different result was obtained. To reach maximal conversion of **2** (around 60%), only a 3.6-fold excess of F_2_ was required (Figure 1, right). However, in this case, higher amounts of byproducts, particularly difluoroderivatives **4** were observed. The amount of tar after the addition of 4.2 equivalents of F_2_ was only 5%. Purification of compound **2** from **3** and **4** by distillation or column chromatography was not successful. Therefore, for preparative experiments we decided to run the fluorination in MeCN to about 40% conversion and isolated **2** from unreacted **1** by flash chromatography (Scheme 3).

**Scheme 4:**
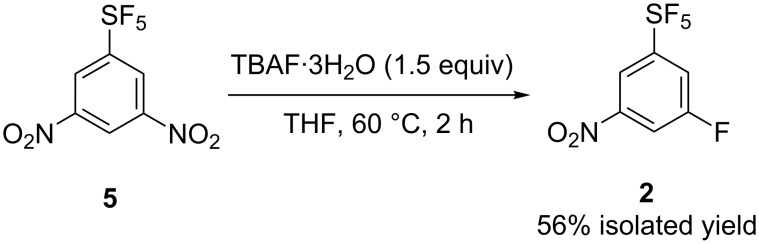
Synthesis of **2** by fluorodenitration of **5**.

Another method for the synthesis of **2** is the fluorodenitration of known 3,5-dinitro-1-(pentafluorosulfanyl)benzene (**5**) [10,28]. The reaction with TBAF hydrate resulted in clean substitution of only one nitro group for the fluorine atom (Scheme 4).

Nucleophilic aromatic substitution of fluorine leading to compounds **3** was investigated (Table 1). With low-boiling alcohols (MeOH, EtOH), the reactions were heated under reflux using the alcohol as a solvent and excess of potassium hydroxide (Table 1, entries 1 and 2). For alcohols with higher boiling points, the reactions were performed with sodium hydride (Table 1, entries 3 and 4). For phenol, thiophenol and dialkylamines, heating with potassium carbonate in DMF gave good results (Table 1, entries 5–9). Finally, the reaction with potassium hydroxide was sluggish even under high temperature (Table 1, entry 10) and for amination, heating with aqueous ammonia solution in DMSO in a pressure vessel was required to form aniline **3k** (Table 1, entry 11).

**Table 1:** S_N_Ar reactions of **2**.

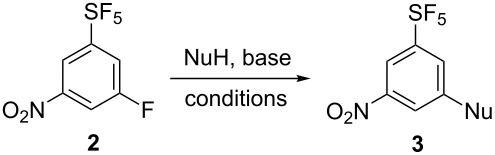						

Entry	NuH (equiv)	Base (equiv)	Solvent	Temp. (°C)	Time (h)	**3**, Yield (%)^a^

1	MeOH (excess)	KOH (5)	MeOH	80	0.5	**3a**, 85
2	EtOH (excess)	KOH (3)	EtOH	80	0.6	**3b**, 83
3	iPrOH (excess)	NaH (3)	iPrOH	rt	6	**3c**, 72
4	HC≡C-CH_2_OH (1.5)	NaH (3)	THF	rt	2	**3d**, 70
5	PhOH (1.5)	K_2_CO_3_ (3)	DMF	80	3	**3e**, 67
6	PhSH (1.5)	K_2_CO_3_ (3)	DMF	90	3	**3f**, 46
7	Morpholine (3)	K_2_CO_3_ (3)	DMF	85	7	**3g**, 63
8	Piperidine (3)	K_2_CO_3_ (3)	DMF	85	3	**3h**, 51
9	Pyrrolidine (3)	K_2_CO_3_ (3)	DMF	85	2	**3i**, 67
10	’OH’	KOH (5)	DMSO^b^	135	6	**3j**, 33
11	’NH_2_’	NH_4_OH^c^ (2.5)	DMSO	135	5	**3k**, 44

^a^Isolated yield. ^b^DMSO/H_2_O (2:1, v/v). ^c^28% aqueous ammonia solution.

It is well known that *ipso* attack of nitroaromatics by nucleophiles (S_N_Ar) is only a secondary process. Under kinetic conditions the aromatic system is initially attacked by a nucleophile in *ortho* or *para* position to the nitro group, which was exploited in oxidative nucleophilic substitution for hydrogen reactions (ONSH) with organolithium or magnesium species or in vicarious nucleophilic substitution reactions (VNS) with carbon, oxygen or nitrogen nucleophiles [29]. VNS is a very powerful process for selective alkylation, amination and hydroxylation of nitroaromatics and was reported to proceed efficiently on **1** and its *para*-isomer [19–21]. In general, the reactions are characterized by short reaction times, low temperatures and an equimolar amount of the nucleophile. The results of VNS reactions with compound **2** are shown in Table 2. With carbon nucleophiles the reactions proceeded in good yields except for diethyl chloromethylphosphonate. A very short reaction time was needed in the reaction with bromoform to avoid decomposition of the tribromomethyl anion to dibromocarbene (Table 2, entry 4). Direct hydroxylation with cumene hydroperoxide required the use of liquid ammonia as a co-solvent (Table 2, entry 5). For direct amination 1,1,1-trimethylhydrazinium iodide was used, which upon deprotonation with strong base provided the nitrogen nucleophile containing the leaving group (Me_3_N) (Table 2, entry 6). High regioselectivities were observed in all cases except for the cyanomethylation reaction (Table 2, entry 3). In general, regioselectivities in VNS reactions were higher than for analogous reactions of **1** which can be explained by the presence of additional steric hindrance of the fluorine substituent in **2**. It is important to note that under the VNS conditions and in the presence of strong nucleophiles and base (*t*-BuOK), fluorine atoms of **2** and **4** remained intact.

**Table 2:** VNS reactions of **2**.

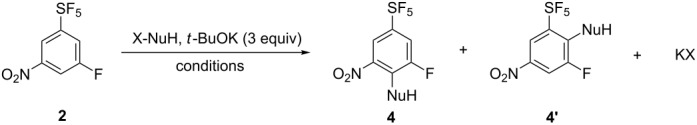							

Entry	X-NuH (equiv)	Solvent	Temp. (°C)	Time (min)	**4**, Yield (%)^a^		**4**:**4’**^b^

1	Cl-CH_2_CO_2_Et (1)	DMF	−30	10	**4a**, 71	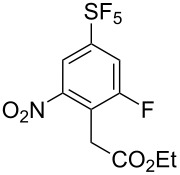	97:3
2	Cl-CH_2_PO_3_Et_2_ (1)	DMF	−60	10	**4b**, 31	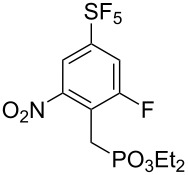	97:3^c^
3	PhO-CH_2_CN (1)	DMF	−30	10	**4c**, 50	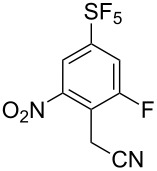	87:13
4	Br-CHBr_2_ (1.1)	DMF/THF^d^	−70	2	**4d**, 81	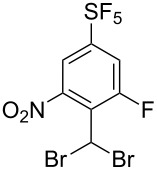	>98:2^c^
5	PhC(CH_3_)_2_O-OH (1)	NH_3_/THF^e^	−50	15	**4e**, 60	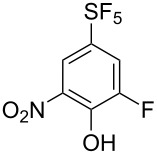	96:4
6^f^	I^−^ Me_3_N^+^-NH_2_ (1.8)	DMSO	rt	5	**4f**, 85	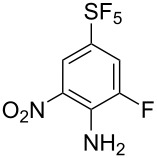	>98:2

^a^Isolated yield of the major isomer **4**. ^b^Determined by GC–MS of the crude reaction mixture. ^c^Determined by ^19^F NMR of the crude reaction mixture. ^d^DMF/THF (7:2, v/v). ^e^NH_3_/THF (4:1, v/v). ^f^Using *t*-BuOK (4 equiv).

## Conclusion

In conclusion, 3-fluoro-5-nitro-1-(pentafluorosulfanyl)benzene was prepared by direct fluorination and fluorodenitration pathways. It underwent nucleophilic aromatic substitutions of the fluorine atom with oxygen, sulfur and nitrogen nucleophiles affording novel 3-substituted-5-nitro-1-(pentafluorosulfanyl)benzenes. Regioselective vicarious nucleophilic substitution with carbon, oxygen, and nitrogen nucleophiles afforded 4-substituted-3-fluoro-5-nitro-1-(pentafluorosulfanyl)benzenes.

## Supporting Information

Synthesis and characterization of all products, copies of ^1^H, ^13^C, and ^19^F NMR spectra of newly synthesized products.

File 1 Experimental part and copies of NMR spectra.
