# Supplementary material for: Synthesis and nucleophilic aromatic substitution of 3-fluoro-5-nitro-1-(pentafluorosulfanyl)benzene
Source: Beilstein J Org Chem. 2016 Feb 3;12:192–7. doi: 10.3762/bjoc.12.21 (PMC4778532; doi:10.3762/bjoc.12.21)

Supporting Information

for

Synthesis and nucleophilic aromatic substitution of 3-fluoro-5-nitro-1-(pentafluorosulfanyl)benzene

Javier Ajenjo[1], Martin Greenhall[2], Camillo Zarantonello[2] and Petr Beier[1,]*[§]

Address: [1]Institute of Organic Chemistry and Biochemistry, v.v.i., Academy of Sciences of the Czech Republic, Flemingovo nám. 2, 166 10 Prague 6, Czech Republic and [2]F2 Chemicals Ltd, Lea Lane, Lea Town, Preston, PR4 0RZ, UK

*Corresponding author

[§]Tel.: +420 739002221; fax: +420 220183578

Email address: Petr Beier - beier@uochb.cas.cz

Experimental part and copies of NMR spectra

Table of Contents

General information	S1
Synthesis of 3-fluoro-5-nitro-1-(pentafluorosulfanyl)benzene (**2**)	S2
Synthesis of compounds **3** by S_NAr of fluorine	S3
Synthesis of compounds **4** by VNS	S7
Copies of NMR spectra	S11

General information

NMR spectra were recorded at 25 °C in $CDCl_3$ or acetone-d_6 on Bruker Avance 400 MHz or 500 MHz instruments. Chemical shifts (δ) are reported in ppm and referenced to residual signals of solvents or internal standards: $CHCl_3$ δ_H = 7.26, δ_C = 77.16; acetone-d_5 δ_H = 2.05, δ_C = 29.84; Me_4Si δ_H = 0.00; $CFCl_3$ δ_F = 0.0. Coupling constants (*J*) are given in Hertz. ^{13}C and ^{19}F NMR spectra were ^1H decoupled. GC–MS spectra were recorded on an Agilent 7890A gas chromatograph coupled with a 5975C quadrupole mass-selective electron impact

(EI) detector (70 eV). High-resolution mass spectra (HRMS) were recorded on an Agilent 7890A gas chromatograph coupled with a Waters GCT Premier orthogonal acceleration time-of-flight detector using electron impact (EI) or chemical (CI) ionizations, or on a LTQ Orbitrap XL using electrospray ionization (ESI). Elemental analyses were obtained using a Perkin–Elmer PE 2400 Series II CHNS. Purification of the products was performed by flash chromatography using silica gel 60. Isolated products had ≥95% purity as determined by ^{1}H, ^{13}C NMR or GCMS. The major impurity was aliphatic hydrocarbon compounds from hexane solvent. Such impurity is unavoidable. Dry solvents if used were obtained the following way: THF was freshly distilled over Na/benzophenone, DMF and MeCN were dried over activated 3 Å molecular sieves.

Synthesis of 3-fluoro-5-nitro-1-(pentafluorosulfanyl)benzene (2)

By direct fluorination of 1,2-bis(3-nitrophenyl)disulfane: 1,2-Bis(3-nitrophenyl)disulfane (2.50 kg, 8.1 mol) and dry MeCN (7.1 L) was charged into a 10 L reactor. The mixture was cooled to −10 °C and 10% F_2/N_2 (v/v) was introduced to the stirred solution at a rate of 300 L/h while keeping the temperature between −10 °C and −4 °C. After 68–95 h (10–14 equiv of F_2), N_2 was introduced (270 L/h) for 5 min. Similar reactions were combined, carefully quenched with water, steam distilled, and extracted into CH_2Cl_2. The solvent was removed under reduced pressure affording crude **1** (12.6 kg) containing 6% of **2** (by GC) which was charged to a 10 L vacuum distillation unit equipped with a 30 plate bubble cap column and warmed offtakes. After removal of solvent residue and other volatiles, fraction **2** (445 g, 2–3% yield, 88% purity by GC) was obtained at 81–85 °C, 3 mmHg, and reflux ratio of 4:1. Redistillation at 66 °C and 0.75 mmHg afforded **2** of 99% purity by GC.

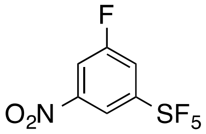

Pale yellow low-melting: ^{1}H NMR (400 MHz, $CDCl_3$) δ 8.48 (d, $^{4}J_{HF}$ = 1.4 Hz, 1H), 8.15 (dt, $^{4}J_{HH}$ = 7.5 Hz, $^{3}J_{HF}$ = 2.3 Hz, 1H), 7.86 (dt, $^{4}J_{HH}$ = 7.8 Hz, $^{3}J_{HF}$ = 2.1 Hz, 1H). ^{13}C NMR (101 MHz, $CDCl_3$) δ 161.68 (d, $^{1}J_{CF}$ = 256.3 Hz), 154.78 (quintd, $^{2}J_{CF}$ = 21.3 Hz, $^{3}J_{CF}$ = 7.5 Hz), 148.81 (d, $^{3}J_{CF}$ = 7.0 Hz), 120.30 (dquint, $^{2}J_{CF}$ = 26.2 Hz, $^{3}J_{CF}$ = 4.6 Hz), 117.84 (sextet, J_{CF} = 4.9 Hz), 114.76 (d, $^{2}J_{CF}$ = 26.1 Hz); ^{19}F NMR (377 MHz, $CDCl_3$) δ 80.03–78.27 (m, 1F), 62.44 (d, $^{2}J_{FF}$ = 151.2 Hz, 4F), –105.00 (s, 1F). HRMS (EI) *m/z* calcd for $C_6H_3F_6NO_2S$ $[M]^+$, 266.9789, found 266.9788.

By direct fluorination of 3-nitro-1-(pentafluorosulfanyl)benzene:

1. Reaction in MeCN followed by GC: A solution of **1** (4.45 g, 17.9 mmol) in MeCN (100 mL) cooled to −15 °C was fluorinated with 10% F_2/N_2 (v/v) at a rate of 3.6 L/h (at −15 to −10 °C). Samples were taken periodically and analyzed by GC to determine composition of

the mixture (see Figure 1, left). The crude mixture was poured onto ice, extracted with CH_2Cl_2 (3 × 60 mL), the combined organic phase was dried ($MgSO_4$) and solvent was removed under reduced pressure. The amount of tar (28% by weight) was determined by Kugelrohr distillation.

2. Reaction in anhydrous HF followed by GC: A solution of **1** (4.50 g, 18.1 mmol) in anhydrous HF (100 mL) cooled to −15 °C was fluorinated with 10% F_2/N_2 (v/v) at a rate of 3.6 L/h (at −15 to −10 °C). Samples were taken periodically and analyzed by GC to determine composition of the mixture (see Figure 1, right). The crude mixture after solvent evaporation was poured onto ice, extracted with CH_2Cl_2 (3 × 60 mL), the combined organic phase was dried ($MgSO_4$) and solvent was removed under reduced pressure. The amount of tar (5% by weight) was determined by Kugelrohr distillation.

Preparative reaction: A solution of **1** (2.509 g, 10.08 mmol) in MeCN (60 mL) was added to a nitrogen flushed PFA reactor with a magnetic stirring bar. A mixture of 20% F_2/N_2 (v/v) was bubbled for 11 h at a rate of 4.5 L/h while maintaining the temperature of the bath at –5 ºC. The mixture was flushed with N_2 for 10 min. and solvent was removed under reduced pressure yielding residue containing 40% of **2** (by GC–MS). Purification by flash chromatography (silica gel, Et_2O/petroleum ether, 10:90) afforded **2** (466.3 mg, 17% yield, 26% yield based on recovered **1**).

By fluorodenitration of 3,5-dinitro-1-(pentafluorosulfanyl)benzene: To a solution of TBAF·3H_2O (183 mg, 0.58 mmol, 1.5 equiv) in THF (5 mL) **5** (115 mg, 0.39 mmol, 1.0 equiv) was added. The mixture was stirred at 60 °C for 2 h. Then water (20 mL) was added and the product was extracted into EtOAc (3 × 15 mL). The combined organic phase was washed with water (15 mL), dried ($MgSO_4$) and solvent was removed under reduced pressure. Purification by flash chromatography (silica gel, Et_2O/hexane 4:96) afforded pure **2** as a pale yellow solid (57 mg, 56% yield).

Synthesis of compounds 3 by S_NAr of fluorine

General procedure 1: To a solution of KOH (138.1 mg, 2.46 mmol, 5 equiv.) in the corresponding alcohol (4 mL), **2** (130.0 mg, 0.49 mmol, 1 equiv.) was added. The mixture was stirred at 80 °C for 30 min. If necessary, aqueous solution of NaOH (0.5 M) was added to pH ≈ 8 and the product was extracted into Et_2O (4 × 10 mL). The combined organic phase was washed with water (15 mL), brine (15 mL), dried ($MgSO_4$) and solvent was removed under reduced pressure. Purification by flash chromatography afforded pure **3**.

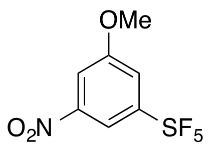

3a: Prepared according to the general procedure 1 using KOH (2.46 mmol) in MeOH (4 mL) and **2** (0.49 mmol) at 80 °C for 30 min. Purification by flash chromatography (silica gel, *n*-pentane/EtOAc, 90:10) afforded **3a** as a pale yellow solid (144 mg, 85% yield): mp 73–75 °C; ^{1}H NMR (400 MHz, $CDCl_3$) δ_H 8.20 (t, $^{4}J_{HH}$ = 1.9 Hz, 1H), 7.88 (t, $^{4}J_{HH}$ = 2.1 Hz, 1H), 7.60 (t, $^{4}J_{HH}$ = 2.5 Hz, 1H), 3.96 (s, 3H); ^{13}C NMR (101 MHz, $CDCl_3$) δ 160.25, 154.65 (quint, $^{2}J_{CF}$ = 19.7 Hz), 148.80, 119.07 (quint, $^{3}J_{CF}$ = 4.7 Hz), 113.79 (quint, $^{3}J_{CF}$ = 4.9 Hz), 111.23, 56.67; ^{19}F NMR (377 MHz, $CDCl_3$) δ 80.73 (quint, $^{2}J_{FF}$ = 149.0 Hz, 1F), 62.25 (d, $^{2}J_{FF}$ = 150.1 Hz, 4F); HRMS (EI) *m/z* calcd for $C_6H_3F_5NO_3S$ $[M]^+$ 278.9989, found 278.9990.

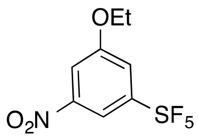

3b: Prepared according to the general procedure 1 using KOH (2.49 mmol) in EtOH (4 mL) and **2** (0.50 mmol) at 80 °C for 35 min. Purification by flash chromatography (silica gel, *n*-pentane/EtOAc, 90:10) afforded **3b** as a yellow oil (125 mg, 83% yield): ^{1}H NMR (400 MHz, $CDCl_3$) δ 8.19 (t, $^{4}J_{HH}$ = 1.9 Hz, 1H), 7.86 (t, $^{4}J_{HH}$ = 2.1 Hz, 1H), 7.59 (t, $^{4}J_{HH}$ = 2.2 Hz, 1H), 4.17 (q, $^{3}J_{HH}$ = 7.0 Hz, 2H), 1.49 (t, $^{3}J_{HH}$ = 7.0 Hz, 3H); ^{13}C NMR (101 MHz, $CDCl_3$) δ 159.60, 154.64 (quint, $^{2}J_{CF}$ = 19.8 Hz), 148.77, 119.52 (quint, $^{3}J_{CF}$ = 4.7 Hz), 113.61 (quint, $^{3}J_{CF}$ = 4.8 Hz), 111.60, 65.43, 14.52; ^{19}F NMR (377 MHz, $CDCl_3$) δ 81.69–79.99 (m, 1F), 62.24 (d, $^{2}J_{FF}$ = 150.7 Hz, 4F); HRMS (EI) *m/z* calcd for $C_8H_8F_5NO_3S$ $[M]^+$ 293.0145, found 293.0144.

General procedure 2: To a solution of NaH (3 equiv) in the appropriate solvent the corresponding alcohol was added and stirred for 30 min at rt. Then **2** (1 equiv) was added and the mixture was stirred at rt for the corresponding time. Water (5 mL) and aqueous solution of NaOH (10 mL, 0.5 M) were added and the product was extracted into Et_2O (4 × 20). The combined organic phase was washed with water, brine, dried ($MgSO_4$) and solvent was removed under reduced pressure. If necessary, purification by flash chromatography afforded pure **3**.

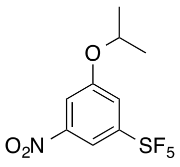

3c: Prepared according to the general procedure 2 from the mixture of NaH (26.0 mg, 0.65 mmol, 3 equiv) and dry iPrOH (2.5 mL) stirred for 30 min. Then **2** (50.0 mg, 0.19 mmol, 1 equiv) was added and the mixture was stirred at rt for 6 h. Crude product after extraction and solvent removal was of sufficient purity; **3c** was obtained as pale brown oil (42 mg, 72% yield): ^{1}H NMR (400 MHz, $CDCl_3$) δ 8.17 (td, $^{4}J_{HH}$ = 1.9, 0.9 Hz, 1H), 7.85 (t, $^{4}J_{HH}$ = 2.0 Hz, 1H), 7.57 (td, $^{4}J_{HH}$ = 2.2, 0.8 Hz, 1H), 4.69 (heptd, $^{3}J_{HH}$ = 6.0 Hz, $^{5}J_{HH}$ = 0.6 Hz, 1H), 1.41 (dd, $^{3}J_{HH}$ = 6.0 Hz, J = 1.0 Hz, 6H); ^{13}C NMR (101 MHz, $CDCl_3$) δ 158.63, 154.72 (quint, $^{2}J_{CF}$ = 20.0 Hz), 148.82, 120.49 (quint, $^{3}J_{CF}$ = 4.6 Hz), 113.39 (quint, $^{3}J_{CF}$ = 4.8 Hz), 112.43, 72.20, 21.73; ^{19}F NMR (377 MHz, $CDCl_3$) δ

81.95–79.77 (m, 1F), 62.18 (d, $^2J_{FF}$ = 150.9 Hz, 4F); HRMS (EI) *m/z* calcd for $C_9H_{10}F_5NO_3S$ $[M]^+$ 307.0302, found 307.0304.

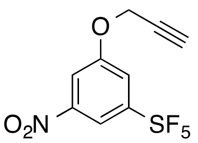

3d: Prepared according to the general procedure 2 using NaH (57.2 mg, 1.41 mmol, 3 equiv.) and propargyl alcohol (42.0 mg, 0.75 mmol, 1.5 equiv) in dry THF (3.5 mL). Then a solution of **2** (126.0 mg, 0.47 mmol, 1 equiv) in dry THF (1 mL) was added dropwise. The mixture was stirred at rt for 2 h. Purification of the product by flash chromatography (silica gel, hexane/EtOAc, 90:10) afforded **3d** as a pale orange oil (94.0 mg, 70% yield): ^{1}H NMR (400 MHz, $CDCl_3$) δ 8.27 (t, $^4J_{HH}$ = 1.9 Hz, 1H), 8.01 (t, $^4J_{HH}$ = 2.1 Hz, 1H), 7.70 (dd, $^4J_{HH}$ = 2.4, 1.9 Hz, 1H), 4.86 (d, $^4J_{HH}$ = 2.4 Hz, 2H), 2.64 (t, $^4J_{HH}$ = 2.4 Hz, 1H);^{13}C NMR (101 MHz, $CDCl_3$) δ 157.91, 154.64 (quint, $^3J_{CF}$ = 20.0 Hz), 148.71, 120.05 (quint,$^4J_{CF}$ = 4.8 Hz), 114.68 (quint, $^4J_{CF}$ = 4.9 Hz), 112.53, 77.92, 76.31, 57.10; ^{19}F NMR (377 MHz, $CDCl_3$) δ 81.37–79.62 (m, 1F), 62.27 (d, $^2J_{FF}$ = 150.9 Hz, 4F); HRMS (EI) *m/z* calcd for $C_9H_6F_5NO_3S$ $[M]^+$ 302.9989, found 302.9988.

General procedure 3: To a solution of K_2CO_3 (3 equiv) and the corresponding nucleophile (1.5 equiv) in DMF (3 mL), **2** (1 equiv) was added and the mixture was stirred at the appropriate temperature and time. Water (25 mL) was added and the product was extracted into EtOAc or *t*-BuOMe. The combined organic phase was washed with water, brine, dried ($MgSO_4$), and solvent was removed under reduced pressure. Purification by flash chromatography afforded **3**.

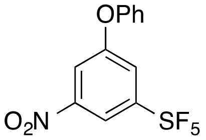

3e: Prepared according to the general procedure 3 using K_2CO_3 (286.0 mg, 2.07 mmol, 3 equiv), phenol (104.6 mg, 1.11 mmol, 1.5 equiv) and **2** (162.9 mg, 0.61 mmol, 1 equiv). The mixture was stirred at 80 °C for 3 h. Purification of the product by flash chromatography (silica gel, Et_2O/hexane, 5:95) afforded the product as a beige solid (139.7 mg, 67% yield): mp 57–59 °C; ^{1}H NMR (400 MHz, $CDCl_3$) δ 8.31 (t, $^4J_{HH}$ = 1.9 Hz, 1H), 7.88 (t, $^4J_{HH}$ = 2.1 Hz, 1H), 7.72 (t, $^4J_{HH}$ = 2.3 Hz, 1H), 7.52–7.44 (m, 2H), 7.34–7.28 (m, 1H), 7.14–7.06 (m, 2H); ^{13}C NMR (101 MHz, $CDCl_3$) δ 159.04, 155.07 (quint, $^2J_{CF}$ = 20.1 Hz), 154.48, 148.86, 130.85, 126.13, 121.49 (quint, $^4J_{CF}$ = 4.5 Hz), 120.22, 115.43 (quint, $^4J_{CF}$ = 5.0 Hz), 114.86; ^{19}F NMR (377 MHz, $CDCl_3$) δ 81.30–79.30 (m, 1F), 62.32 (d, $^2J_{FF}$ = 151.0 Hz, 4F); HRMS (EI) *m/z* calcd for $C_{12}H_8F_5NO_3S$ $[M]^+$ 341.0145, found 341.0144.

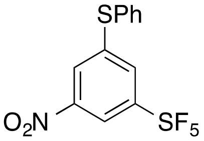

3f: Prepared according to the general procedure 3 using K_2CO_3 (153.0 mg, 1.11 mmol, 3 equiv) thiophenol (63.0 mg, 0.57 mmol, 1.5 equiv) and **2** (101.0 mg, 0.38 mmol, 1 equiv). The mixture was stirred at 90 °C for 3 h. Purification by flash chromatography (silica gel $CHCl_2$/petroleum ether, 2:98) afforded **3f** as

a pale yellow oil (62.5 mg, 46% yield): ^{1}H NMR (400 MHz,) δ 8.33 (t, $^{4}J_{HH}$ = 2.0 Hz, 1H), 8.03 (t, $^{4}J_{HH}$ = 1.8 Hz, 1H), 7.79 (t, $^{4}J_{HH}$ = 1.8 Hz, 1H), 7.58–7.46 (m, 5H); ^{13}C NMR (101 MHz, $CDCl_3$) δ 154.83–153.84 (m), 148.34, 143.88, 134.59, 130.54, 130.36, 129.88, 129.72 (quint, $^{3}J_{CF}$ = 4.7 Hz), 124.43, 118.44 (quint, $^{3}J_{CF}$ = 4.9 Hz); ^{19}F NMR (377 MHz, $CDCl_3$) δ 81.28–79.29 (m, 1F), 62.34 (d, $^{2}J_{FF}$ = 151.2 Hz, 4F); HRMS (EI) *m/z* calcd for $C_{12}H_8F_5NO_2S_2$ $[M]^+$ 356.9917, found 356.9914.

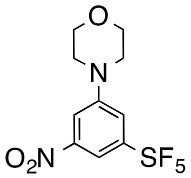

3g: Prepared according to the general procedure 3 using K_2CO_3 (149.0 mg, 1.08 mmol, 3 equiv), morpholine (99 mg, 1.14 mmol, 3 equiv) and **2** (97.5 mg, 0.36 mmol, 1 equiv). The mixture was stirred at 85 °C for 7 h. Purification by flash chromatography (silica gel, hexane) afforded **3g** as a pale yellow oil (79.0 mg, 63% yield): ^{1}H NMR (400 MHz, $CDCl_3$) δ 8.04 (t, $^{4}J_{HH}$ = 1.9 Hz, 1H), 7.83 (t, $^{4}J_{HH}$ = 2.1 Hz, 1H), 7.49 (t, $^{4}J_{HH}$ = 2.2 Hz, 1H), 3.95–3.85 (m, 4H), 3.93–3.85 (m, 4H); ^{13}C NMR (101 MHz, $CDCl_3$) δ 155.48–154.73 (m), 151.90, 148.86, 117.39 (quint, $^{3}J_{CF}$ = 4.5 Hz), 111.73, 111.58 (quint, $^{3}J_{CF}$ = 4.6 Hz), 66.43, 48.22; ^{19}F NMR (377 MHz, $CDCl_3$) δ 82.50–80.78 (m, 1F), 62.05 (d, $^{4}J_{HH}$ = 150.5 Hz, 4F); HRMS (EI) *m/z* calcd for $C_6H_4F_5NO_3S$ $[M]^+$ 334.0411, found 334.0410.

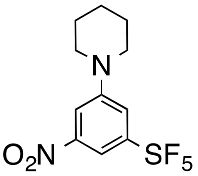

3h: Prepared according to the general procedure 3 using K_2CO_3 (145.0 mg, 1.12 mmol, 3 equiv), piperidine (99.0 mg, 1.12 mmol, 3 equiv) and **2** (107.0 mg, 0.36 mmol, 1 equiv). The mixture was stirred at 85 °C for 3 h. Purification of the product was carried out by flash chromatography (silica gel, *n*-pentane) afforded **3h** as a pale yellow solid (75.0 mg, 51% yield): mp 79–81 °C; ^{1}H NMR (400 MHz, $CDCl_3$) δ 7.93 (t, $^{4}J_{HH}$ = 1.9 Hz, 1H), 7.80 (t, $^{4}J_{HH}$ = 2.2 Hz, 1H), 7.47 (t, $^{4}J_{HH}$ = 2.2 Hz, 1H), 3.36–3.29 (m, 4H), 1.80–1.70 (m, 4H), 1.67 (dtt, $^{3}J_{HH}$ = 5.6, 4.3 Hz, $^{4}J_{HH}$ = 2.2 Hz, 2H); ^{13}C NMR (101 MHz, $CDCl_3$) δ 156.01–153.88 (m), 152.21, 148.88, 117.63 (t, $^{3}J_{CF}$ = 4.7 Hz), 111.89, 110.11 (quint, $^{3}J_{CF}$ = 5.1 Hz), 49.53, 25.35, 23.99; ^{19}F NMR (377 MHz, $CDCl_3$) δ 82.96–81.32 (m, 1F), 61.96 (d, $^{2}J_{FF}$ = 150.4 Hz, 4F); HRMS (EI) *m/z* calcd for $C_{11}H_{13}F_5N_2O_2S$ $[M]^+$ 332.0618, found 332.0619.

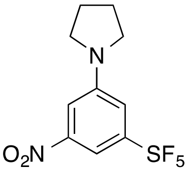

3i: Prepared according to the general procedure 3 using K_2CO_3 (150.0 mg, 1.12 mmol, 3 equiv), pyrrolidine (85.0 mg, 1.12 mmol, 3 equiv) and **2** (101.0 mg, 0.38 mmol, 1 equiv). The mixture was stirred at 85 °C for 2 h. No further purification was carried out, affording **3i** as a yellow solid (82.0 mg, 67% yield): mp 155–157 °C; ^{1}H NMR (400 MHz, $CDCl_3$) δ 7.81 (t, $^{4}J_{HH}$ = 1.9 Hz, 1H), 7.43 (t, $^{4}J_{HH}$ = 2.2 Hz, 1H), 7.10 (t, $^{4}J_{HH}$ = 2.1 Hz, 1H), 3.46 (ddd, $^{3}J_{HH}$ = 6.6, 4.3 Hz, $^{4}J_{HH}$ = 2.6 Hz, 4H), 2.13–2.09 (m, 4H); ^{13}C NMR (101 MHz, $CDCl_3$) δ 155.67–155.12 (m), 149.85,

149.38, 114.31 (quint, ${}^3J_{CF}$ = 4.8 Hz), 109.14, 106.83 (quint, ${}^3J_{CF}$ = 5.0 Hz), 48.94, 26.18; ^{19}F NMR (377 MHz, $CDCl_3$) δ 83.30–81.61 (m, 1F), 61.99 (d, ${}^2J_{FF}$ = 150.3 Hz, 4F); HRMS (EI) *m/z* calcd for $C_{10}H_{11}F_5N_2O_2S$ $[M]^+$ 318.0461, found 318.0460.

General procedure 4: A solution of **2** (1 equiv) and base in DMSO and water was heated at 135 ºC. Water (25 mL) was added and the product was extracted into EtOAc or Et_2O. The combined organic phase was washed with water (30 mL), brine (30 mL), dried ($MgSO_4$), and solvent was removed under reduced pressure. Purification by flash chromatography afforded **3j** and **3k**.

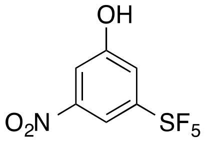

3j: Prepared according to the general procedure 4 using KOH (1.143 g, 18.72 mmol, 5 equiv) and **2** (1.006 g, 3.76 mmol, 1 equiv) in DMSO (6 mL) and water (3 mL). The mixture was stirred at 135 °C for 6 h, then cooled and aqueous HCl (1M) was added to pH ~ 3. Purification by flash chromatography (silica gel, AcOH/EtOAc/$CHCl_3$/hexane, 1:40:40:19) afforded **3j** as a yellow solid (336.3 mg, 33% yield): mp 89–91 °C; ^{1}H NMR (400 MHz, $CDCl_3$) δ 8.22 (t, ${}^4J_{HH}$ = 1.9 Hz, 1H), 7.88 (t, ${}^4J_{HH}$ = 2.1 Hz, 1H), 7.65–7.55 (m, 1H), 6.07 (s, 1H); ^{13}C NMR (101 MHz, $CDCl_3$) δ 156.52, 154.5–155.2 (m), 148.68, 119.84 (quint, ${}^3J_{CF}$ = 4.6 Hz), 114.10 (quint, ${}^3J_{CF}$ = 4.9 Hz), 113.76; ^{19}F NMR (377 MHz, $CDCl_3$) δ 83.69–78.92 (m, 1F), 62.25 (d, ${}^2J_{FF}$ = 150.6 Hz, 4F); HRMS (CI) *m/z* calcd for $C_6H_4F_5NO_3S$ $[M + H]^+$ 265.9910, found 265.9908.

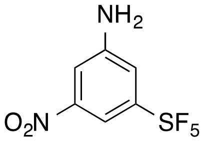

3k: Prepared according to the general procedure 4 using aqueous ammonia solution (28%, 0.1 mL, 1.31 mmol, 2.5 equiv) and **2** (149.8 mg, 0.56 mmol, 1 equiv) in DMSO (0.5 mL) in a sealed vial stirred at 135 °C for 5 h. Purification by flash chromatography (silica gel, EtOAc/hexane, 9:1) afforded the product **3k** a yellow oil (65.3 mg, 44% yield): ^{1}H NMR (400 MHz, acetone-d_6) δ 7.74 (d, ${}^4J_{HH}$ = 2.1 Hz, 2H), 7.55 (t, ${}^4J_{HH}$ = 2.1 Hz, 1H), 5.94 (s, 2H); ^{13}C NMR (126 MHz, acetone-d_6) δ 155.14 (quint, ${}^2J_{CF}$ = 18.1 Hz), 151.51, 149.81, 116.89 (quint, ${}^3J_{CF}$ = 4.7 Hz), 111.58, 108.37 (quint, ${}^3J_{CF}$ = 5.1 Hz); ^{19}F NMR (377 MHz, acetone-d_6) δ 85.29–82.19 (m, 1F), 62.77 (d, ${}^2J_{FF}$ = 148.8 Hz, 4F); HRMS (EI) *m/z* calcd for $C_6H_5F_5N_2O_2S$ $[M]^+$ 263.9992, found 263.9993.

Synthesis of compounds 4 by VNS

General procedure 5: To a solution of *t*-BuOK (3 equiv) in the corresponding dry solvent, a mixture of the appropriate X-NuH (1–2 equiv) and **2** (1 equiv) were added dropwise. After stirring for appropriate temperature and time, aqueous solution of HCl (1 M) was added to pH ~ 3 and the product was extracted into EtOAc (4 × 10 mL). The combined organic phase was

washed with LiCl (40 mL, 1 M), dried ($MgSO_4$), and the solvent was removed under reduced pressure. Purification by flash chromatography afforded product **4**.

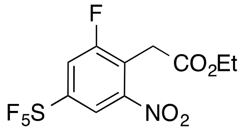

4a: Prepared according to the general procedure 5 using *t*-BuOK (77.0 mg, 0.69 mmol, 3 equiv) in DMF (3 mL) and a mixture of **2** (50.4 mg, 0.19 mmol, 1 equiv) and $ClCH_2CO_2Et$ (25.9 mg, 0.21 mmol, 1 equiv) in DMF (1.5 mL) at −30 °C for 10 min. No further purification of the product was carried out giving **4a** as a pale yellow oil (47.5 mg, 71% yield): ^{1}H NMR (400 MHz, $CDCl_3$) δ 8.32 (t, J = 2.0 Hz, 1H), 7.81 (dd, $^3J_{HH}$ = 8.8 Hz, $^4J_{HH}$ = 2.2 Hz, 1H), 4.21 (q, $^3J_{HH}$ = 7.1 Hz, 2H), 4.11 (d, $^4J_{HH}$ = 1.4 Hz, 2H), 1.28 (t, $^3J_{HH}$ = 7.1 Hz, 3H); ^{13}C NMR (101 MHz, $CDCl_3$) δ 168.16, 160.47 (d, $^1J_{CF}$ = 253.8 Hz), 152.75 (quintd, $^2J_{CF}$ = 21.7 Hz, $^3J_{CF}$ = 9.3 Hz), 149.50–149.15 (m), 122.79 (d, J = 18.9 Hz), 119.28–119.02 (m), 119.09–118.46 (m), 62.13, 31.08 (d, $^3J_{CF}$ = 4.5 Hz), 14.21; ^{19}F NMR (377 MHz, $CDCl_3$) δ 80.38–78.13 (m, 1F), 62.36 (d, $^2J_{FF}$ = 151.4 Hz, 4F), –108.46 (s, 1F); HRMS (EI) *m/z* calcd for $C_{10}H_9F_6NO_4S$ $[M]^+$ 353.0156, found 353.0158.

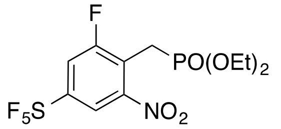

4b: Prepared according to the general procedure 5 using *t*-BuOK (186.7 mg, 1.66 mmol, 3 equiv) in DMF (4 mL) and a mixture of **2** (157.3 mg, 0.59 mmol, 1 equiv) and $ClCH_2PO_3Et_2$ (105.3 mg, 0.56 mmol, 1 equiv) in DMF (1 mL) −60 °C for 10 min. Purification by flash chromatography (silica gel, EtOAc/hexane 50:50) afforded **4b** as a beige solid (57.64 mg, 31% yield): mp 94–96 °C; ^{1}H NMR (400 MHz, $CDCl_3$) δ 8.19–8.16 (m, 1H), 7.75 (ddd, $^3J_{HF}$ = 9.1 Hz, $^4J_{HH}$ = 2.3 Hz, $^4J_{HF}$ = 0.9 Hz, 1H), 4.14–4.01 (m, 4H), 3.81 (dd, $^2J_{HP}$ = 22.9 Hz, $^4J_{HF}$ = 1.7 Hz, 2H), 1.26 (td, $^3J_{HH}$ = 7.1 Hz, $^4J_{HP}$ = 0.5 Hz, 6H); ^{13}C NMR (101 MHz, $CDCl_3$) δ 160.10 (dd, $^1J_{CF}$ = 254.7 Hz, $^3J_{CP}$ = 6.1 Hz), 152.59–151.80 (m), 149.73–149.12 (m), 121.30 (dd, $^2J_{CF}$ = 19.5 Hz, $^2J_{CP}$ = 10.7 Hz), 119.39–119.03 (m), 118.51–117.97 (m), 62.95 (d, $^2J_{CP}$ = 6.6 Hz), 23.19 (dd, $^1J_{CP}$ = 137.3 Hz, $^3J_{CF}$ =3.6 Hz), 16.29 (d, $^3J_{CP}$ = 6.1 Hz); ^{19}F NMR (376 MHz, $CDCl_3$) δ 80.44–78.47 (m, 1F), 62.46 (d, $^2J_{FF}$ = 151.5 Hz, 4F), –107.06 (d, $^4J_{FP}$ = 4.6 Hz, 1F); HRMS (ESI) *m/z* calcd for $C_{11}H_{14}F_6NO_5PS$ $[M + Na]^+$ 440.01263, found 440.01267.

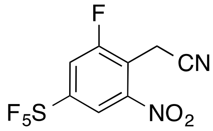

4c: Prepared according to the general procedure 5 using *t*-BuOK (186.9 mg, 1.67 mmol, 3 equiv) in DMF (3.5 mL) and a mixture of **2** (149.0 mg, 0.56 mmol, 1 equiv) and $PhOCH_2CN$ (79.6 mg, 0.60 mmol, 1 equiv) in DMF (2 mL) at –30 °C for 10 min. Purification flash chromatography (silica gel, Et_2O/hexane, 70:30) afforded **4c** as an orange oil (86.3 mg, 50% yield): ^{1}H NMR (400 MHz, $CDCl_3$) δ 8.41 (t, $^4J_{HH}$ = 2.0 Hz, 1H), 7.91 (dd, $^3J_{HF}$ = 8.8 Hz, $^4J_{HH}$ = 2.2 Hz, 1H), 4.15 (d, $^4J_{HF}$ = 1.4 Hz, 2H); ^{13}C NMR (101

MHz, $CDCl_3$) δ 160.24 (d, $^1J_{CF}$ = 257.0 Hz), 159.39–153.65 (m), 148.45, 120.5–119.71 (m), 119.60 (quint, $^3J_{CF}$ = 4.6 Hz), 118.61 (d, $^2J_{CF}$ =18.5 Hz), 114.11, 14.73 (d, $^4J_{CF}$ = 5.9 Hz); ^{19}F NMR (377 MHz, $CDCl_3$) δ 79.25–77.19 (m, 1F), 62.30 (d, $^2J_{FF}$ = 150.5 Hz, 4F), –106.40 (s, 1F); HRMS (EI) *m/z* calcd for $C_8H_4F_6N_2O_2S$ $[M]^+$ 305.9898, found 305.9896.

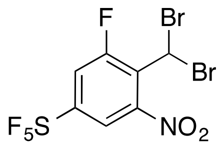

4d: Prepared according to the general procedure 5 using *t*-BuOK (133.8 mg, 1.19 mmol, 3 equiv) in DMF (3.5 mL) and a mixture of **2** (103.2 mg, 0.39 mmol, 1 equiv) and $CHBr_3$ (108.6 mg, 0.43 mmol, 1.1 equiv) in THF (1 mL) at −70 °C for 2 min. Purification by flash chromatography (silica gel, Et_2O/hexane, 2:98) afforded **4d** as a white solid (131.0 mg, 81% yield): mp 87–89 °C; ^{1}H NMR (500.0 MHz, $CDCl_3$) δ 8.11 (dd, $^4J_{HH}$ = 2.3 Hz, $^5J_{HF}$ = 1.7 Hz, 1H), 7.87 (dd, $^3J_{HF}$ = 10.1 Hz, $^4J_{HH}$ = 2.3 Hz, 1H), 7.20 (d, $^4J_{HH}$ = 3.2 Hz, $^4J_{HF}$ = 0.5 Hz, 1H); ^{13}C NMR (125.7 MHz, $CDCl_3$) δ 162.77–159.84 (m), 154.40–153.74 (m), 145.69–145.43 (m), 127.57 (d, $^2J_{CF}$ = 12.5 Hz), 120.26 (dquint, $^2J_{CF}$ = 26.7 Hz, $^3J_{CF}$ = 4.7 Hz), 118.39 (quint, $^3J_{CF}$ = 4.8 Hz), 22.71 (d, $^3J_{CF}$ = 3.8 Hz);^{19}F NMR (377 MHz, $CDCl_3$) δ 80.29–78.57 (m, 1F), 62.46 (d, $^2J_{FF}$ = 151.6 Hz, 4F), –107.06 (d, $^4J_{HF}$ = 4.6 Hz, 1F). Anal. Calcd. (%) for $C_7H_3Br_2F_6NO_2S$: C 19.15, H 0.69, Br 36.41, F 25.97, N 3.19, S 7.30, found: C 19.35, H 0.76, Br 36.29, F 23.49, N 2.90, S 7.25.

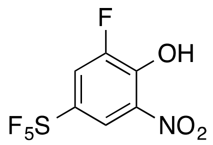

4e: To a solution of *t*-BuOK (199.5 mg, 1.78 mmol, 3 equiv) in liquid NH_3 (4 mL) cooled to −50 °C a mixture of **2** (158.5 mg, 0.59 mmol, 1 equiv) and cumene hydroperoxide (87.6 mg, 0.58 mmol, 1 equiv) in THF (1 mL) was added dropwise. After stirring for 15 min at −50 °C aqueous solution of HCl (1 M) was added to pH ~ 3 and the product was extracted into EtOAc (4 × 10 mL). The combined organic phase dried ($MgSO_4$), and the solvent was removed under reduced pressure. HCl (36%, 5 mL) was added and the mixture was stirred at 60 °C for 30 min. Water (10 mL) was added and the product was extracted into EtOAc (3 × 10 mL). The combined organic phase dried ($MgSO_4$), and the solvent was removed under reduced pressure. Purification by flash chromatography (silica gel, hexane/EtOAc/AcOH (80:15:5 to 95:0:5) afforded **4e** as yellow oil (101.6 mg, 60% yield): mp 79–81 °C; ^{1}H NMR (500 MHz, acetone-d_6) δ 8.14 (dd, $^4J_{HH}$ = 3.1 Hz, $^4J_{HF}$ = 1.8 Hz, 1H), 7.40 (dd, $^3J_{HF}$ = 11.4 Hz, $^4J_{HH}$ = 3.1 Hz, 1H); ^{13}C NMR (126 MHz, acetone-d_6) δ 160.60 (d, $^2J_{CF}$ = 18.2 Hz), 157.62 (d, $^1J_{CF}$ = 245.7 Hz), 135.75, 133.25–132.91 (m), 121.77, 115.42 (d, $^2J_{CF}$ = 25.5 Hz); ^{19}F NMR (377 MHz, acetone-d_6) δ 88.43 (quint, $^2J_{FF}$ = 149.2 Hz, 1F), 65.89 (d, $^2J_{FF}$ = 148.9 Hz, 4F), –129.64 (s, 1F); HRMS (EI) *m/z* calcd for $C_6H_3F_6NO_3S$ $[M]^+$ 282.9738, found 282.9739.

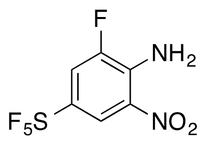

4f: To a solution of *t*-BuOK (256.4 mg, 2.38 mmol, 4 equiv) in DMSO (4 mL) a mixture of **2** (159.0 mg, 0.60 mmol, 1 equiv) and I^- Me_3N^+-NH_2 (225.1 mg, 1.11 mmol, 1.8 equiv) in DMSO (1 mL) was added dropwise at rt. After stirring for 5 min, NaOH (0.5 M, 20 mL) was added and the product was extracted into EtOAc (4 × 15 mL). The combined organic phase dried ($MgSO_4$), and the solvent was removed under reduced pressure. Purification by flash chromatography (silica gel, EtOAc/hexane, 10:90) afforded **4f** as yellow solid (143 mg, 85% yield): mp 78–80 °C; ^{1}H NMR (400 MHz, $CDCl_3$) δ 8.42 (t, $^4J_{HH}$ = 2.2 Hz, 1H), 7.63 (dd, $^3J_{HF}$ = 10.8 Hz, $^4J_{HH}$ = 2.5 Hz, 1H), 6.49 (s, 2H); ^{13}C NMR (101 MHz, $CDCl_3$) δ 150.50 (d, $^1J_{CF}$ = 247.0 Hz), 140.68–139.14 (m), 137.23 (d, $^2J_{CF}$ = 15.5 Hz), 130.90–130.85 (m), 120.65–120.36 (m), 117.46 (dquint, $^3J_{CF}$ = 23.7 Hz, $^4J_{CF}$ =4.3 Hz); ^{19}F NMR (377 MHz, $CDCl_3$) δ 83.26–81.62 (m, 1F), 63.70 (d, $^2J_{FF}$ = 151.3 Hz, 4F), –129.50 (s, 1F; HRMS (EI) *m/z* calcd for $C_6H_4F_6N_2O_2S$ $[M]^+$ 281.9898, found 281.9899.

Copies of NMR spectra

2: ^{1}H NMR (400 MHz, $CDCl_3$)

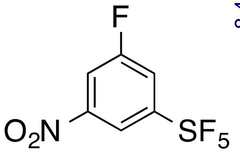

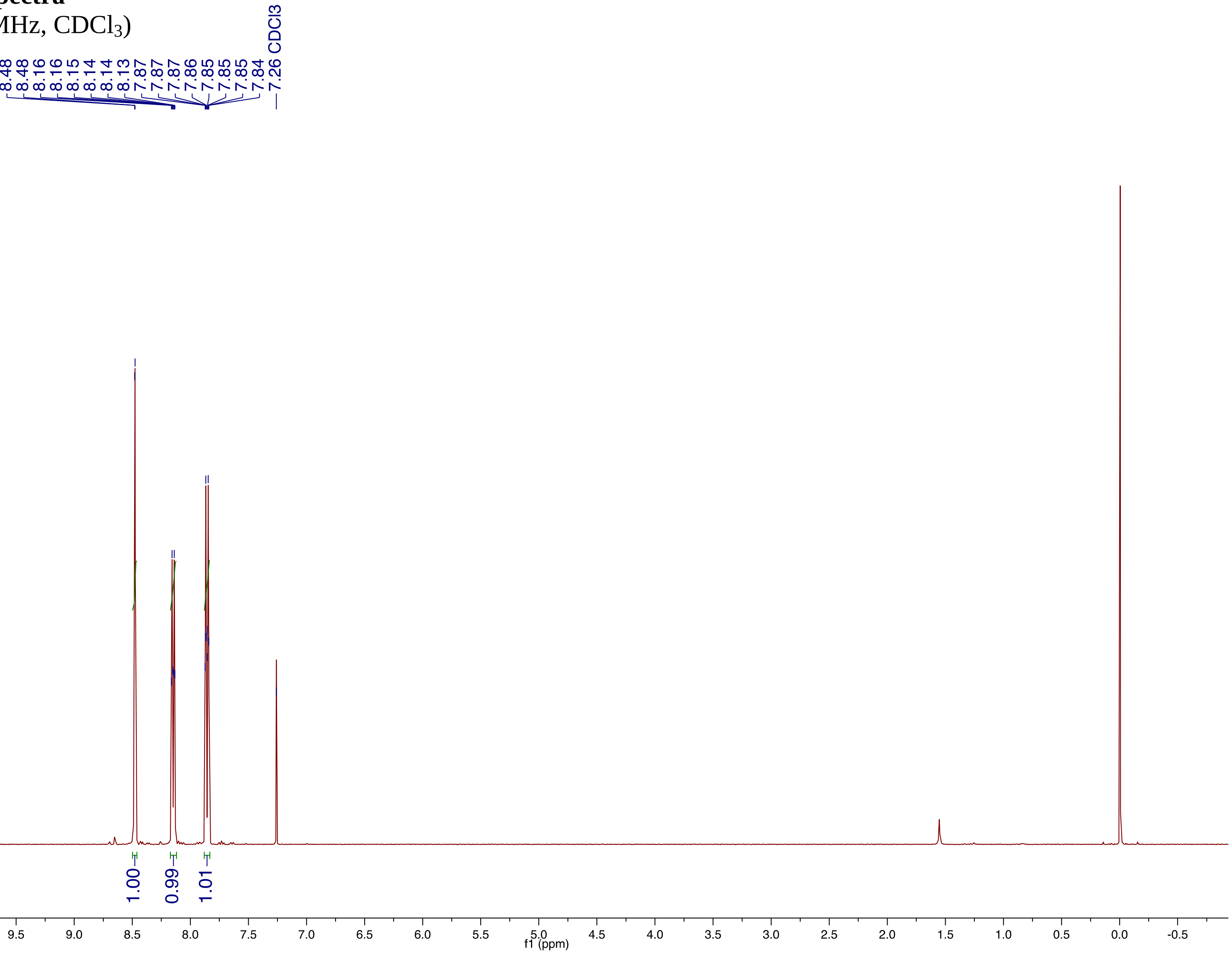

2: ^{13}C NMR (101 MHz, $CDCl_3$)

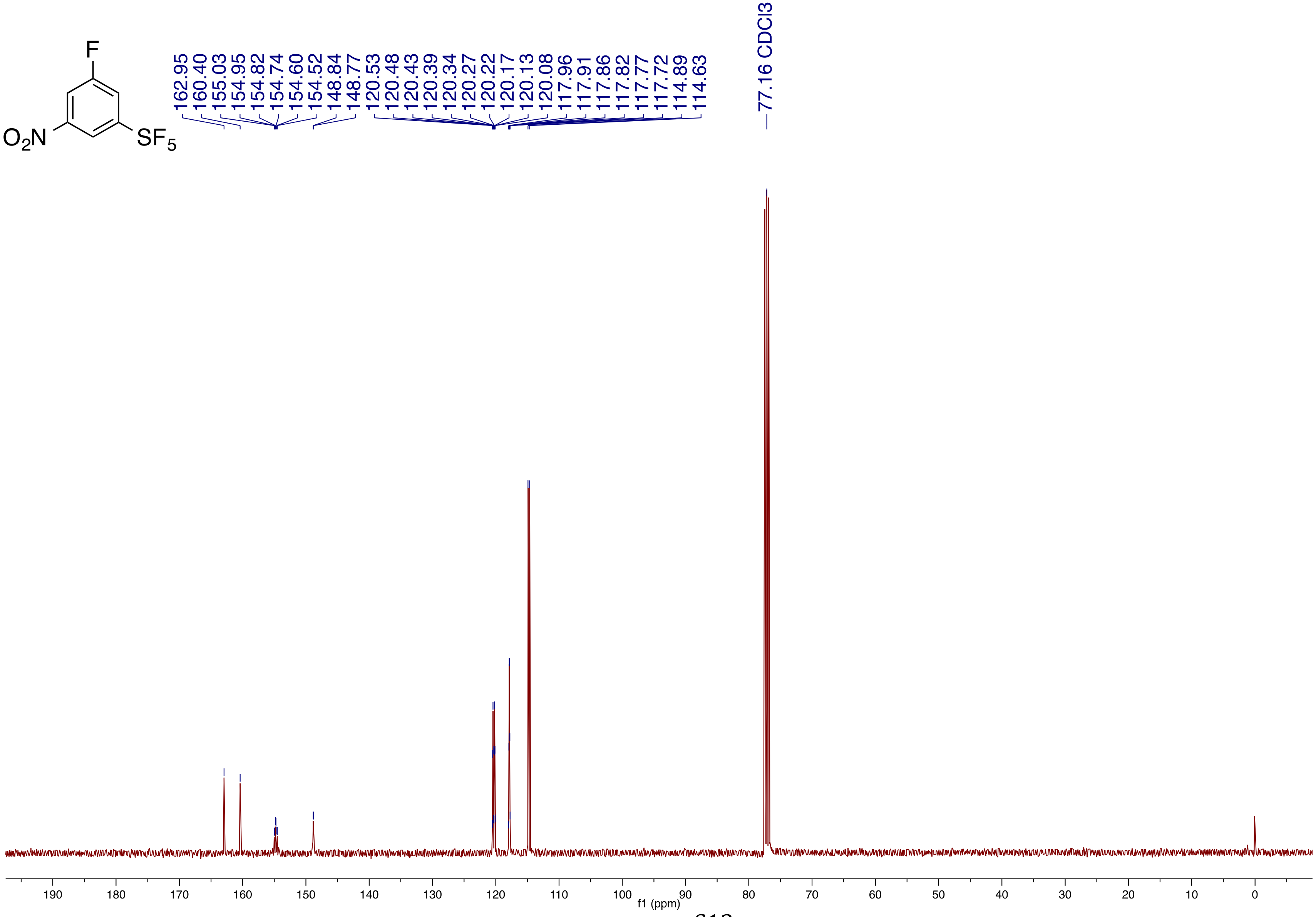

2: ^{19}F NMR (377 MHz, $CDCl_3$)

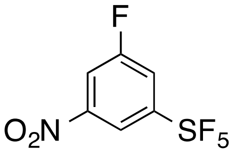

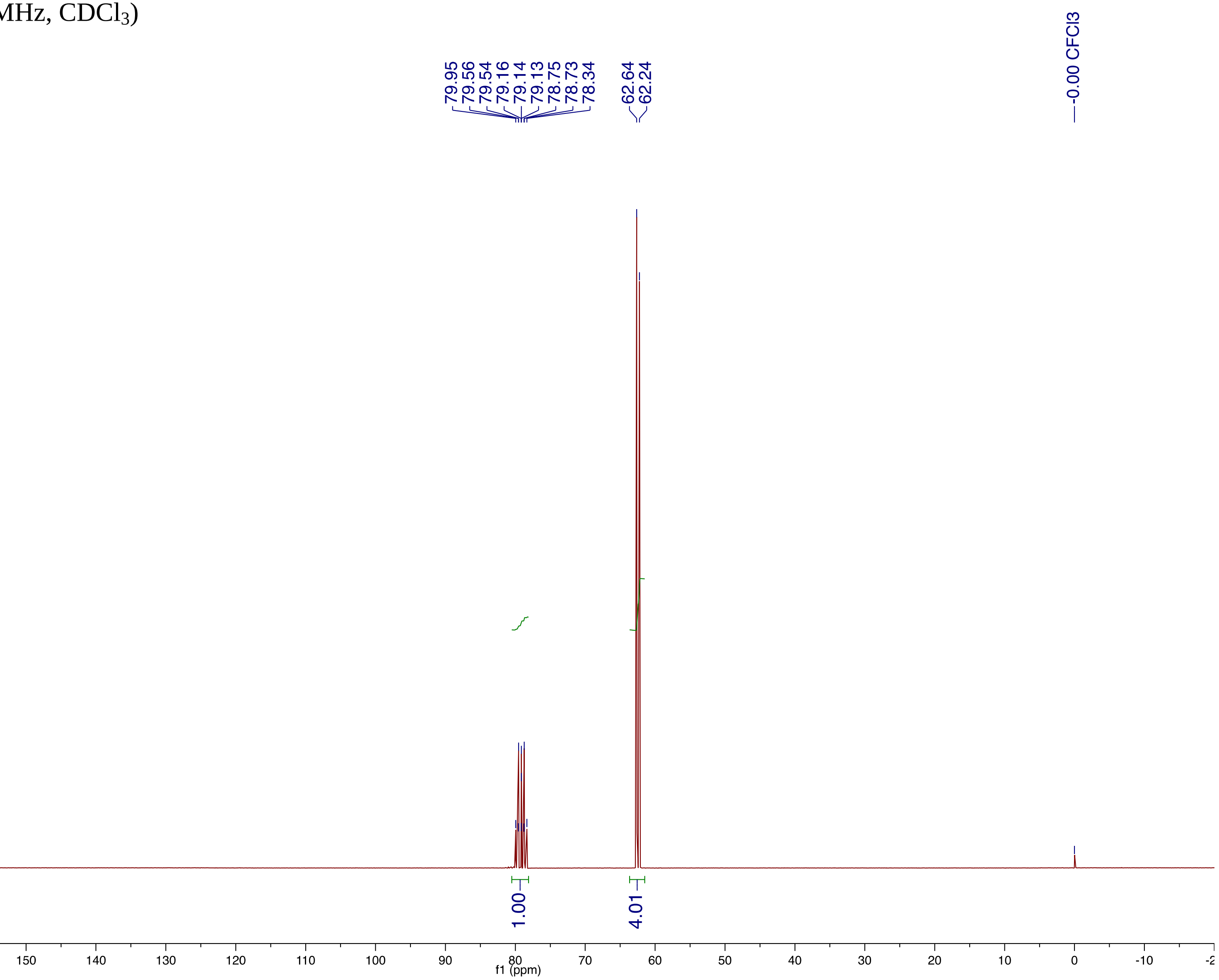

2: ^{19}F NMR (377 MHz, $CDCl_3$)

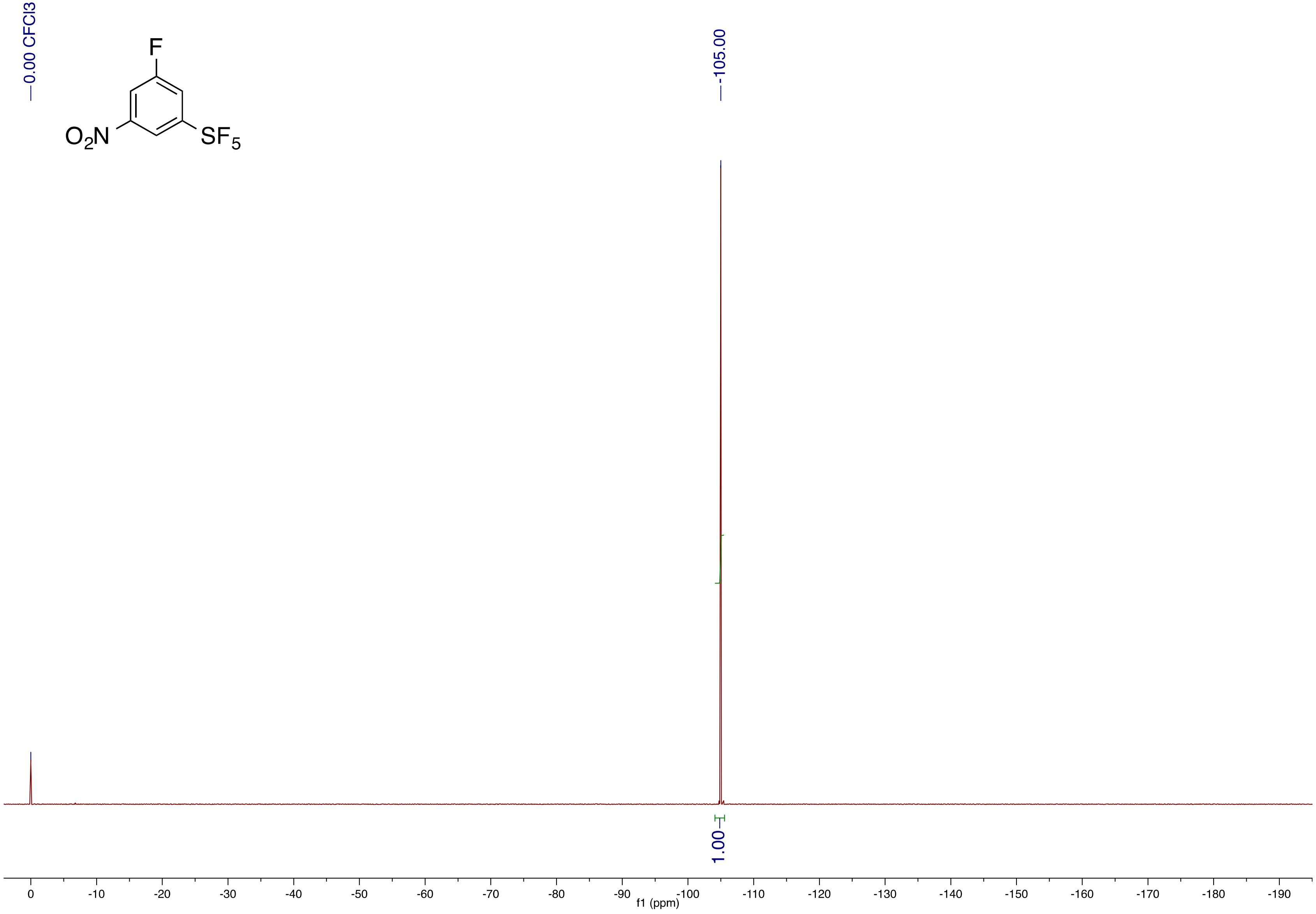

3a: ^{1}H NMR (400 MHz, $CDCl_3$)

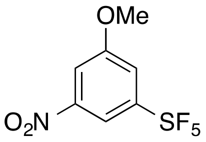

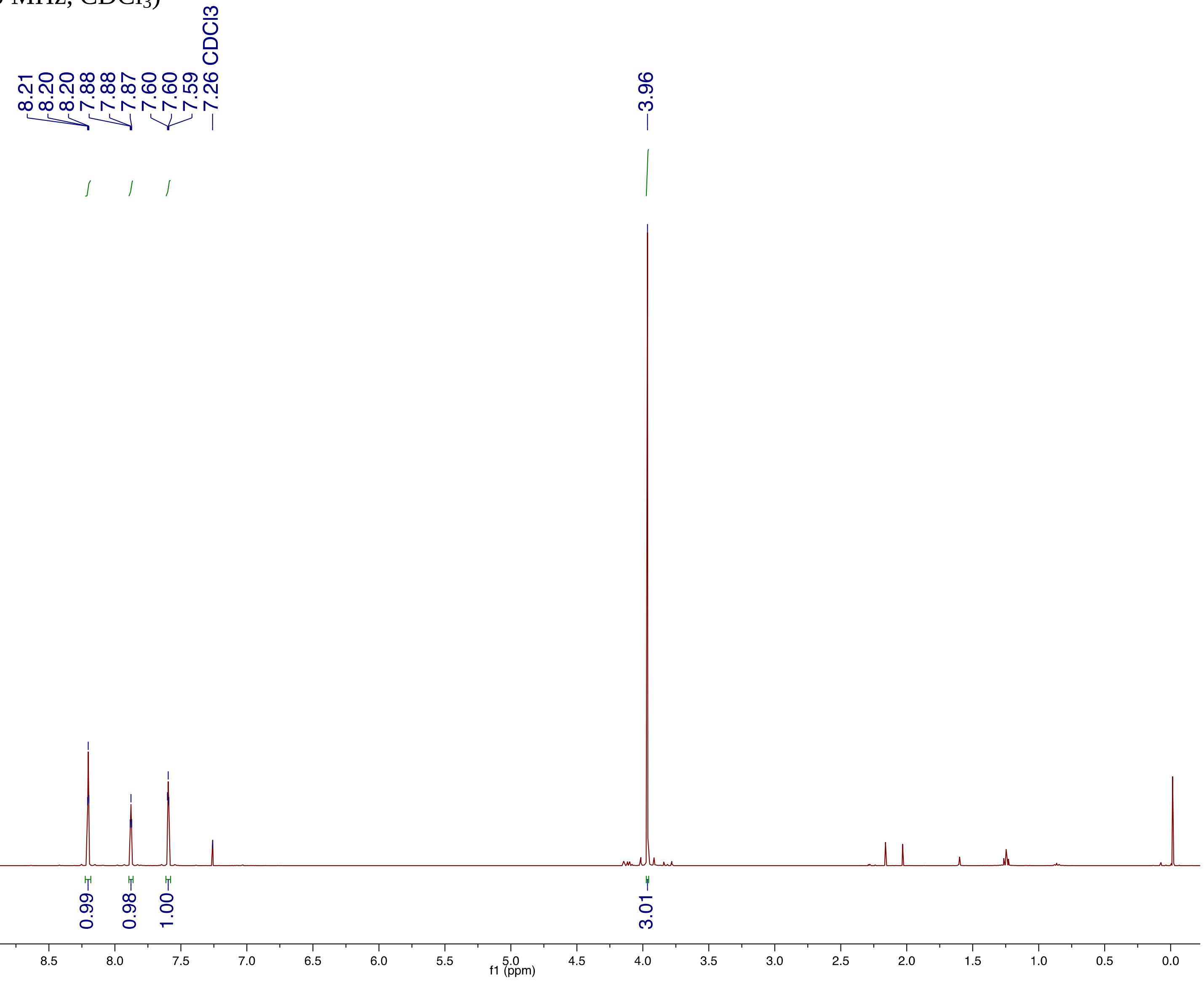

3a: ^{13}C NMR (101 MHz, $CDCl_3$)

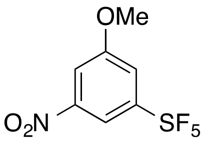

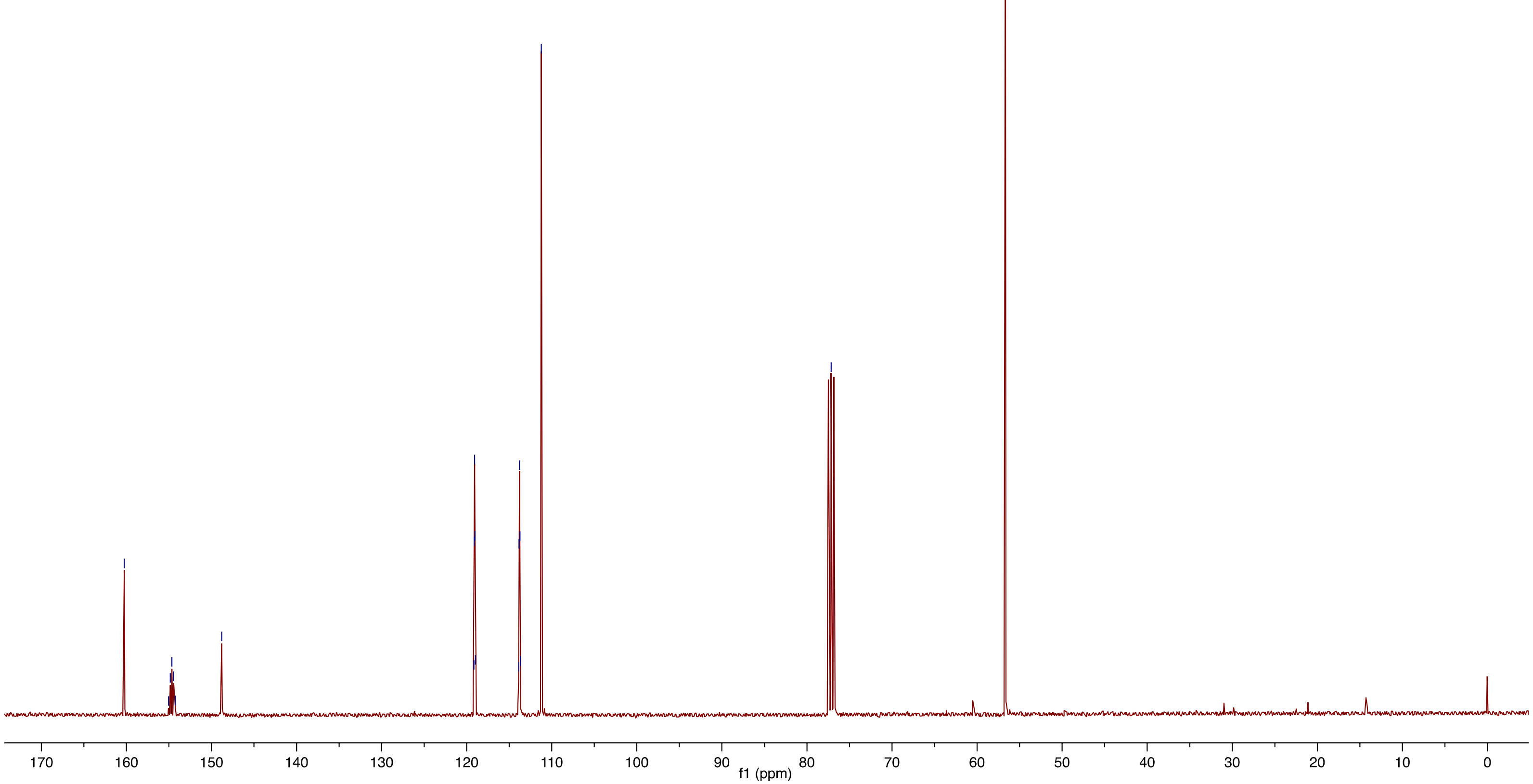

3a: ^{19}F NMR (377 MHz, $CDCl_3$)

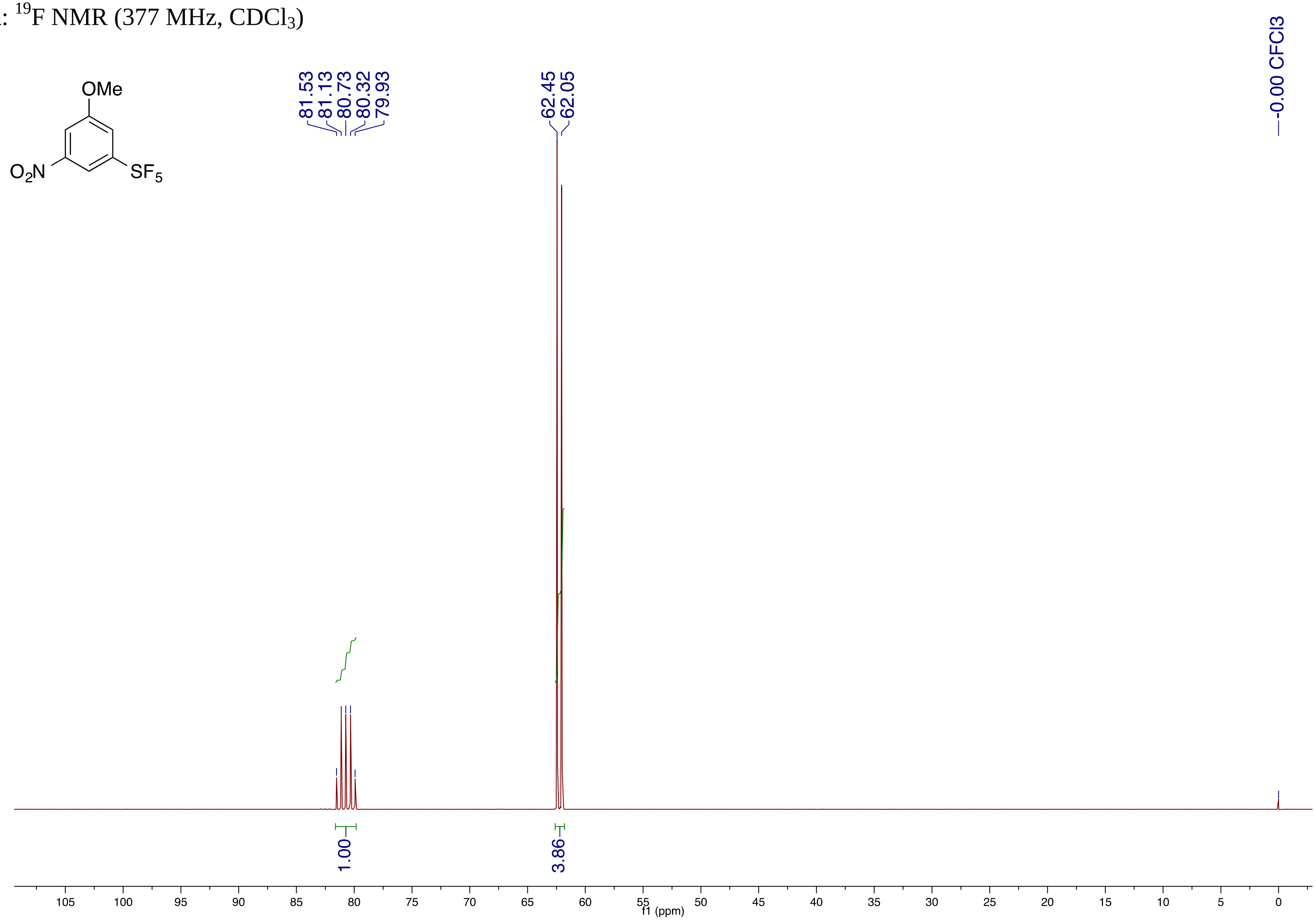

3b: ^{1}H NMR (400 MHz, $CDCl_3$)

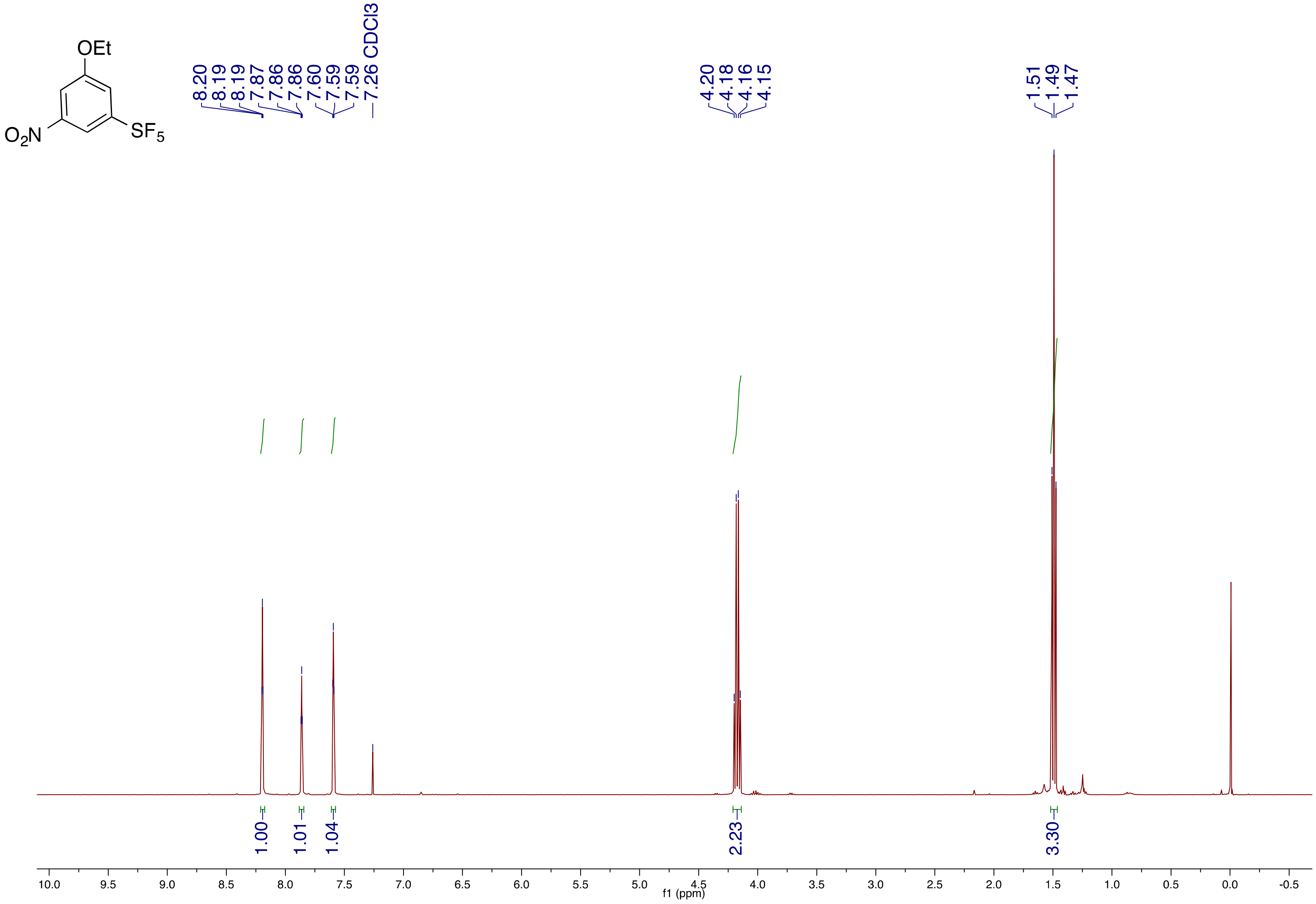

3b: ^{13}C NMR (101 MHz, $CDCl_3$)

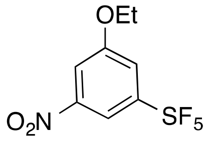

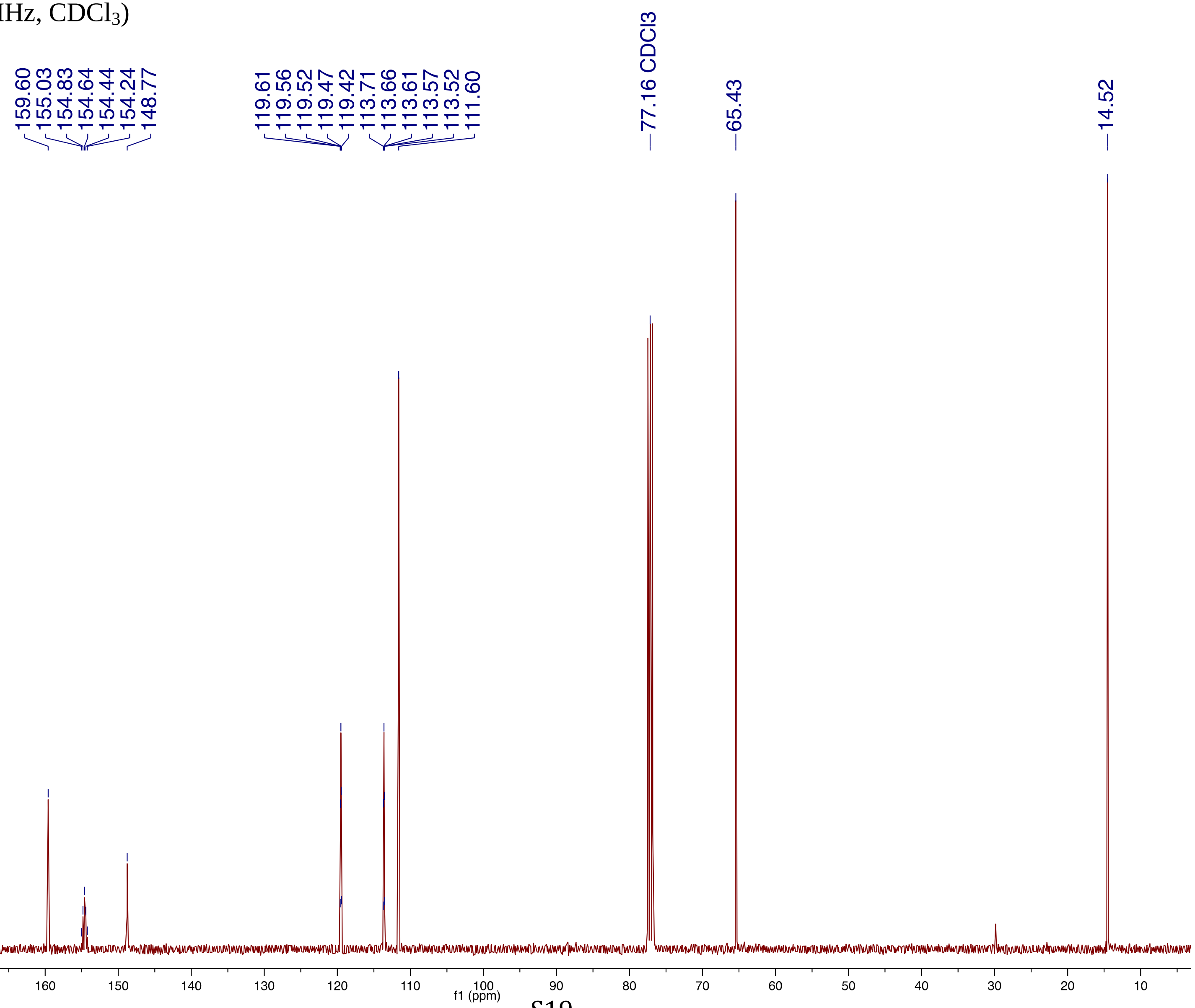

3b: ^{19}F NMR (377 MHz, $CDCl_3$)

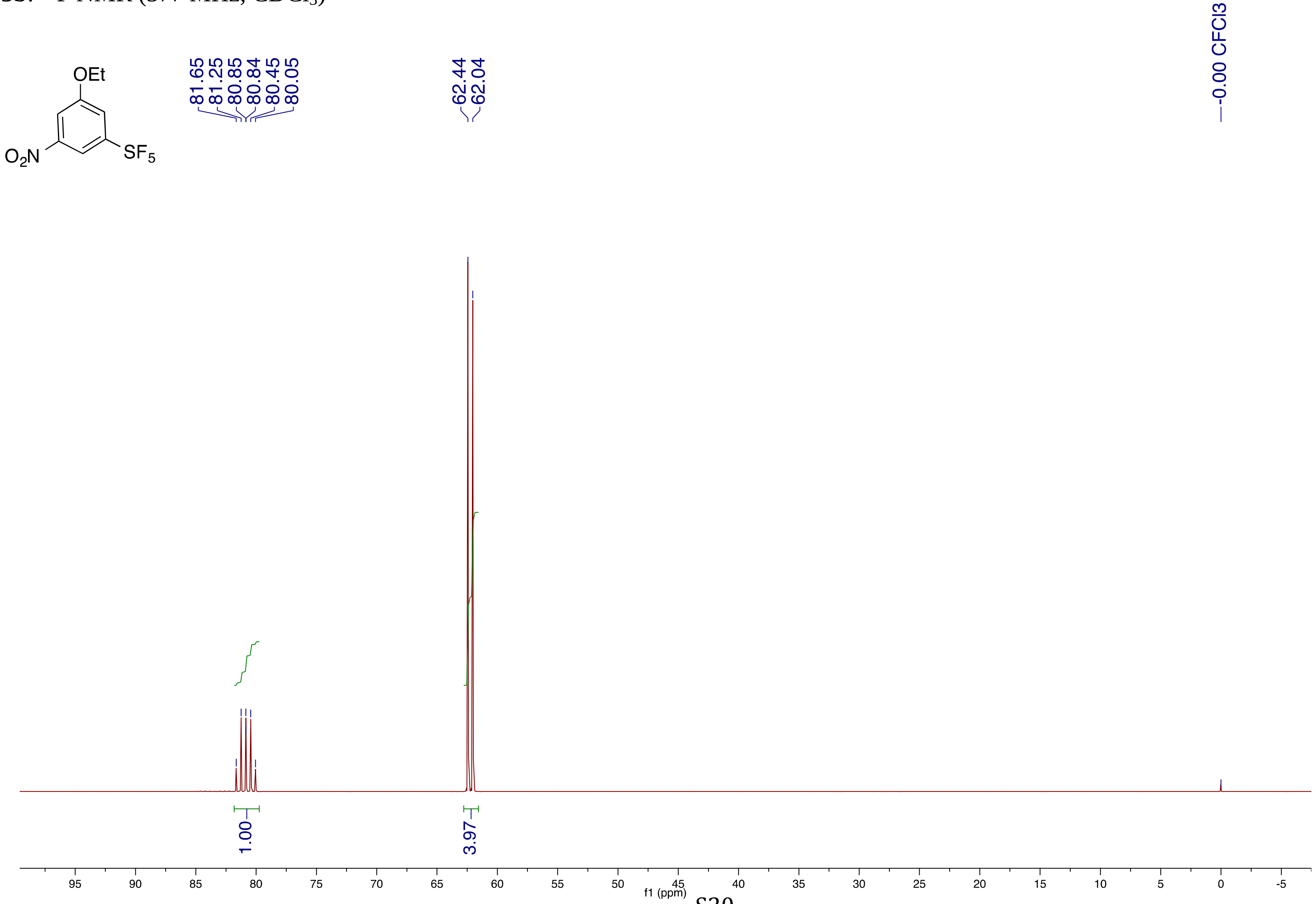

3c: ^{1}H NMR (400 MHz, $CDCl_3$)

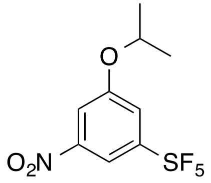

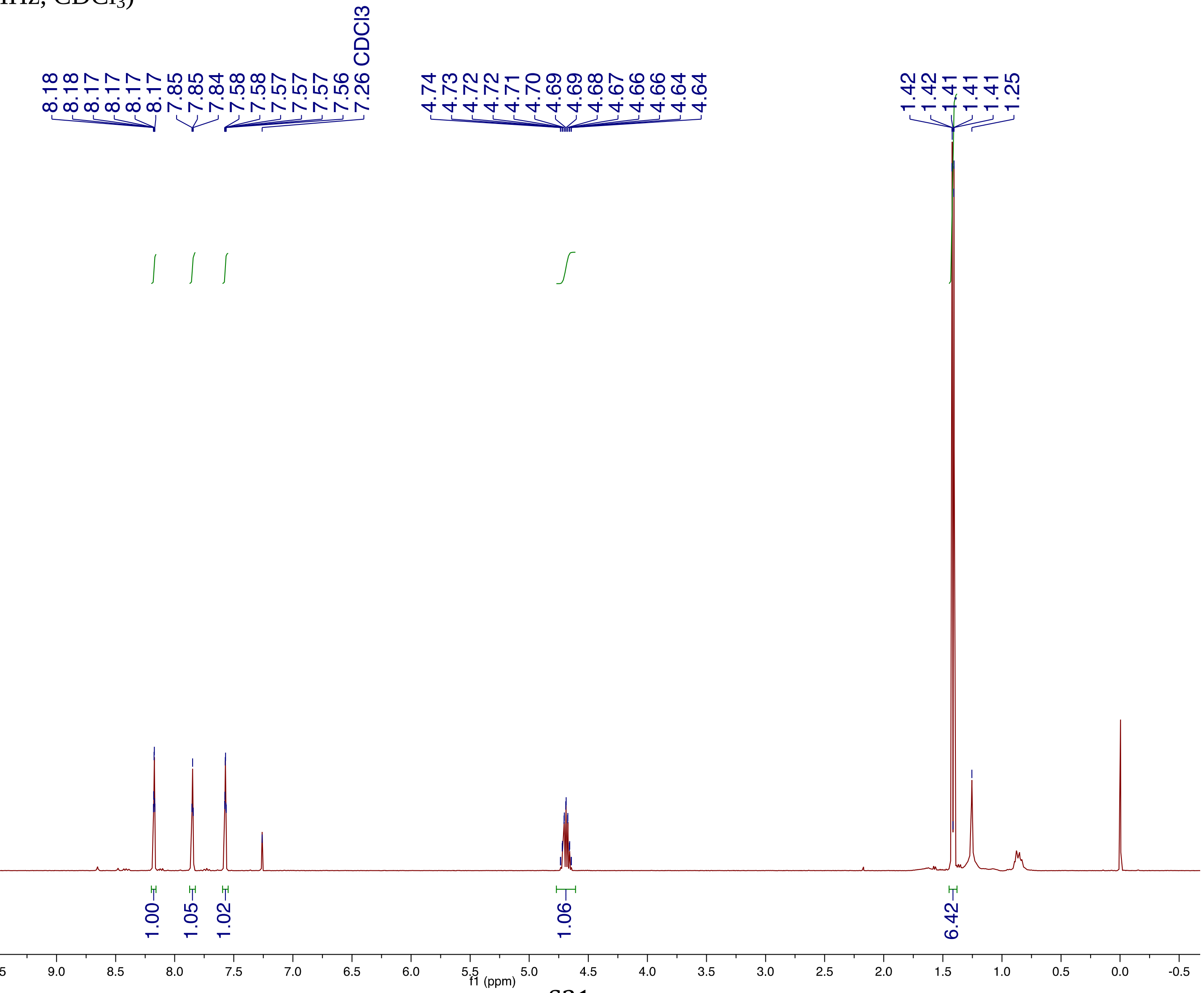

3c: ^{13}C NMR (101 MHz, $CDCl_3$)

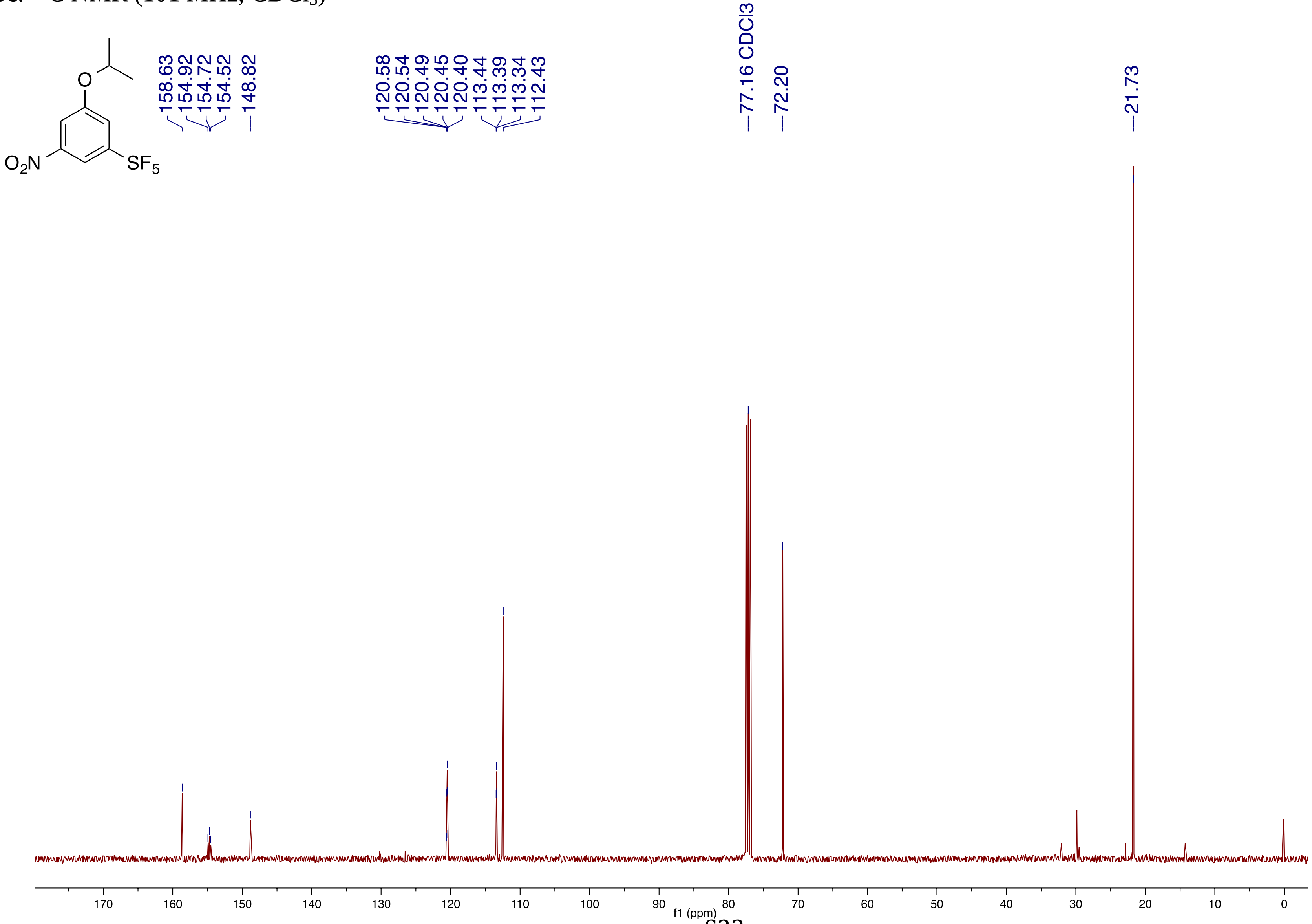

3c: ^{19}F NMR (377 MHz, $CDCl_3$)

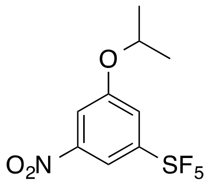

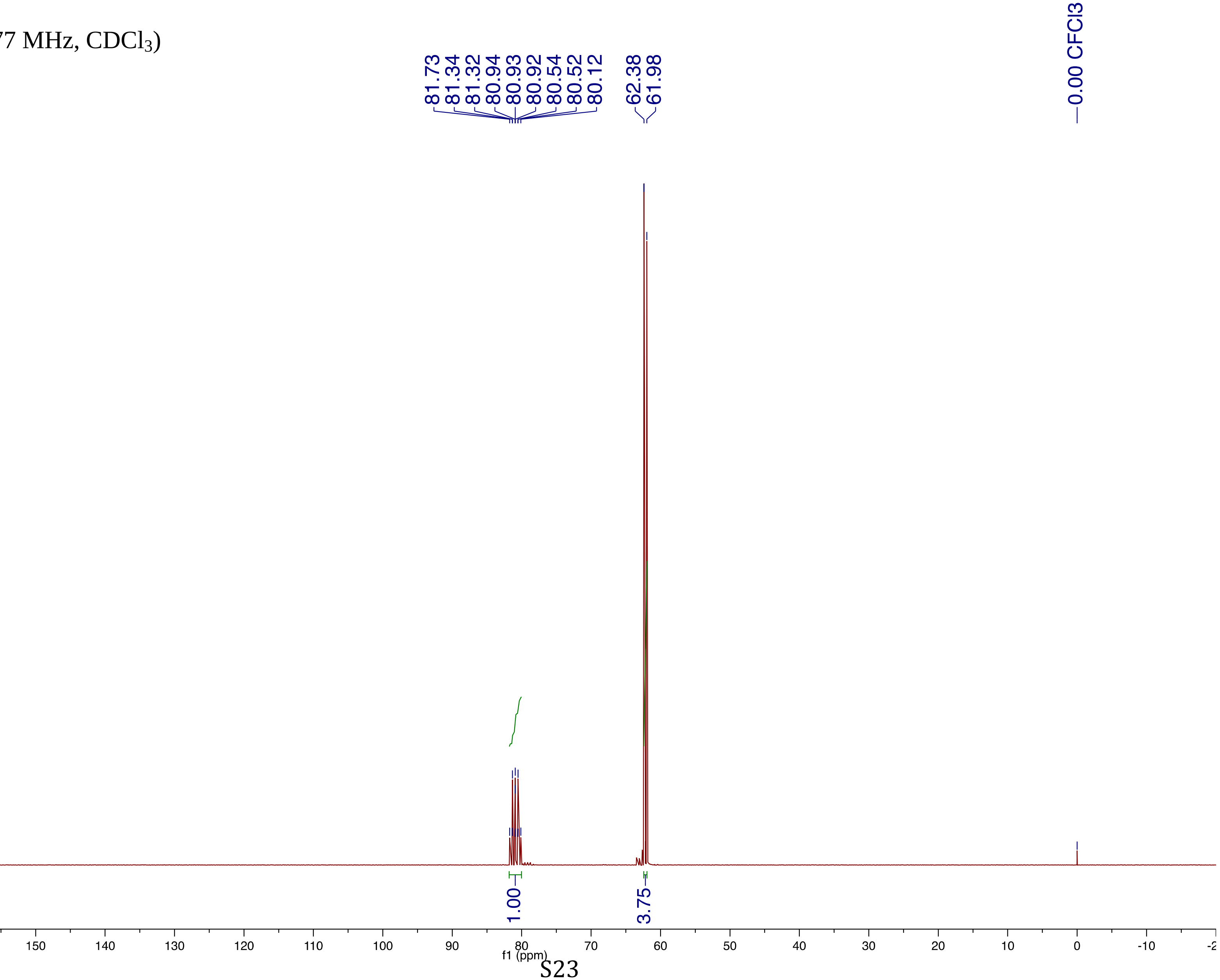

3d: ^{1}H NMR (400 MHz, $CDCl_3$)

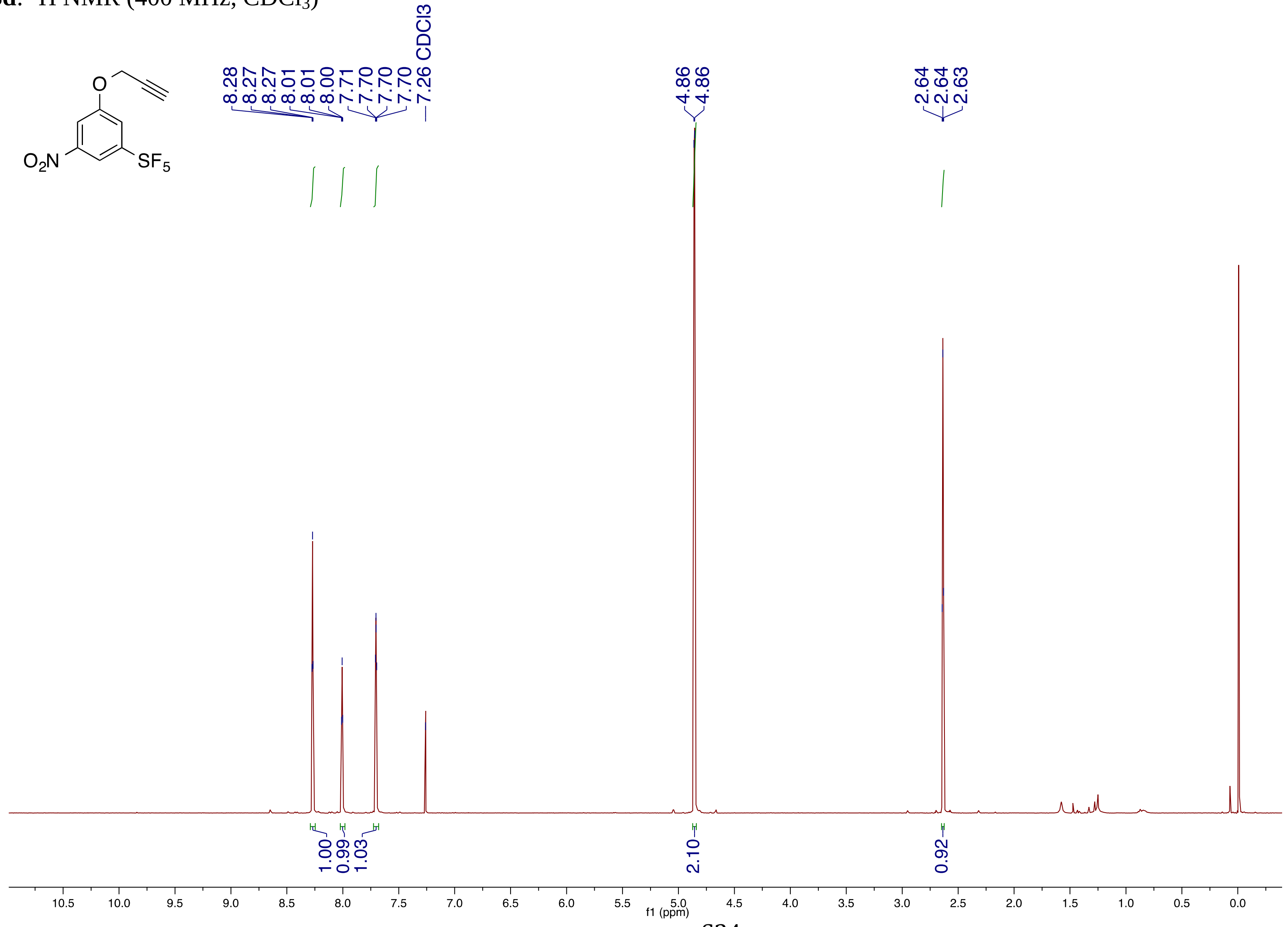

3d: ^{13}C NMR (101 MHz, $CDCl_3$)

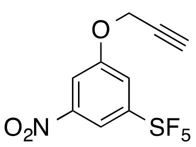

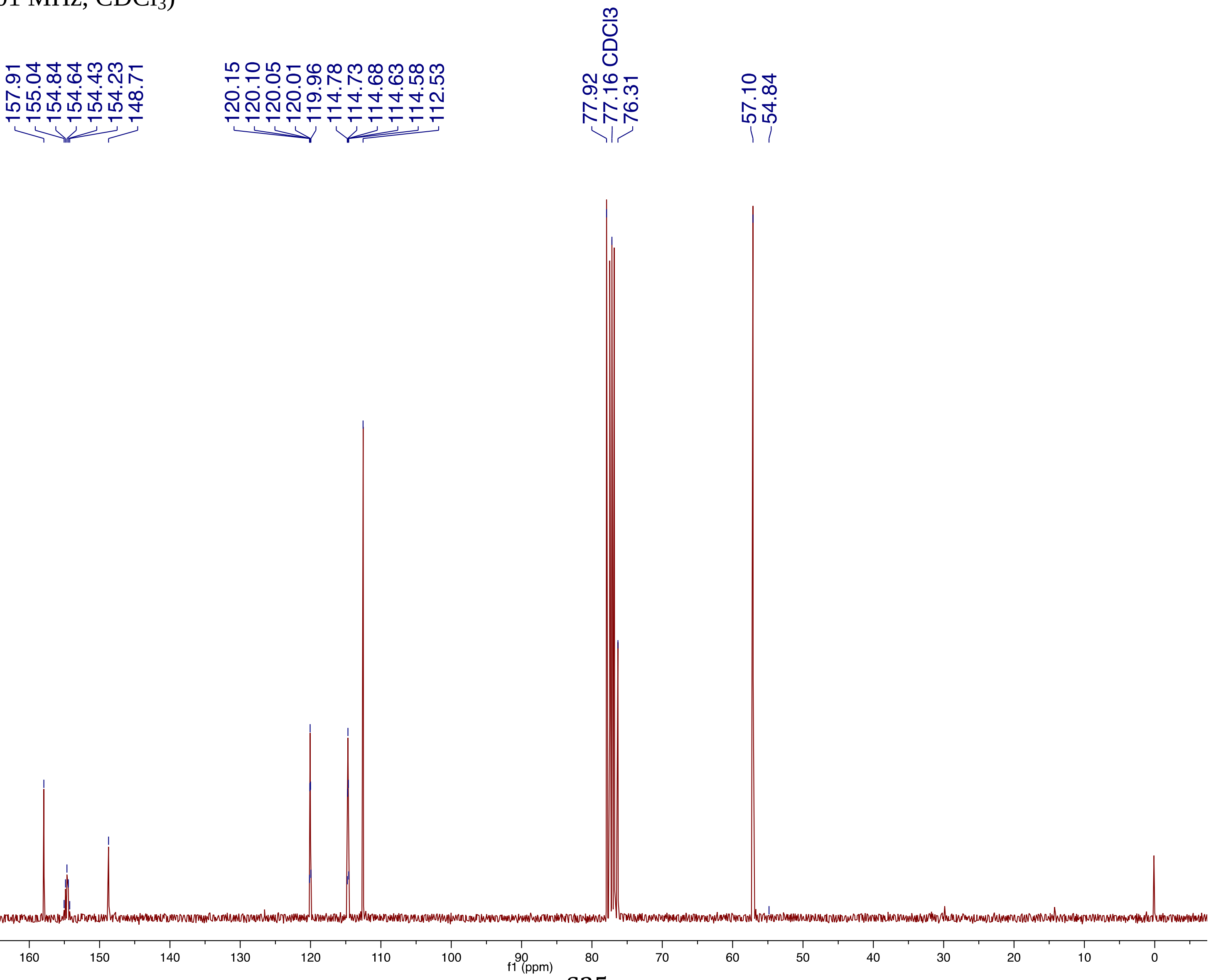

3d: ^{19}F NMR (377 MHz, $CDCl_3$)

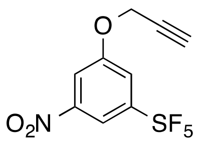

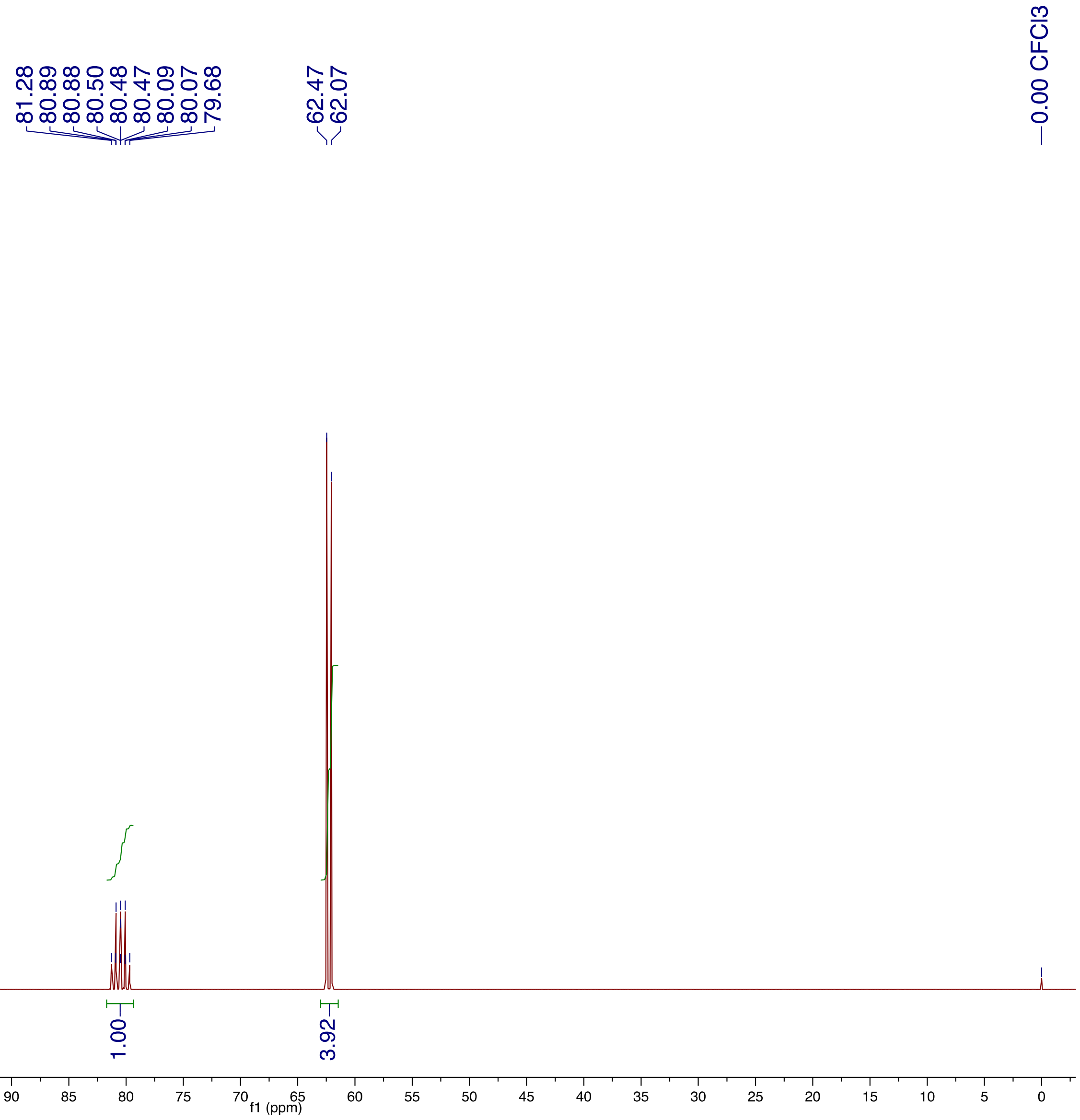

3e: ^{1}H NMR (400 MHz, $CDCl_3$)

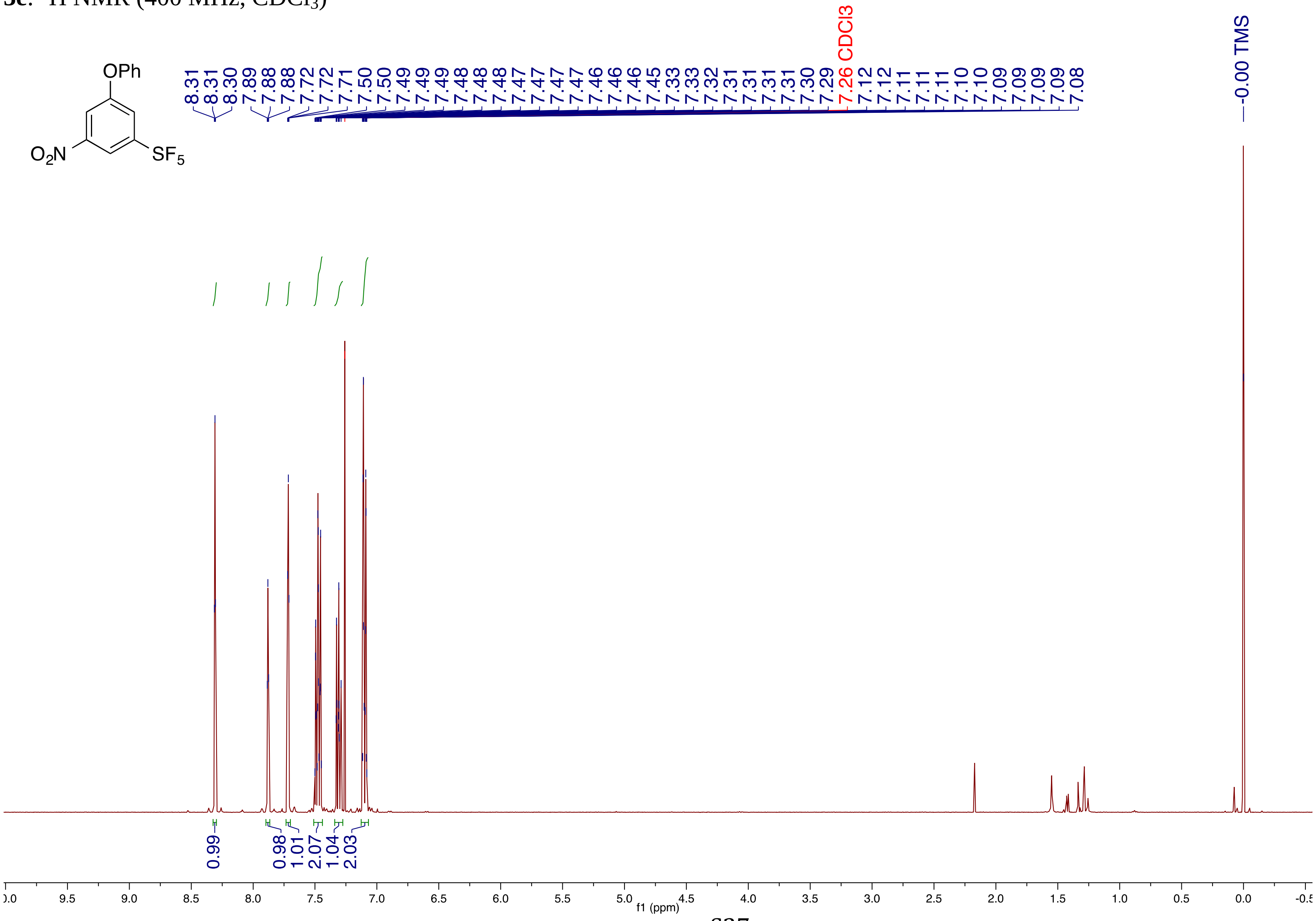

3e: ^{13}C NMR (101 MHz, $CDCl_3$)

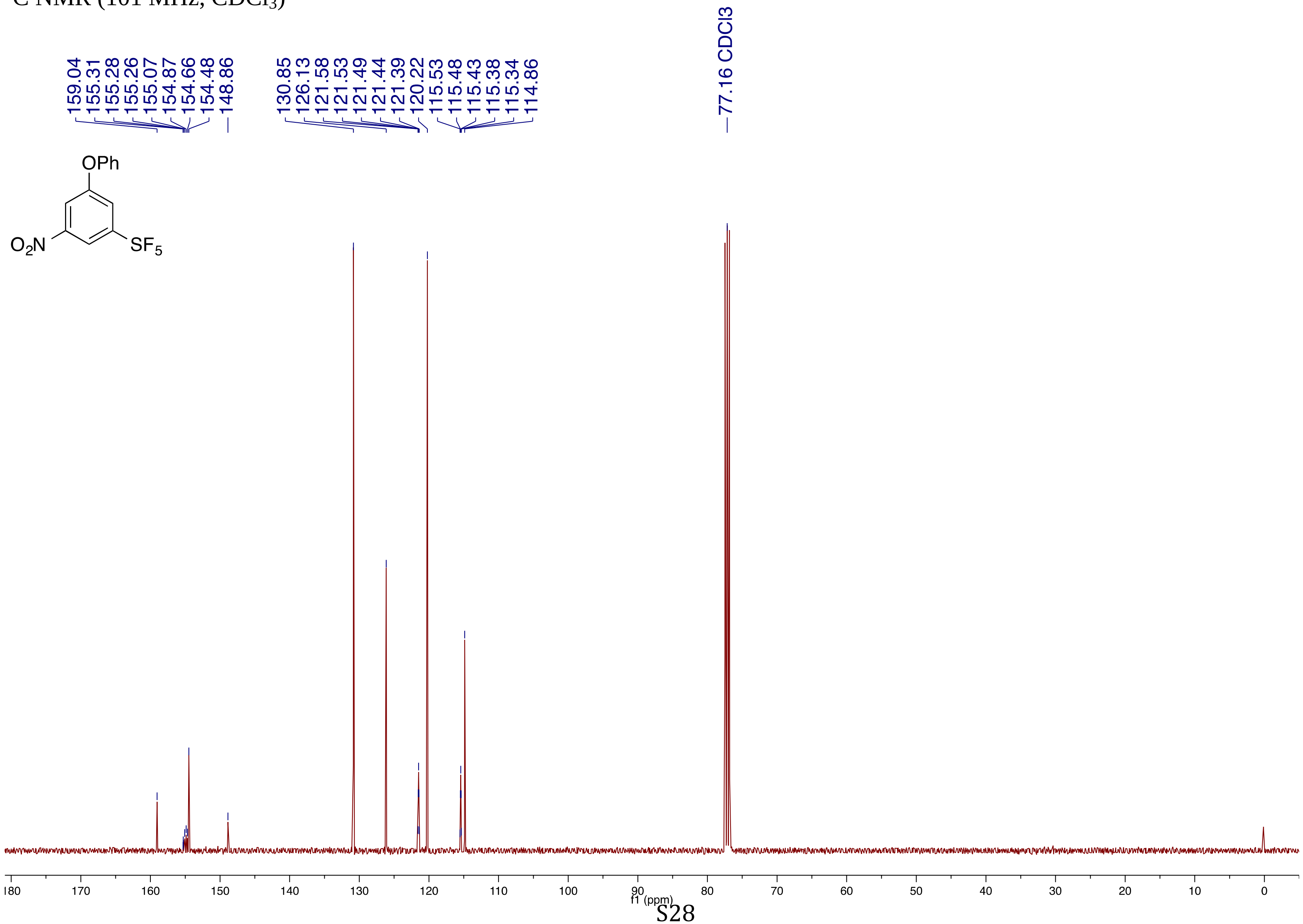

3e: ^{19}F NMR (377 MHz, $CDCl_3$)

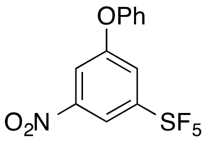

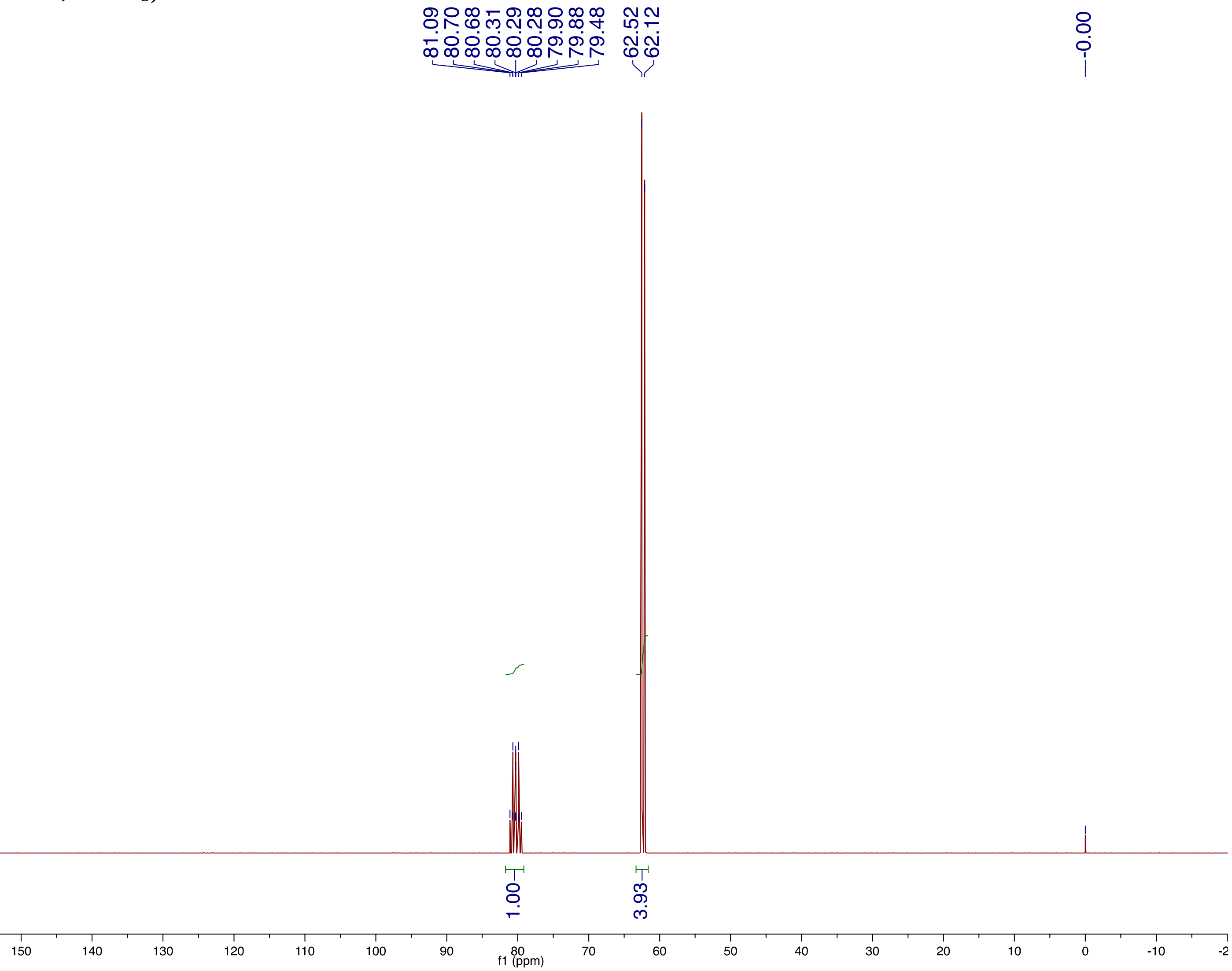

3f: ^{1}H NMR (400 MHz, $CDCl_3$)

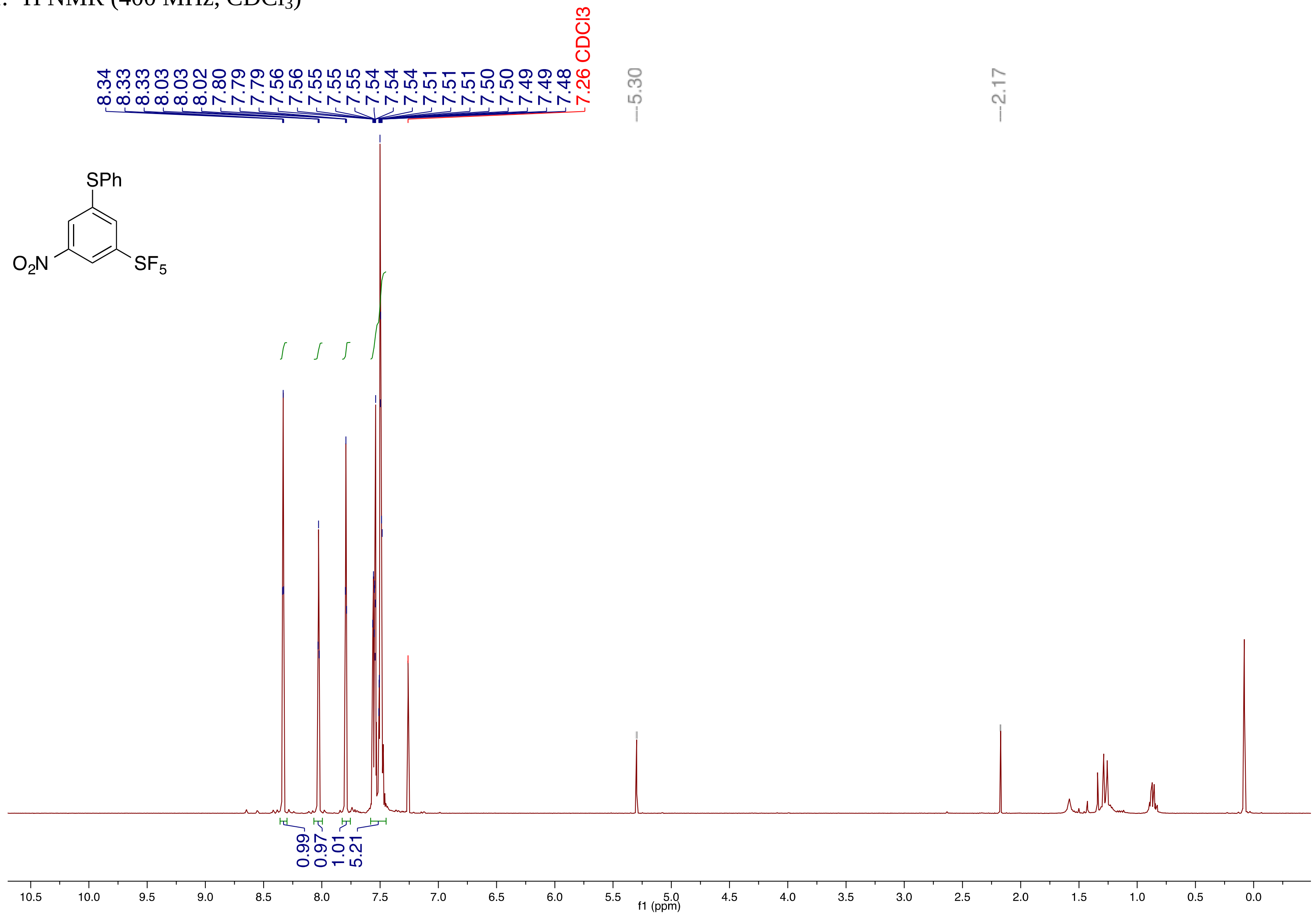

3f: ^{13}C NMR (101 MHz, $CDCl_3$)

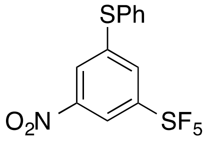

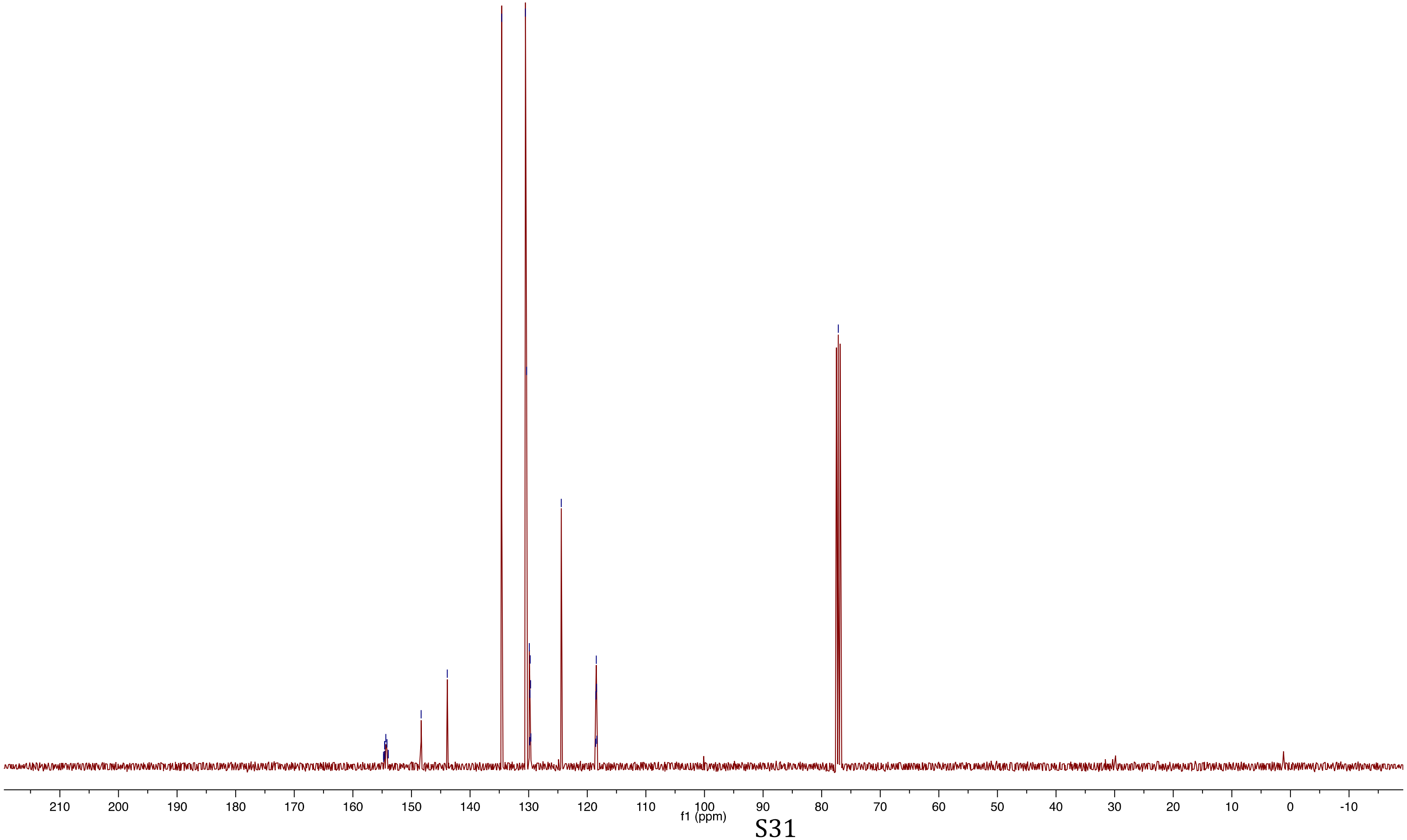

3f: ^{19}F NMR (377 MHz, $CDCl_3$)

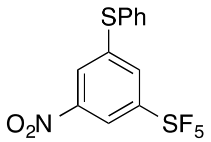

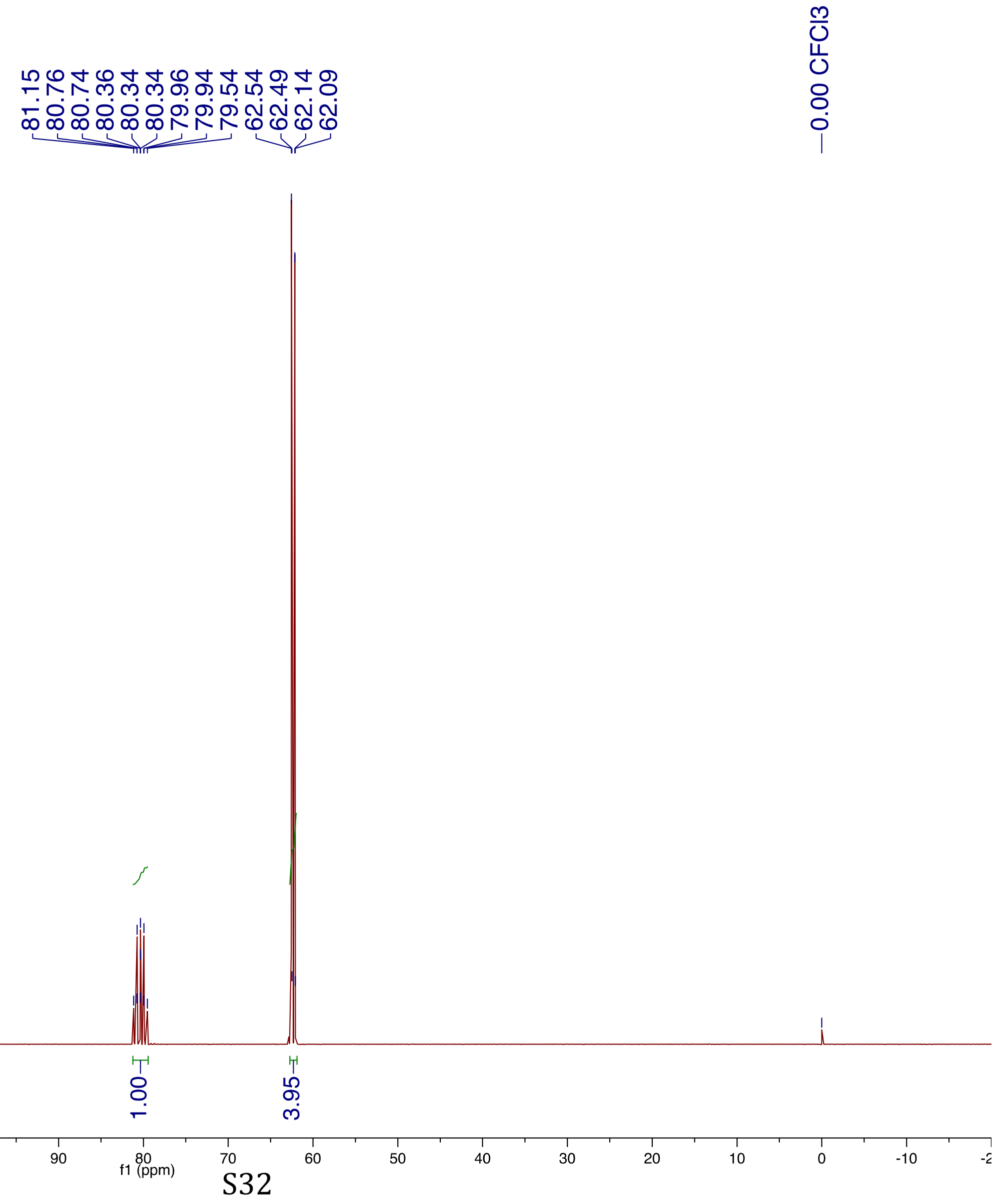

3g: ^{1}H NMR (400 MHz, $CDCl_3$)

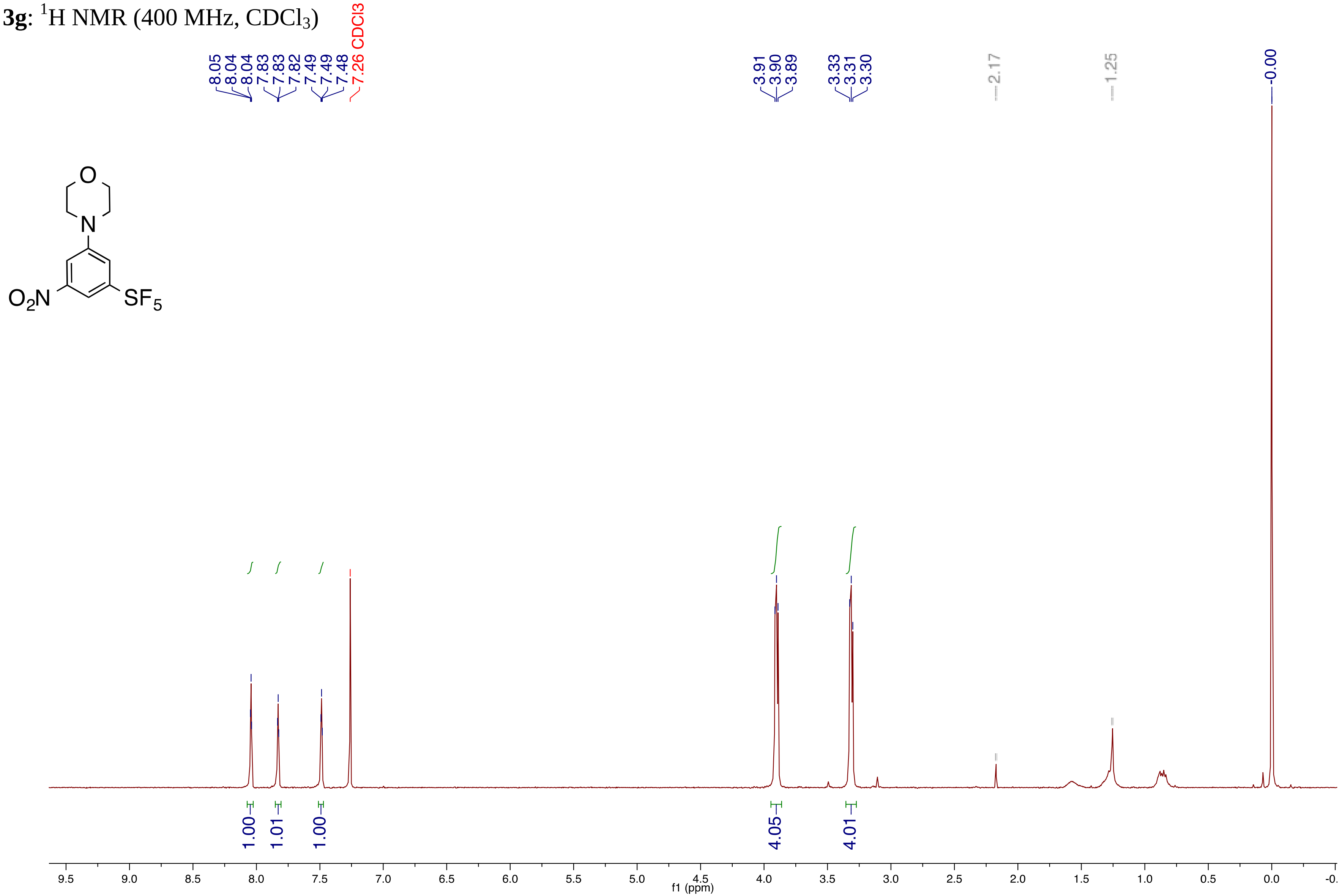

3f: ^{13}C NMR (101 MHz, $CDCl_3$)

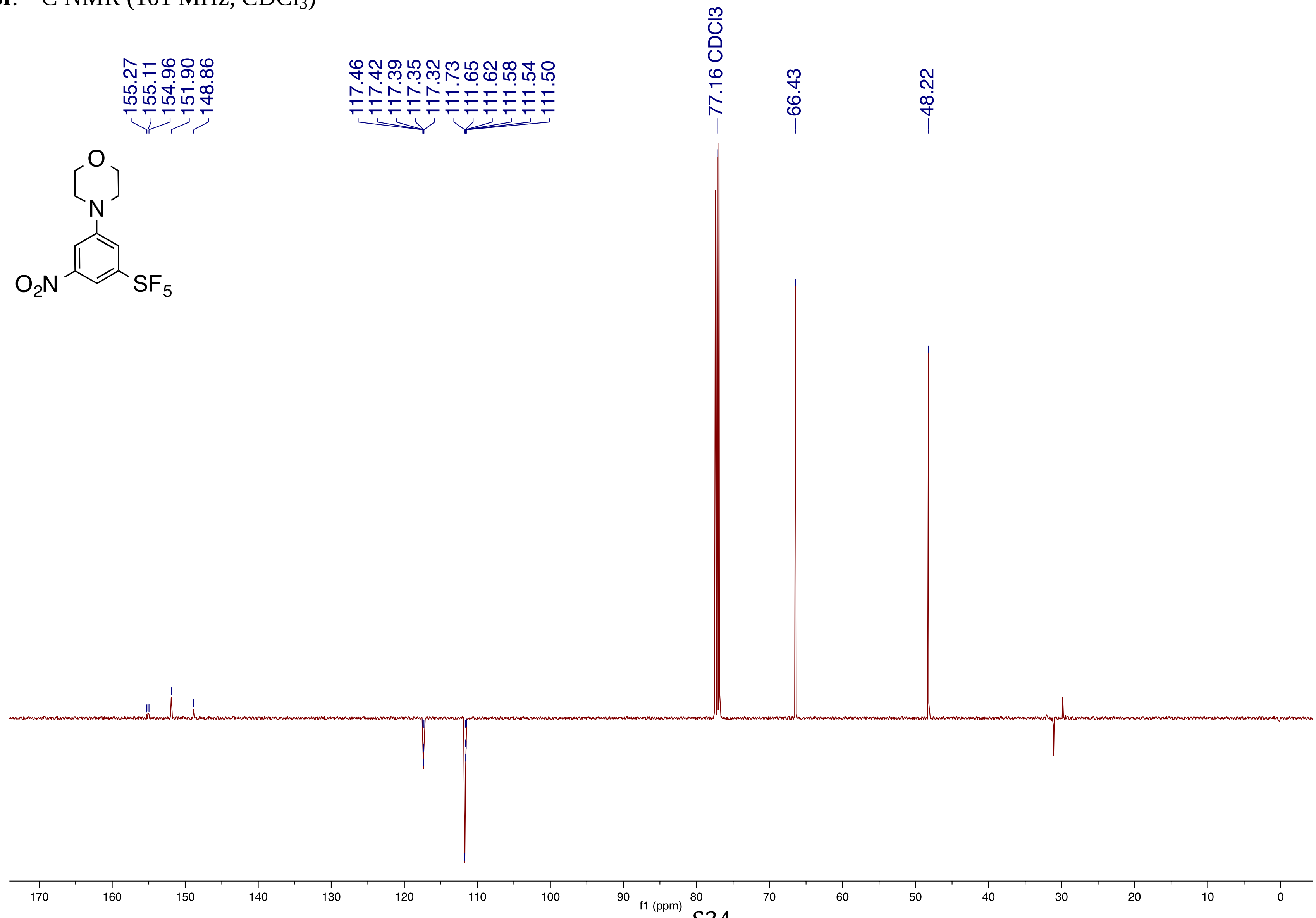

3g: ^{19}F NMR (377 MHz, $CDCl_3$)

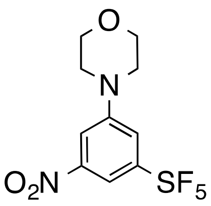

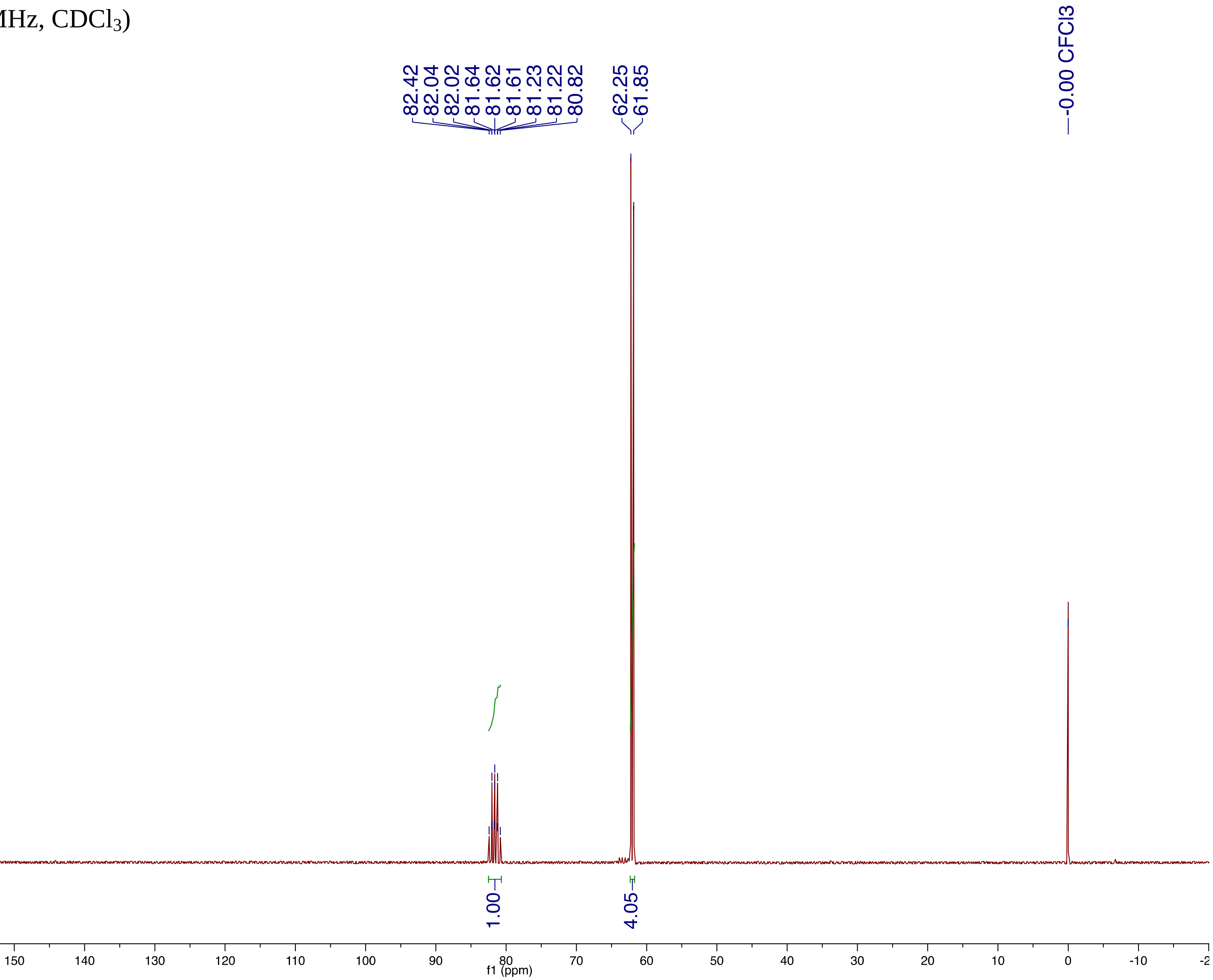

3h: ^{1}H NMR (400 MHz,$CDCl_3$)

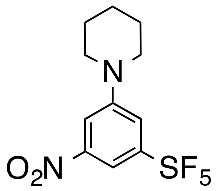

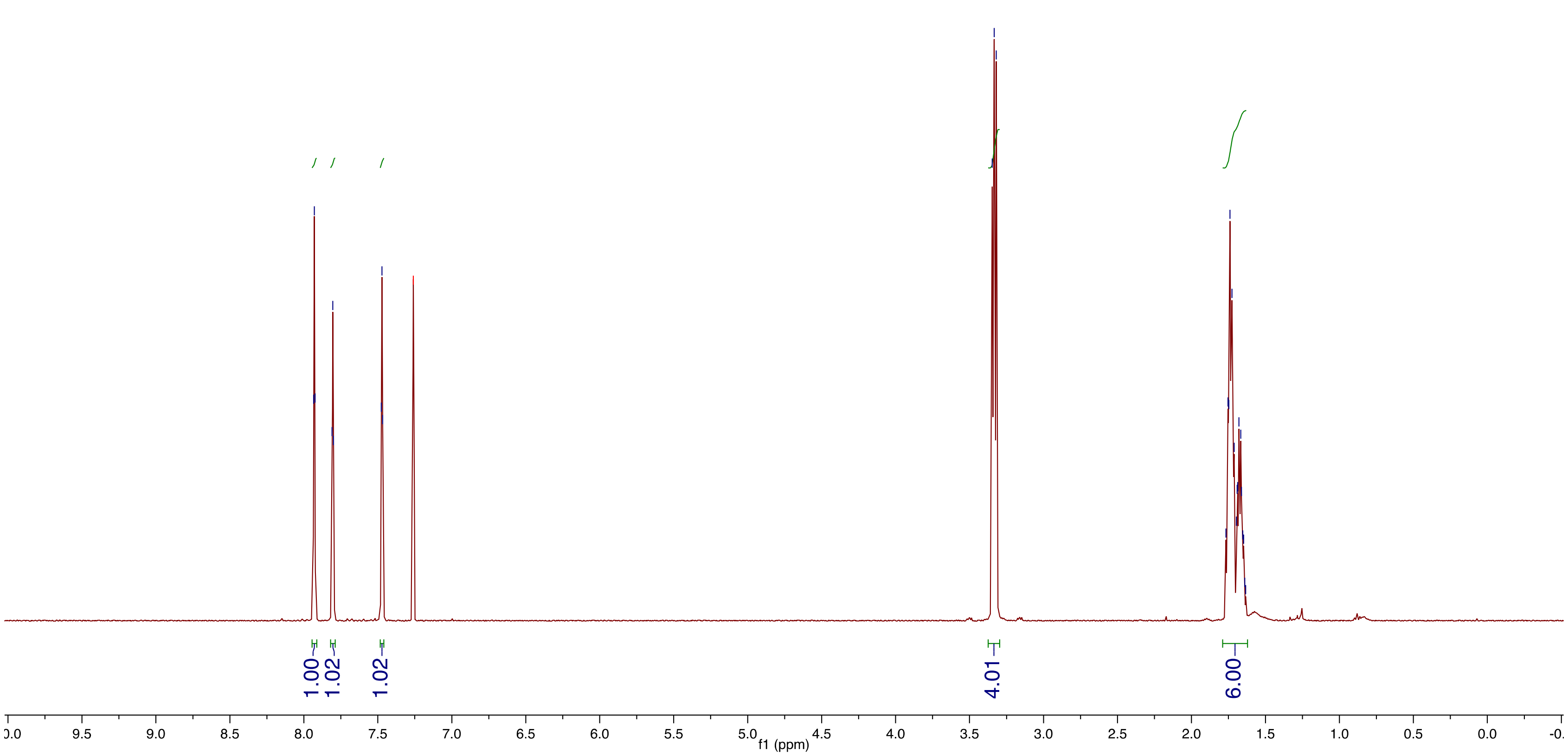

3h: ^{13}C NMR (101 MHz, $CDCl_3$)

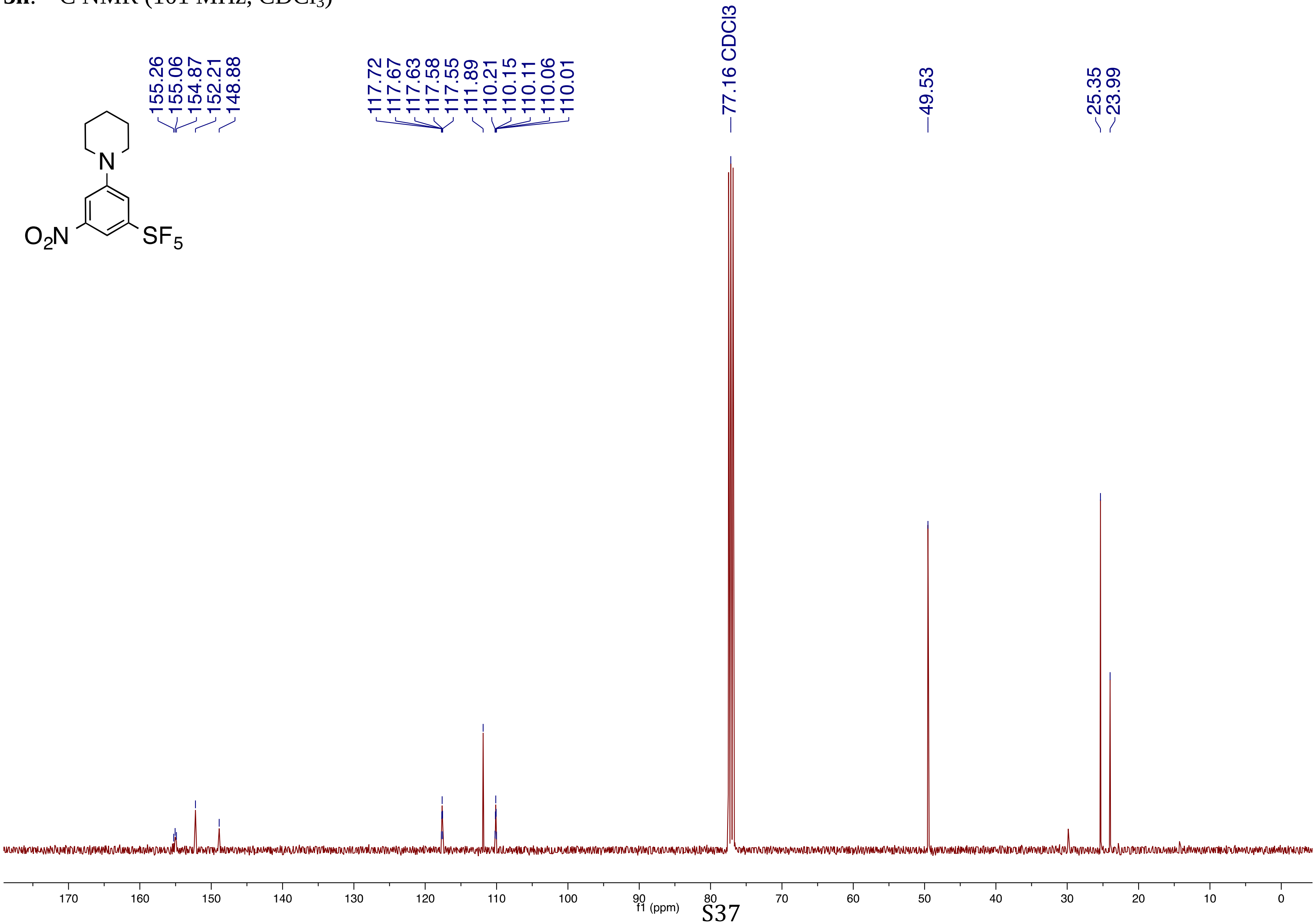

3h: ^{19}F NMR (377 MHz, $CDCl_3$)

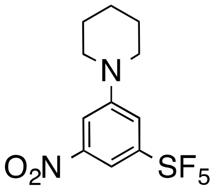

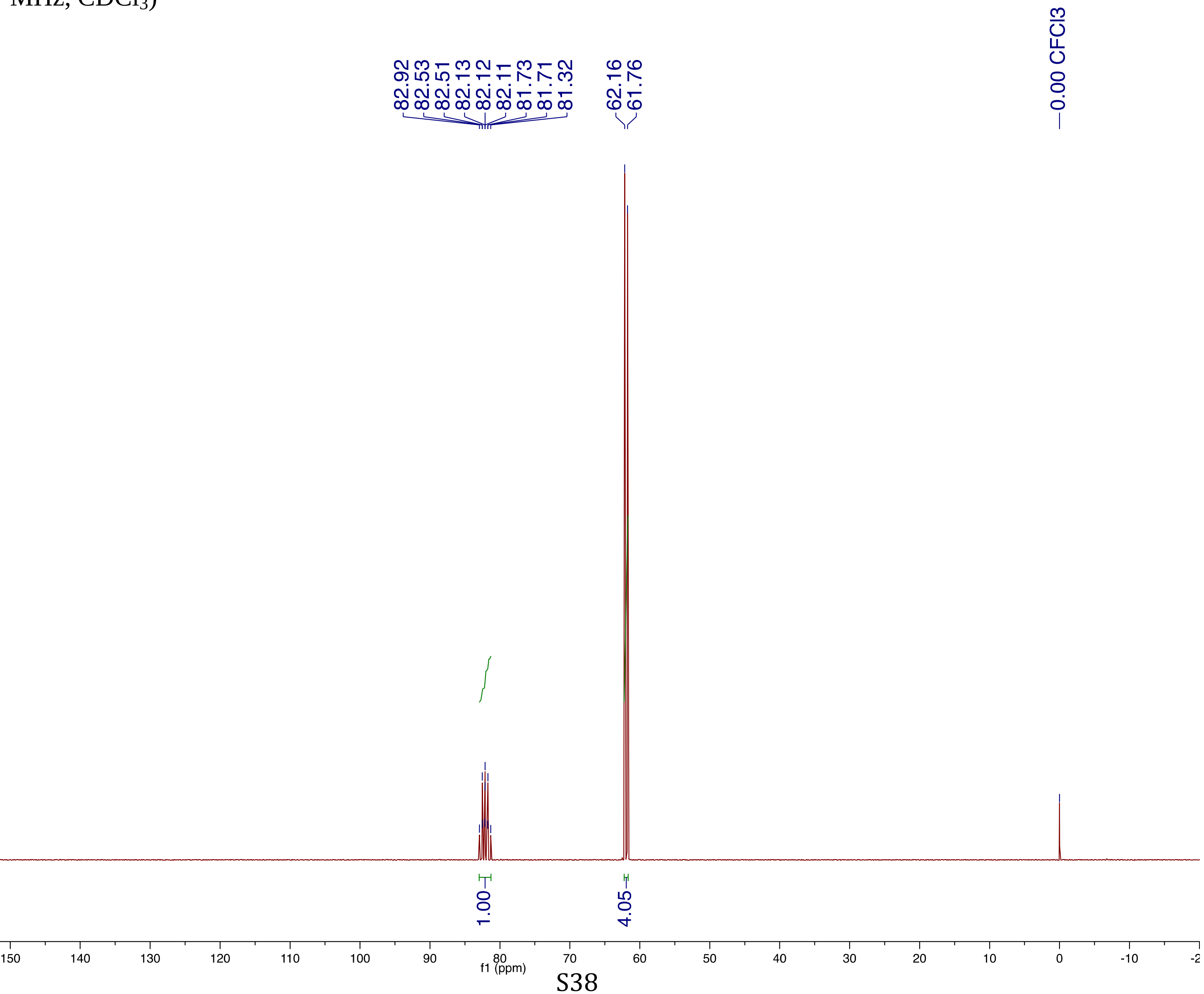

3i: ^{1}H NMR (400 MHz, $CDCl_3$)

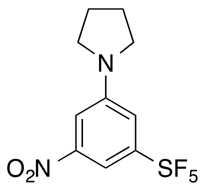

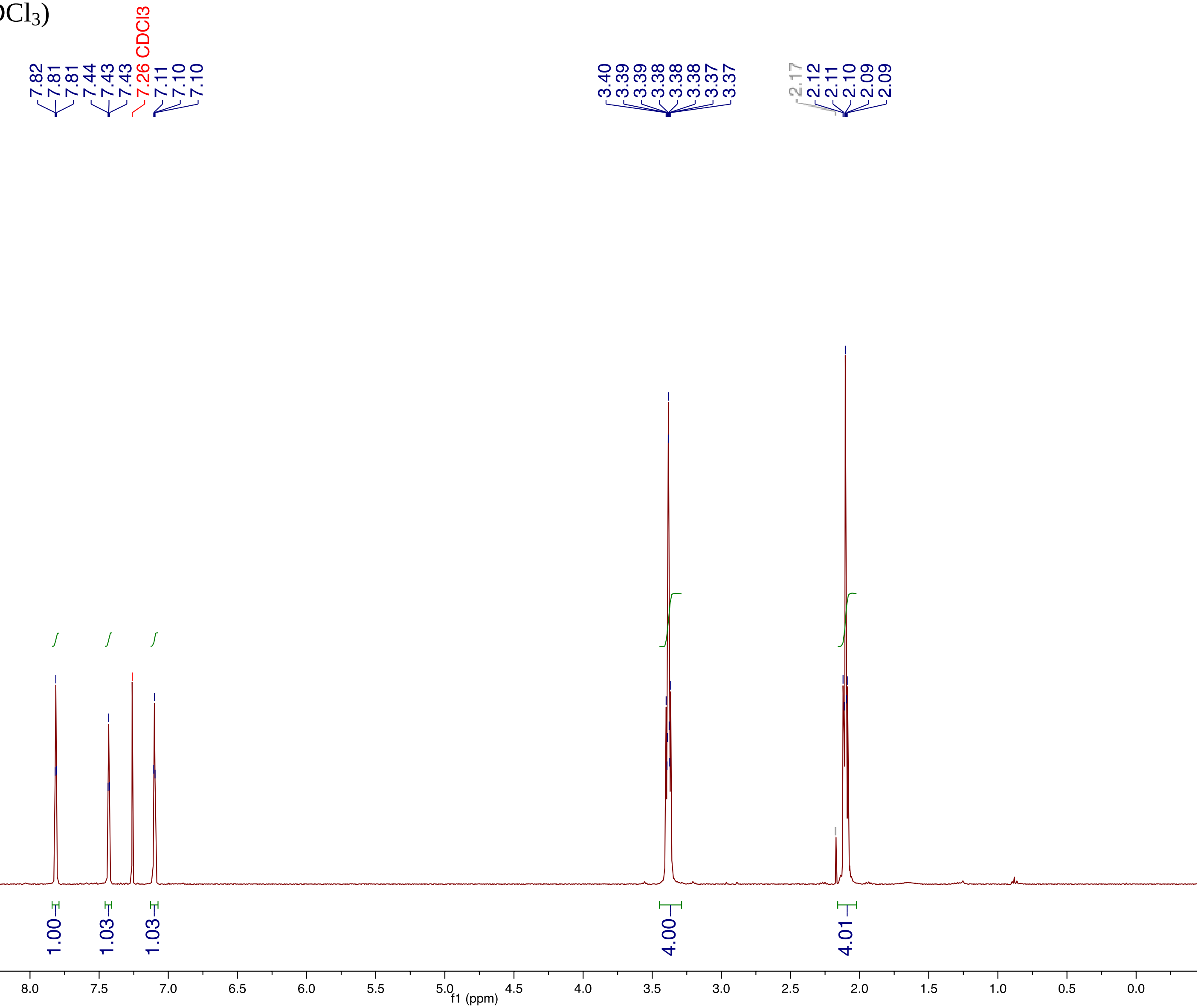

3i: ^{13}C NMR (101 MHz, $CDCl_3$)

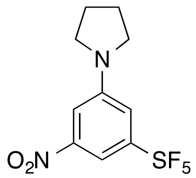

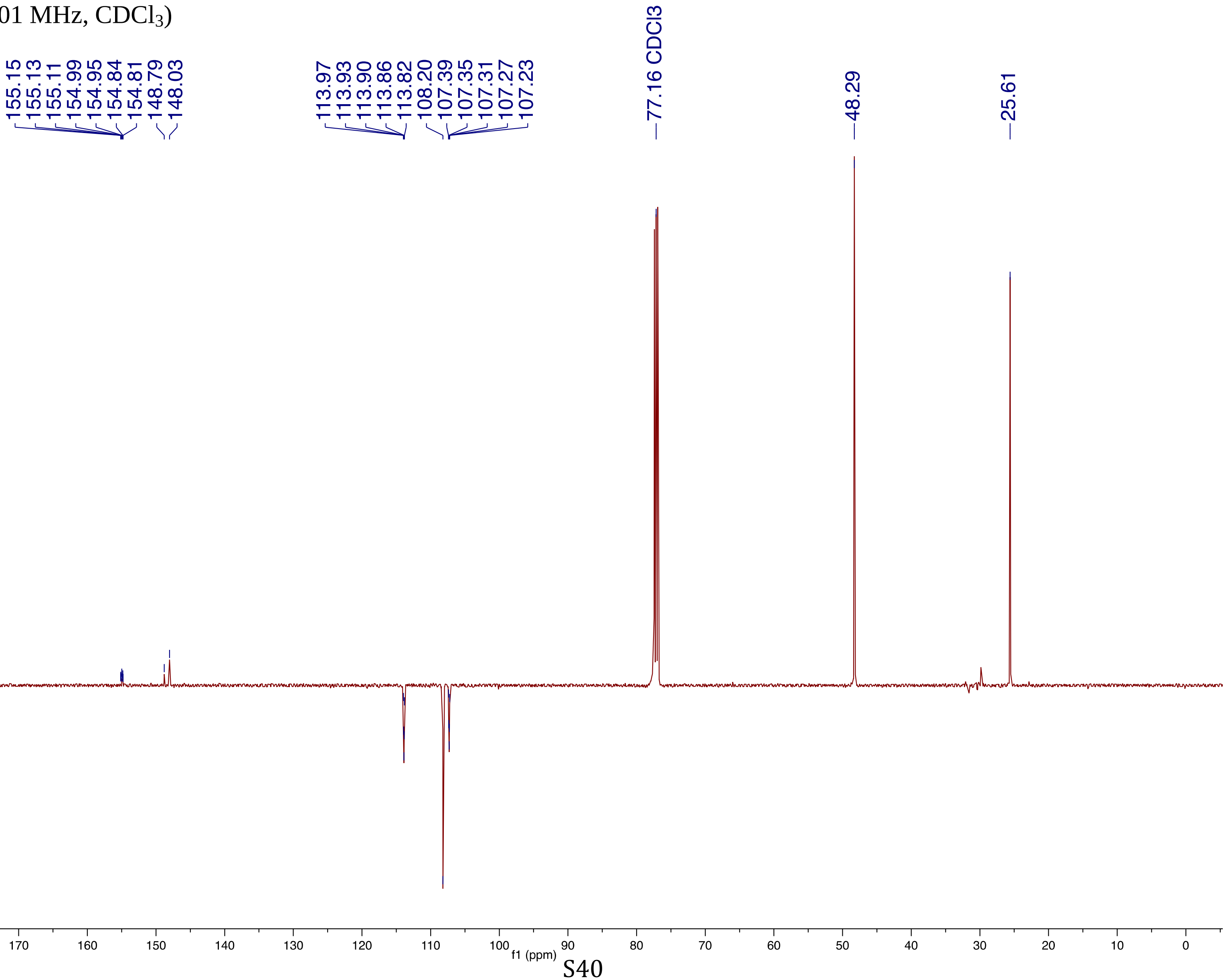

3i: ^{19}F NMR (377 MHz, $CDCl_3$)

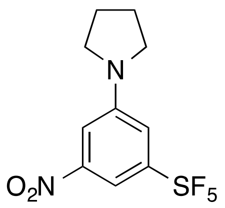

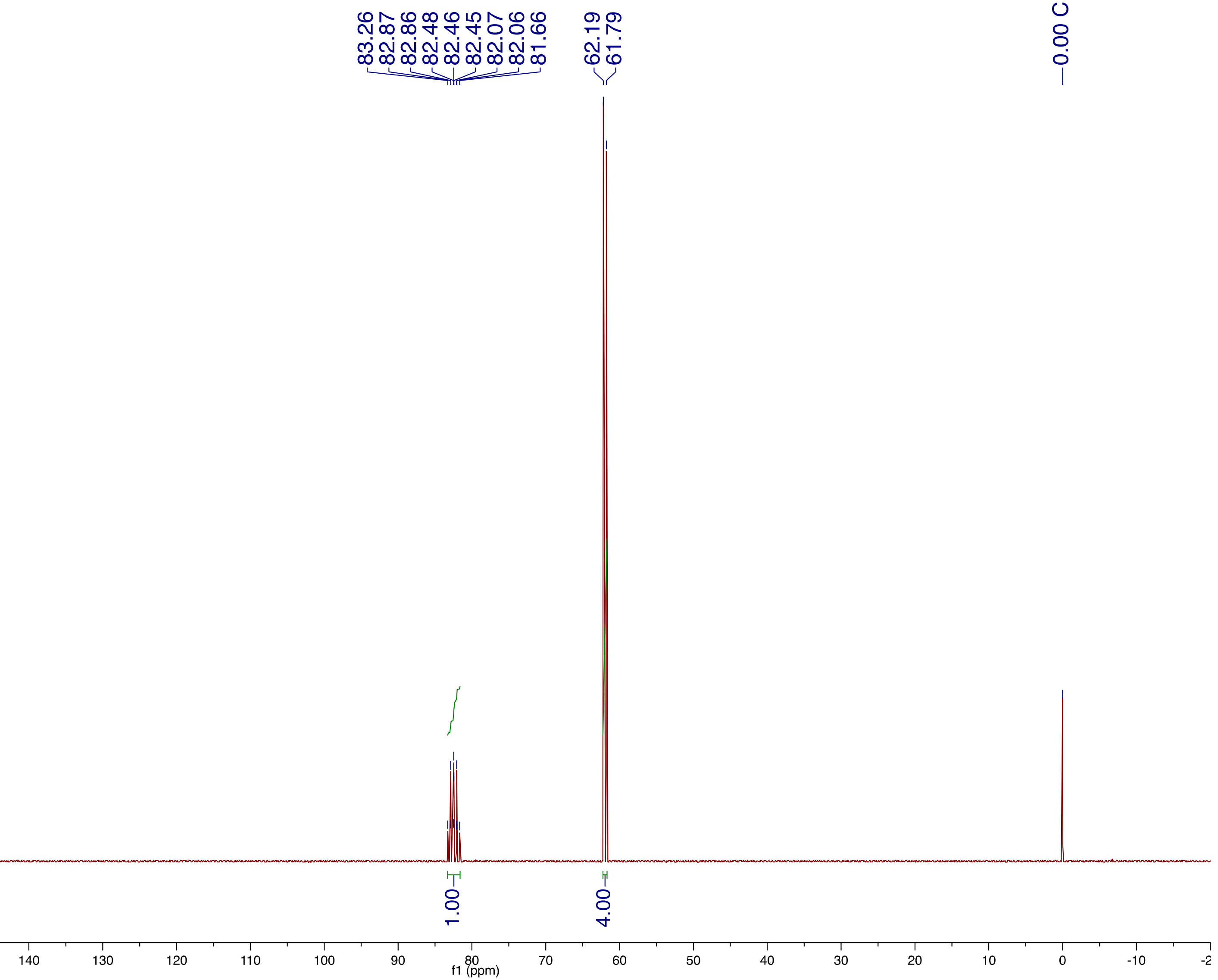

3j: ^{1}H NMR (400 MHz, $CDCl_3$)

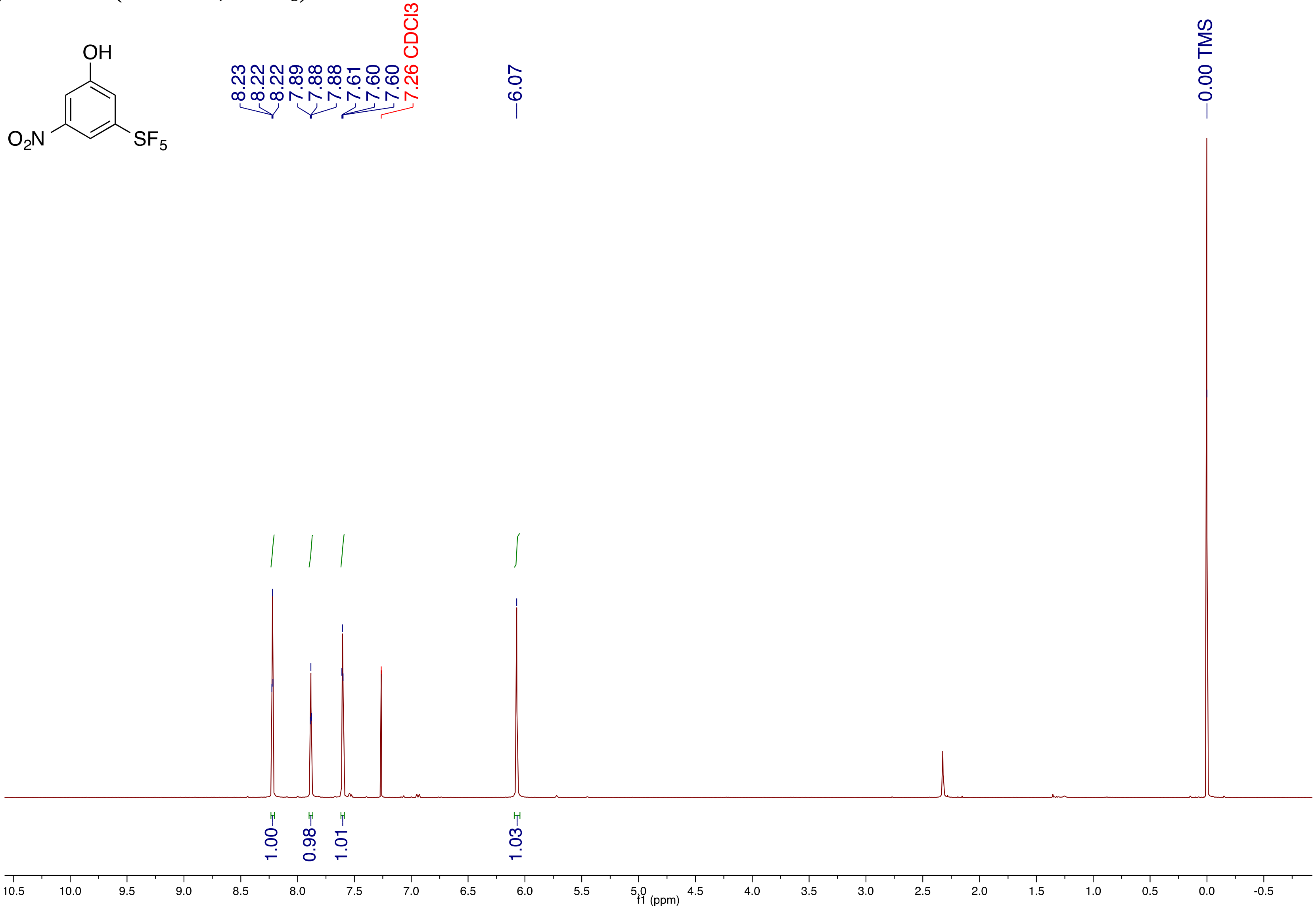

3j: ^{13}C NMR (101 MHz, $CDCl_3$)

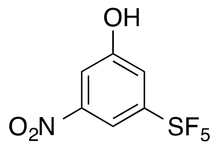

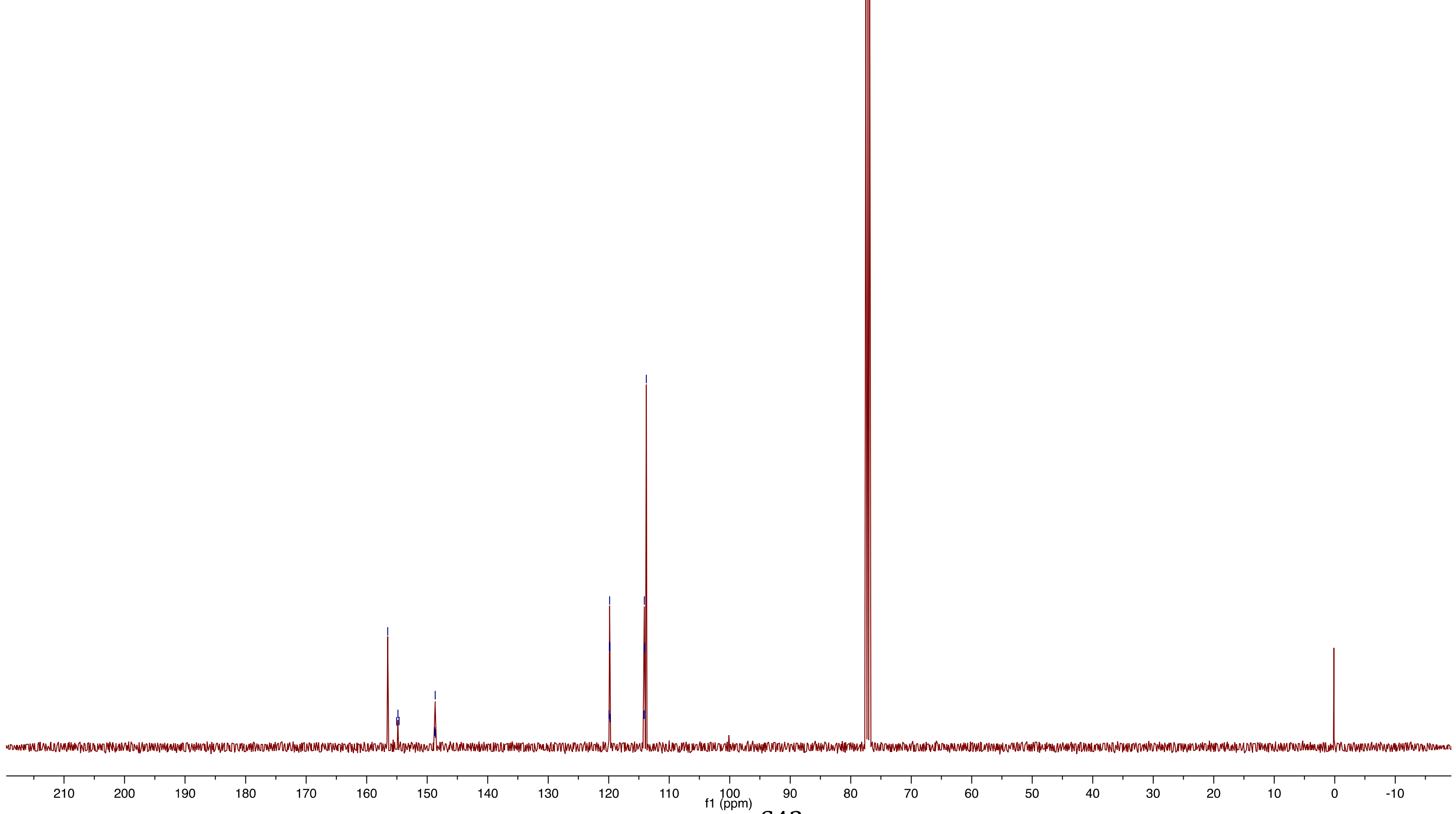

3j: ^{19}F NMR (377 MHz, $CDCl_3$)

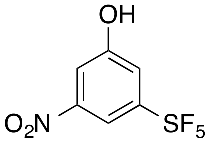

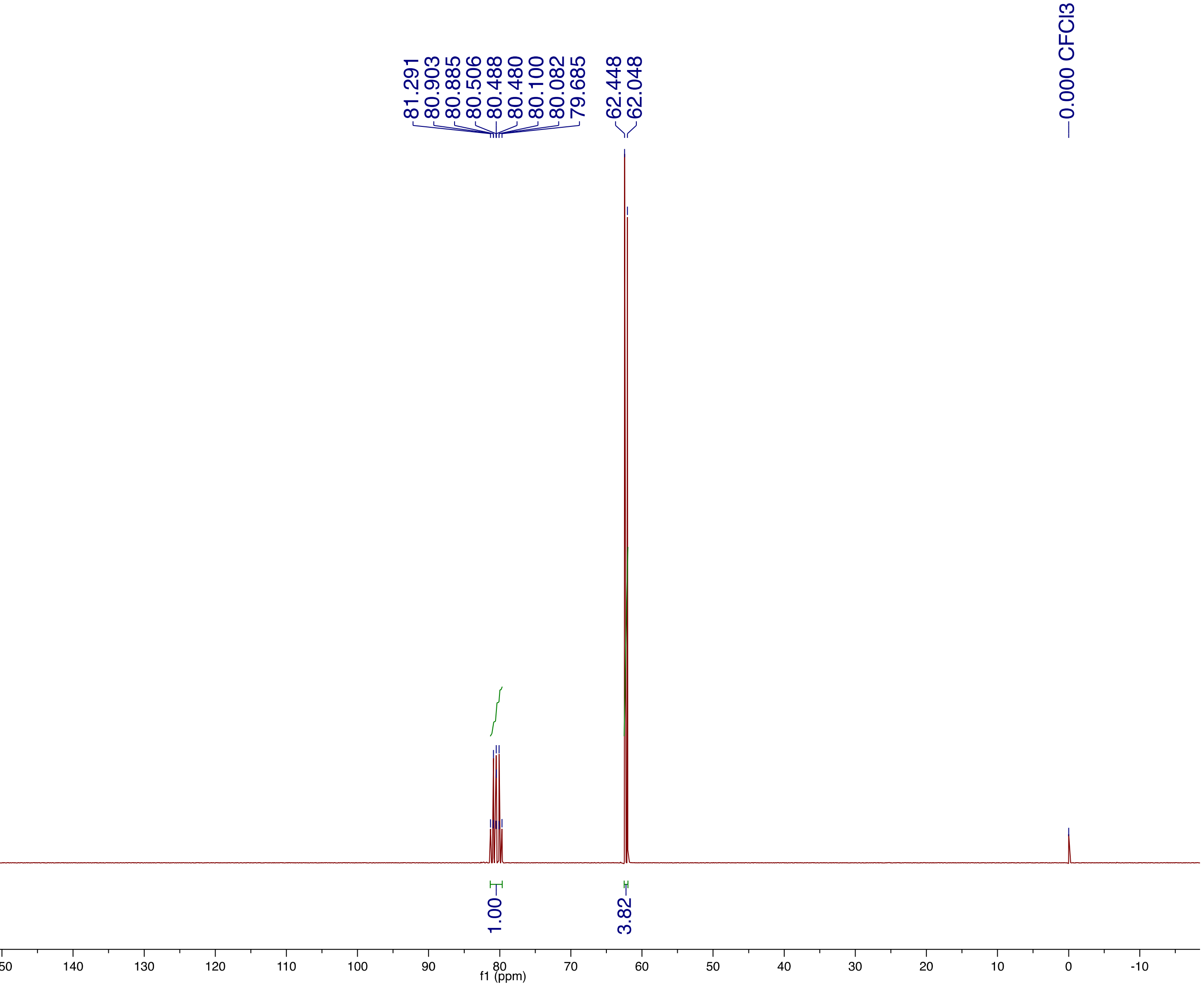

3k: ^{1}H NMR (500 MHz, acetone-d_6)

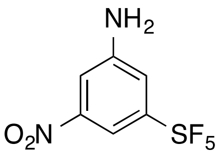

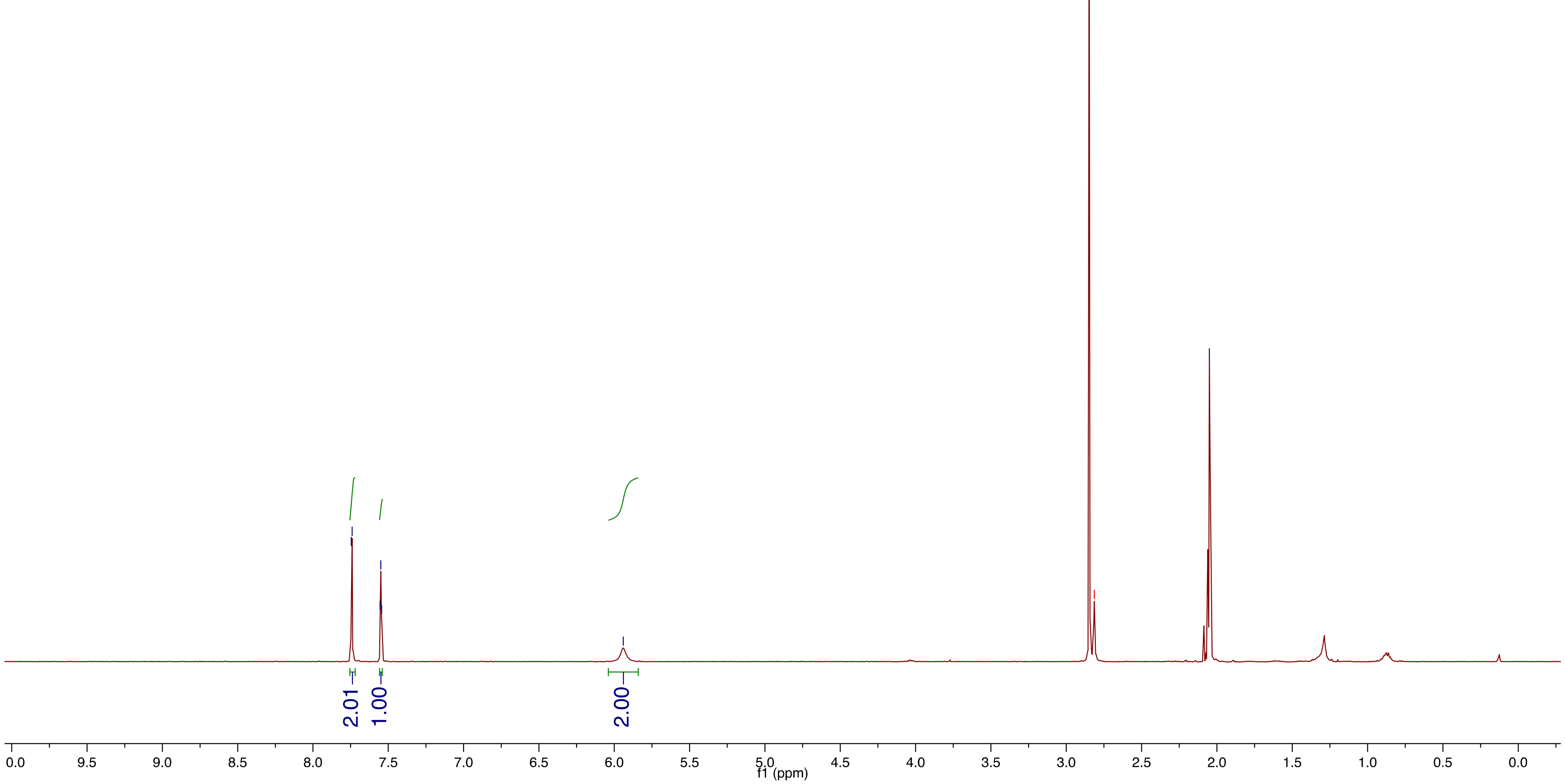

3k: ^{13}C NMR (126 MHz, acetone-d_6)

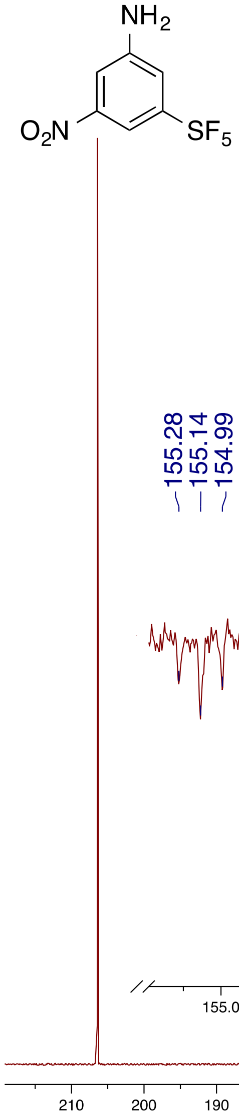

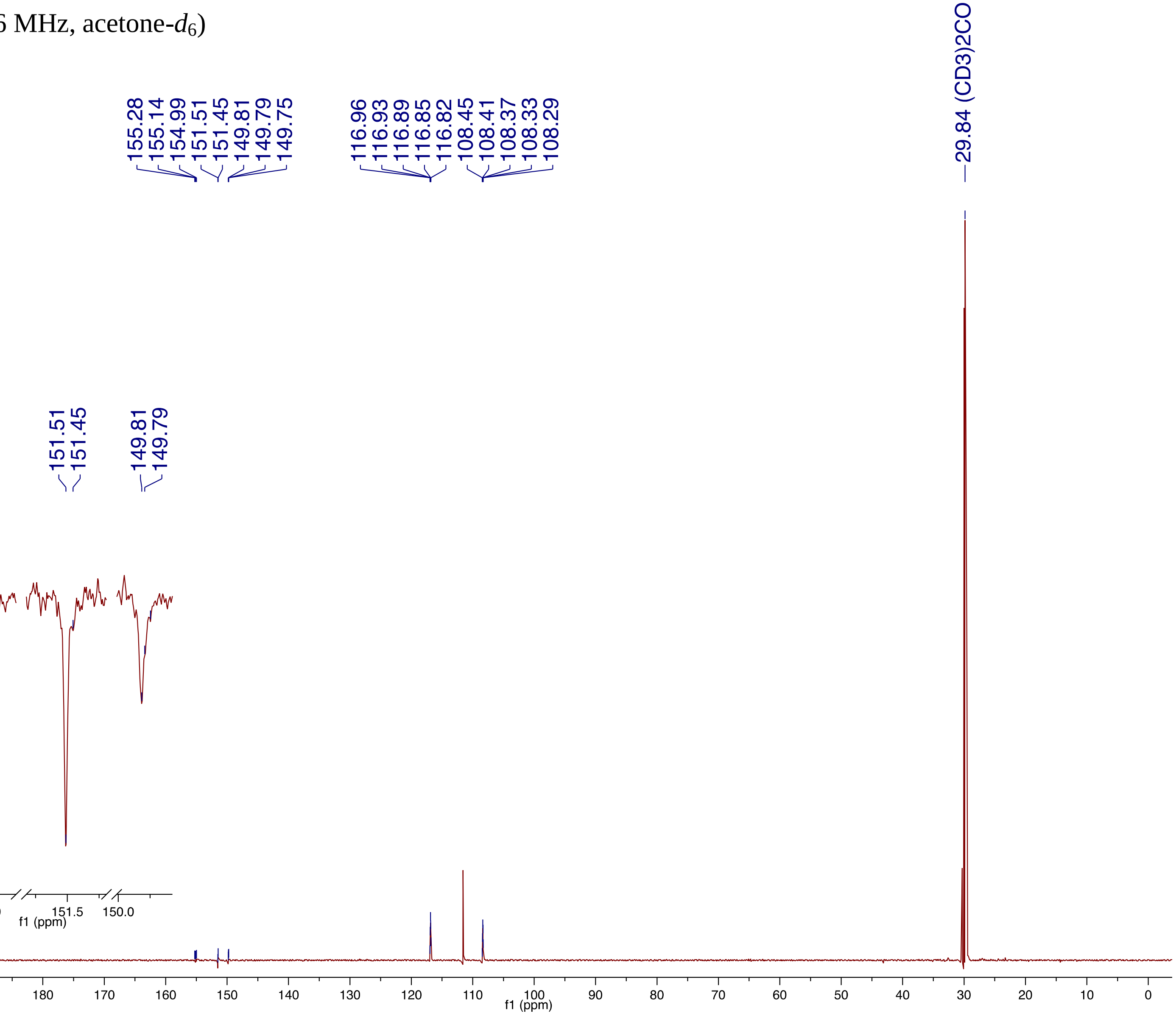

3k: ^{19}F NMR (377 MHz, acetone-d_6)

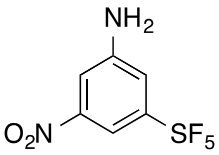

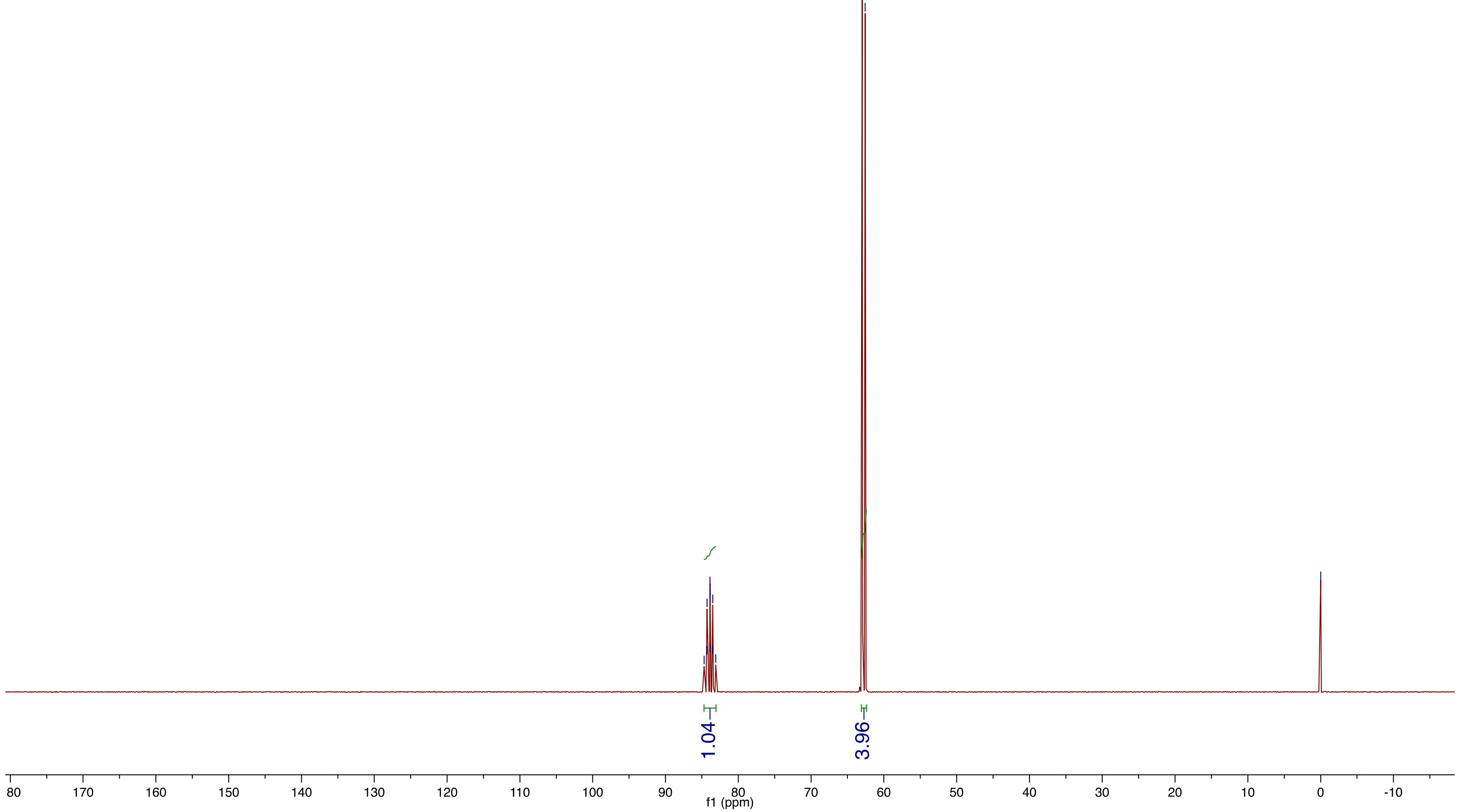

4a: ^{1}H NMR (400 MHz, $CDCl_3$)

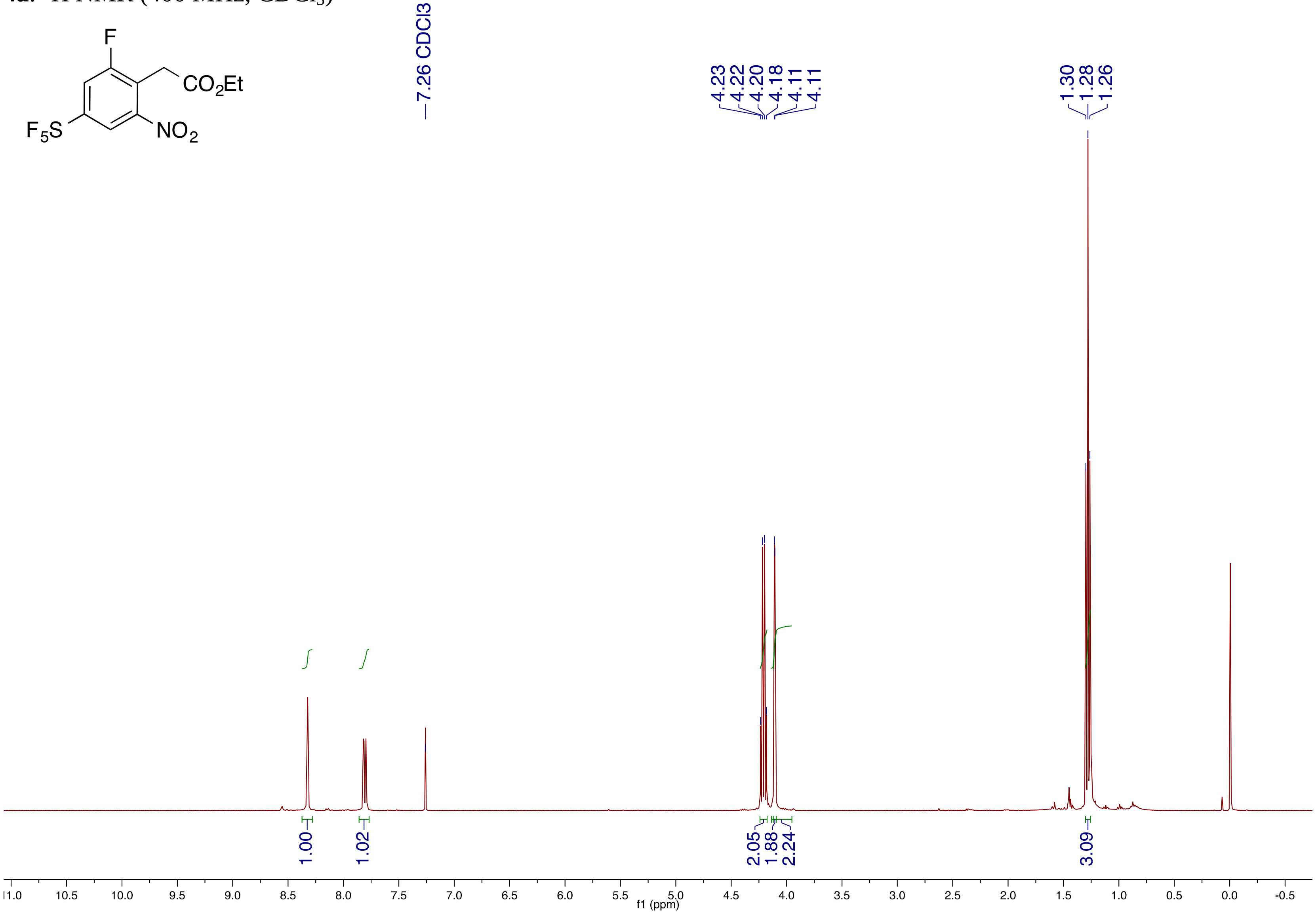

4a: ^{13}C NMR (101 MHz, $CDCl_3$)

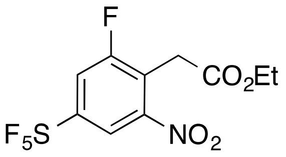

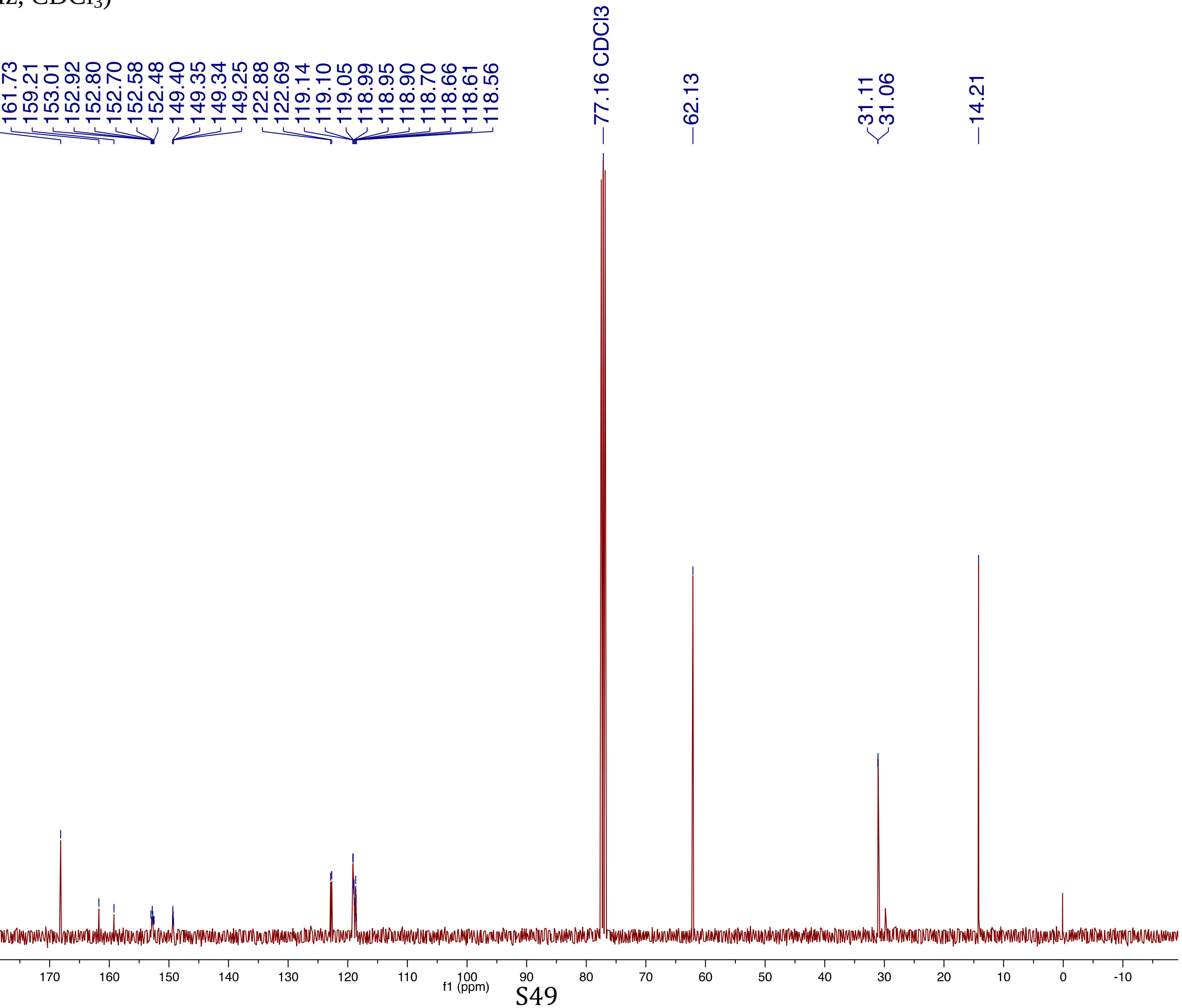

4a: ^{19}F NMR (377 MHz, $CDCl_3$)

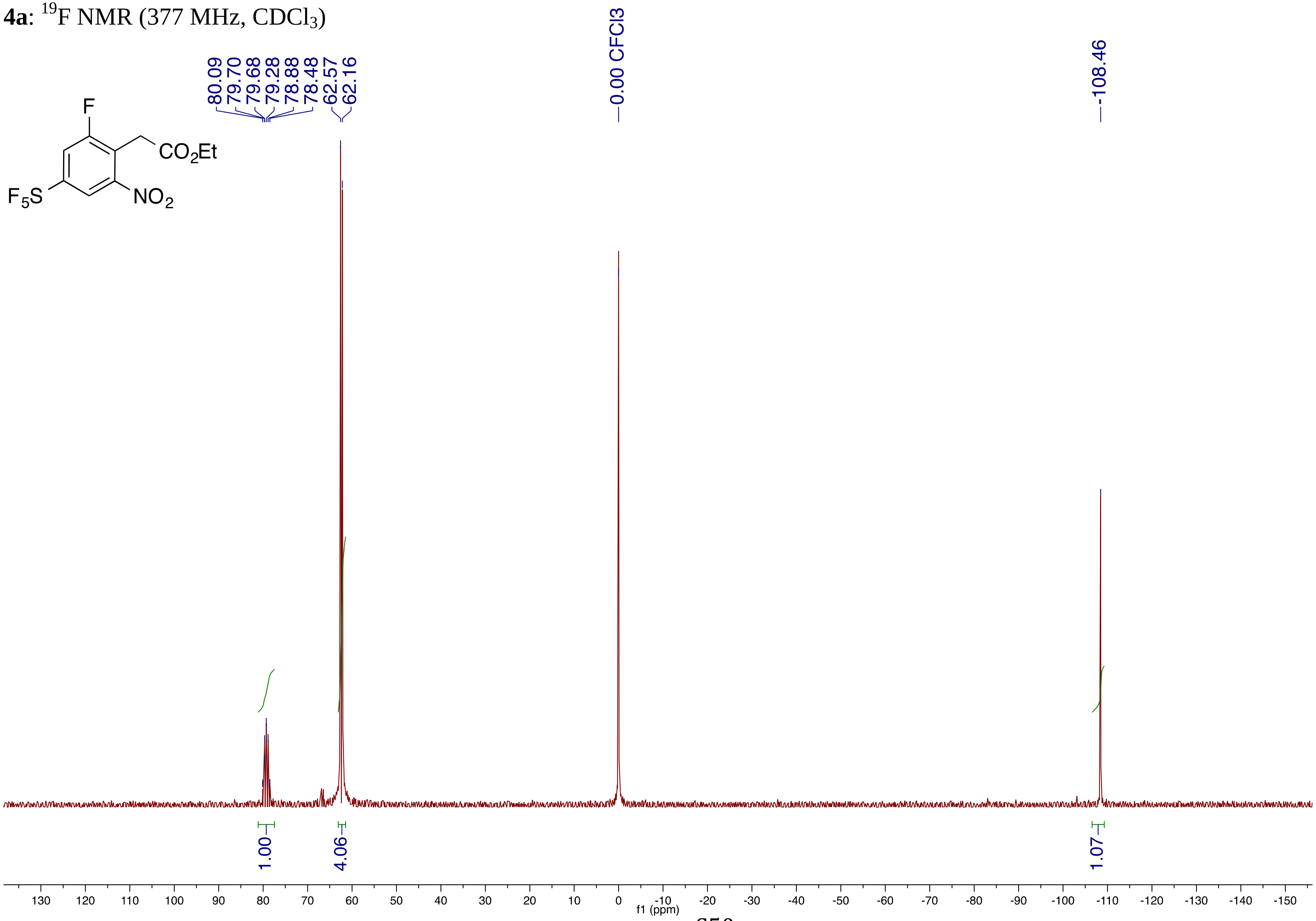

4b: ^{1}H NMR (400 MHz, $CDCl_3$)

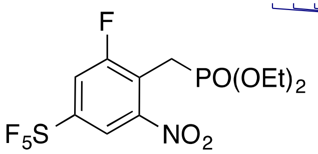

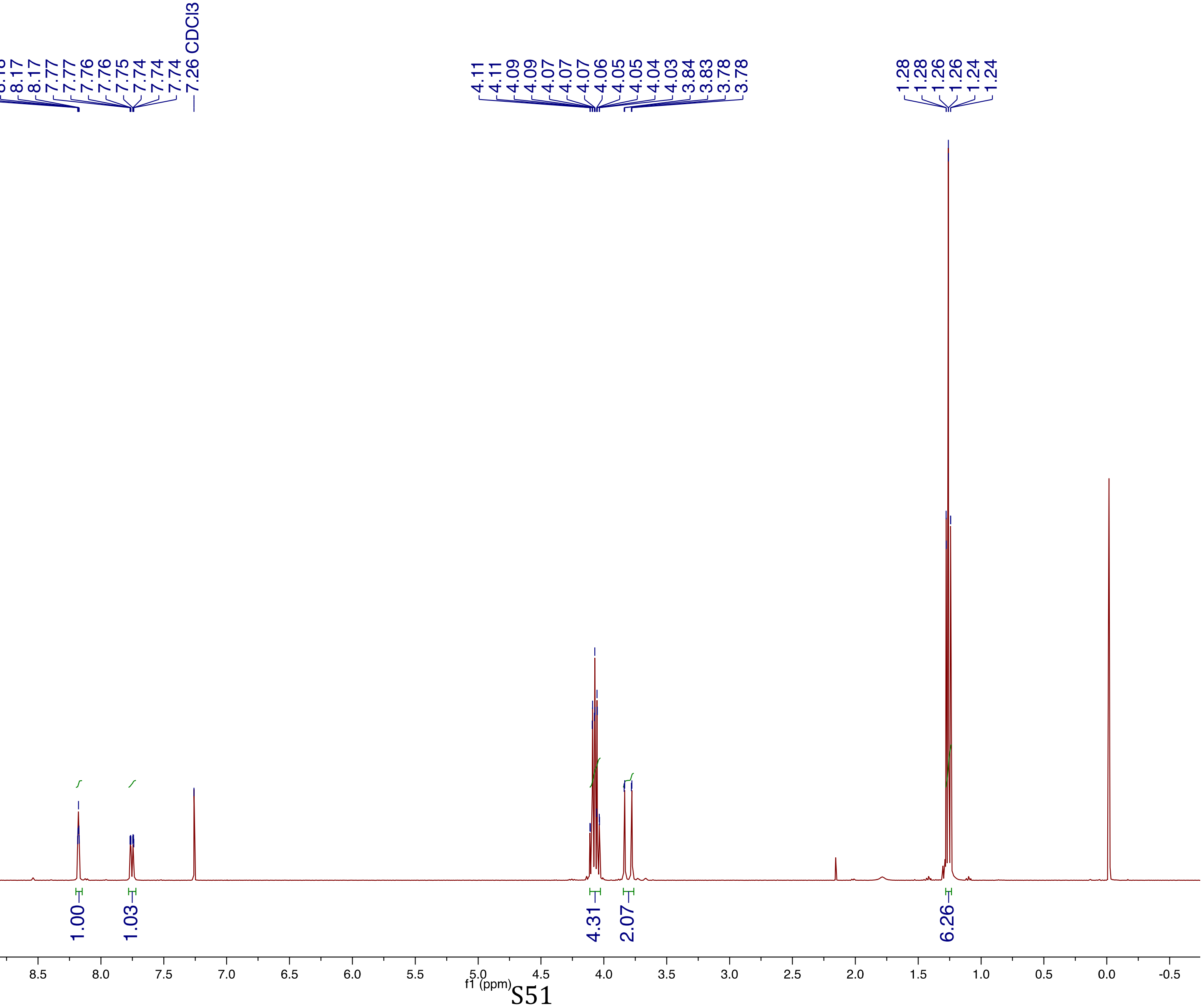

4b: ^{13}C NMR (101 MHz, $CDCl_3$)

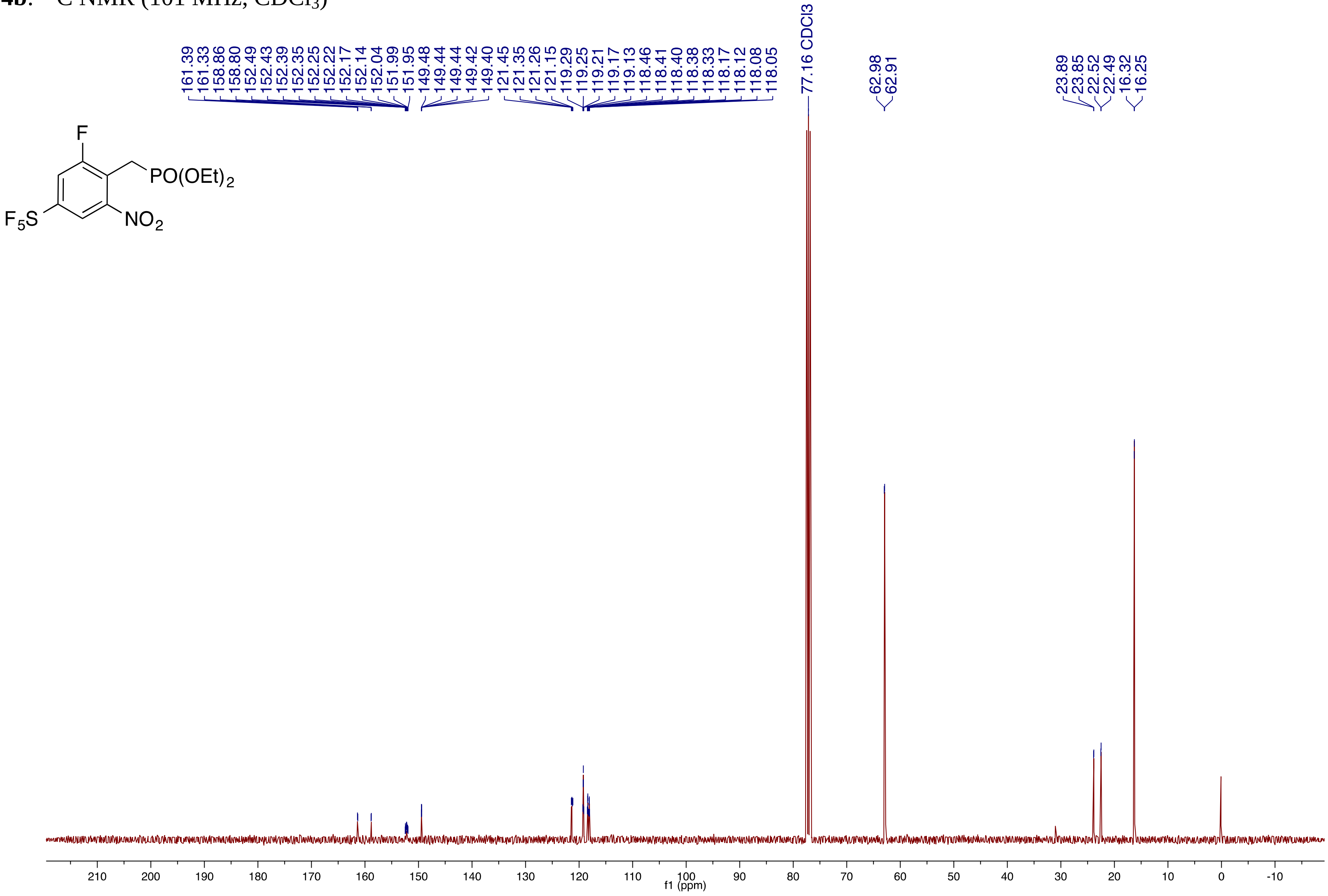

4b: ^{19}F NMR (377 MHz, $CDCl_3$)

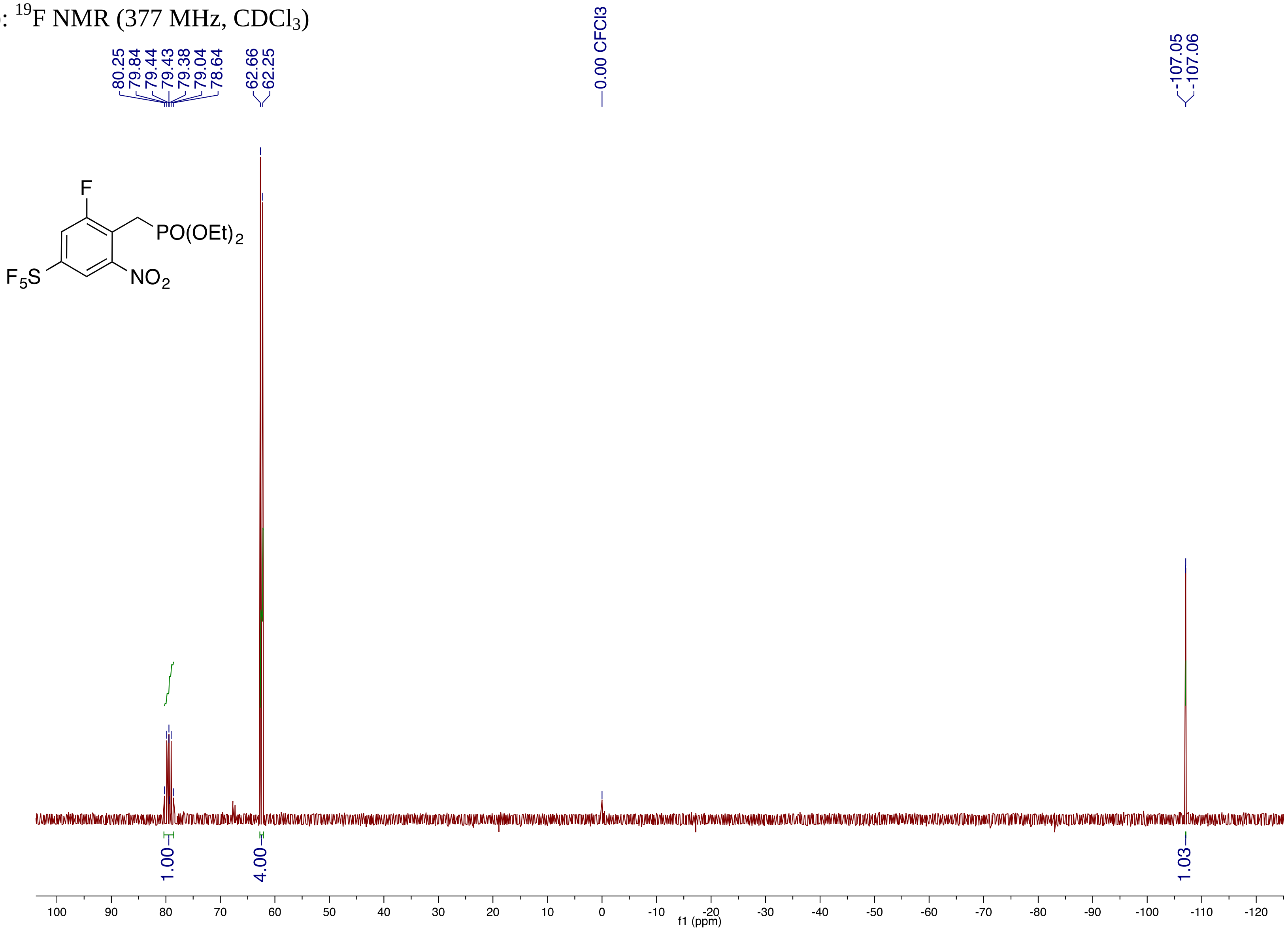

4c: ^{1}H NMR (400 MHz, $CDCl_3$)

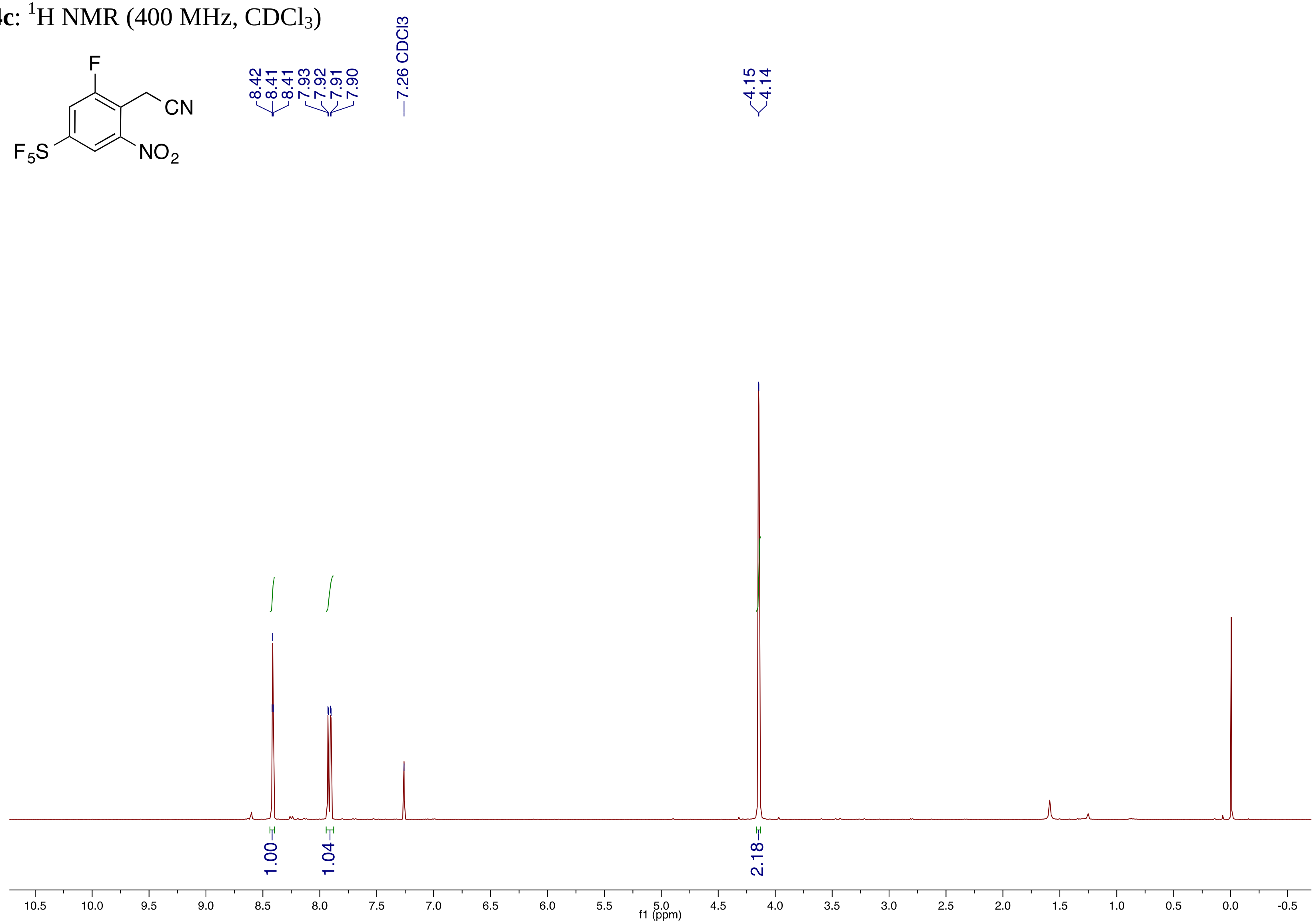

4c: ^{13}C NMR (101 MHz, $CDCl_3$)

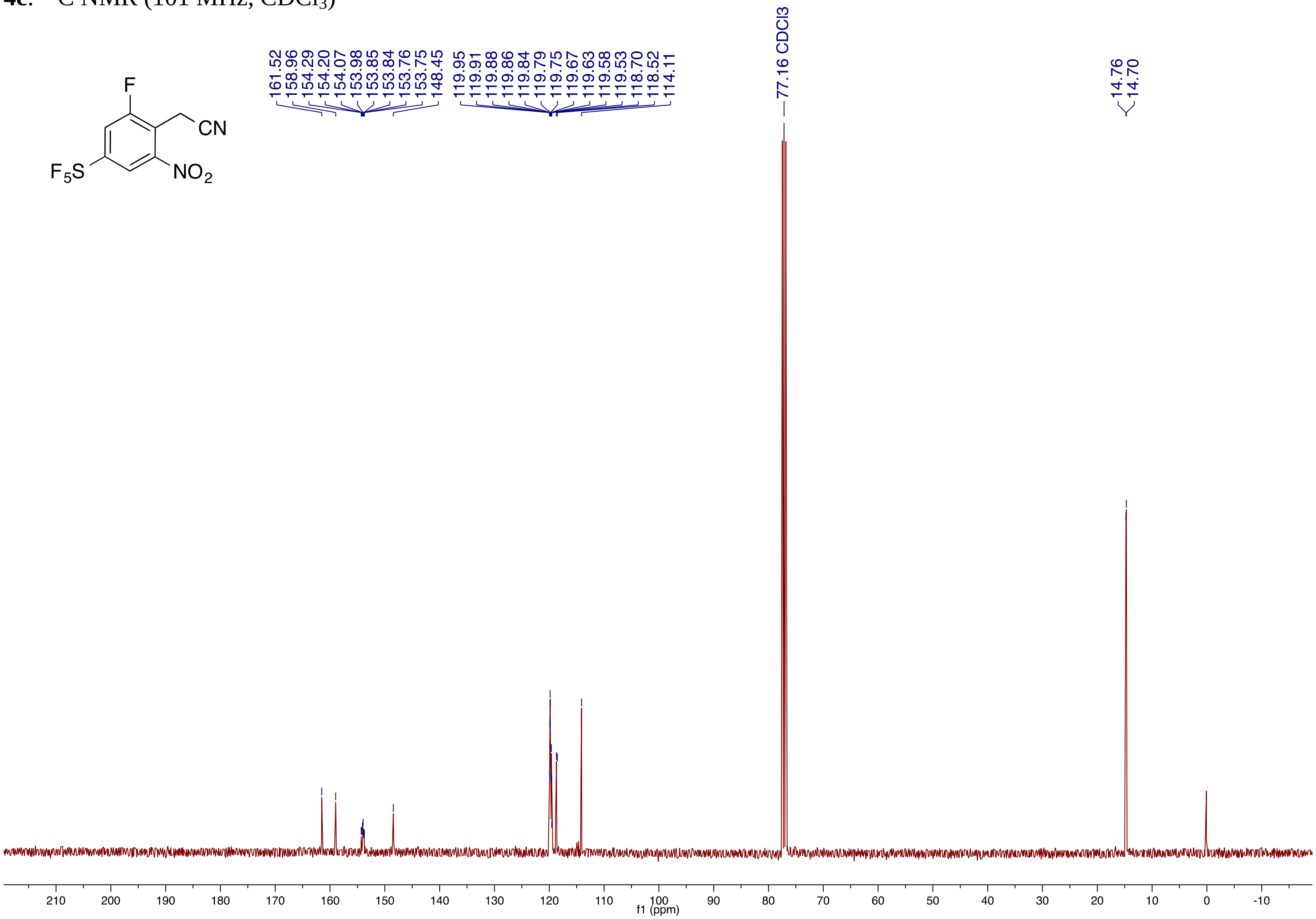

4c: ^{19}F NMR (377 MHz, $CDCl_3$)

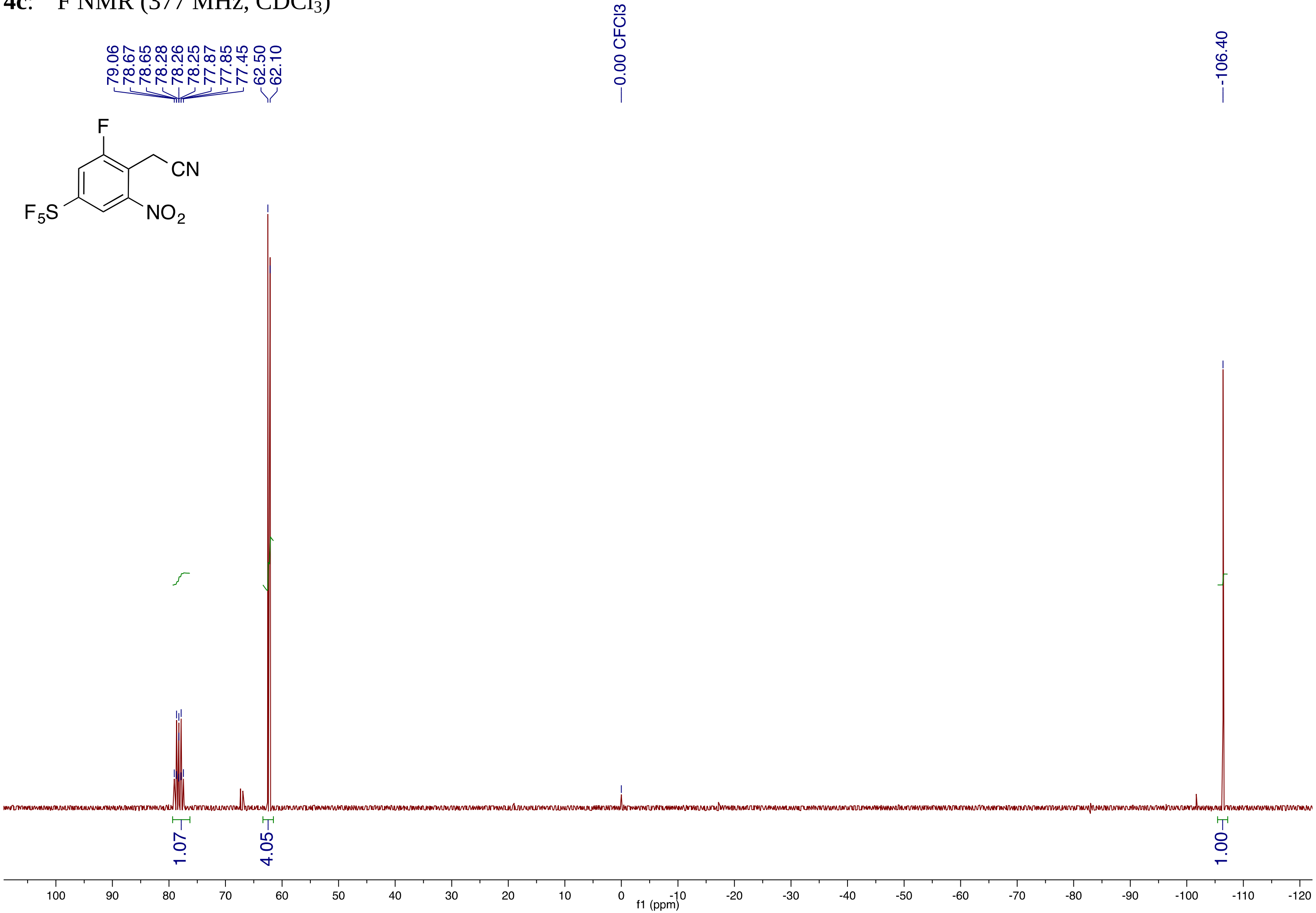

4d: ^{1}H NMR (400 MHz, $CDCl_3$)

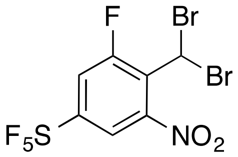

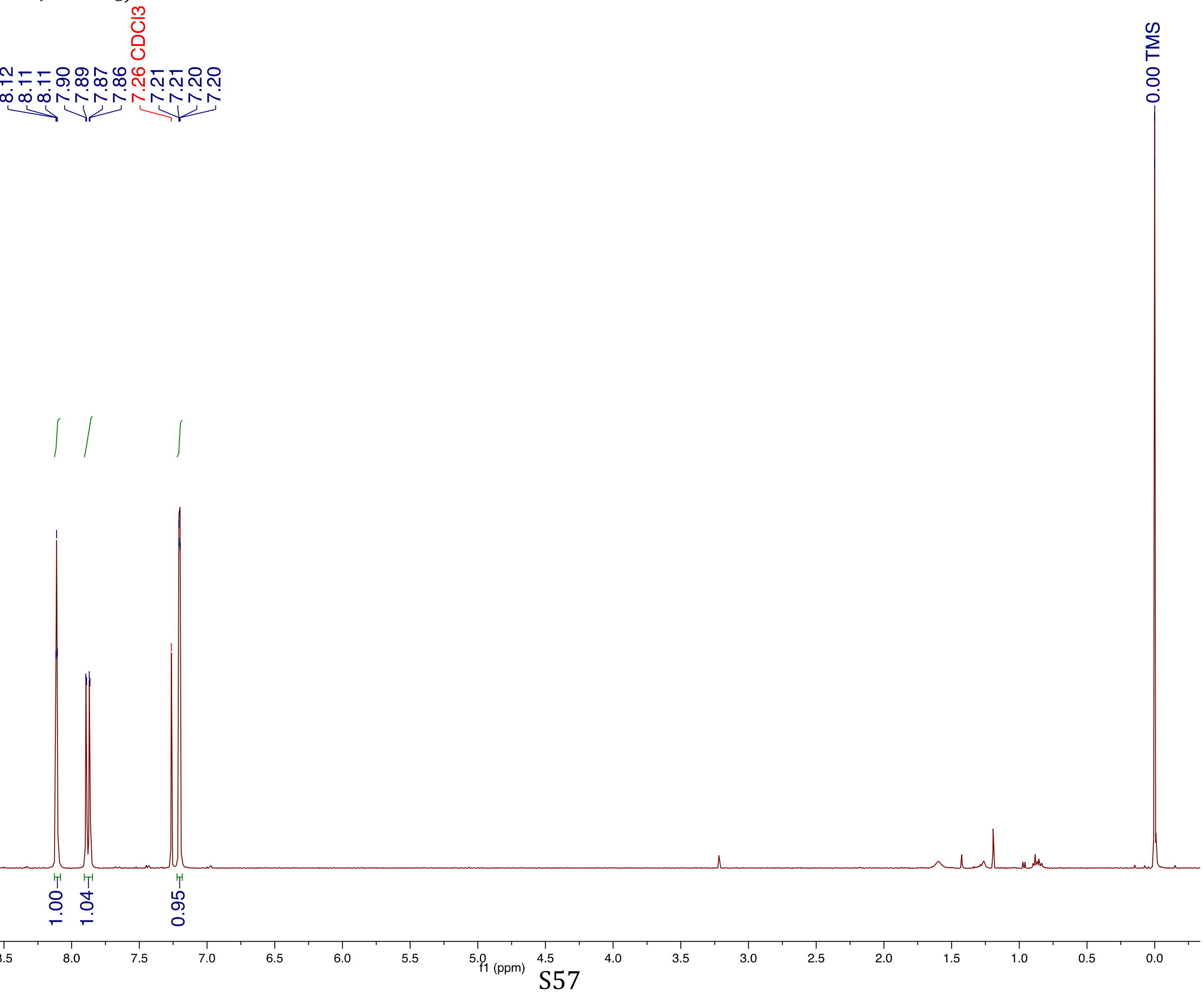

4d: ^{13}C NMR (101 MHz, $CDCl_3$)

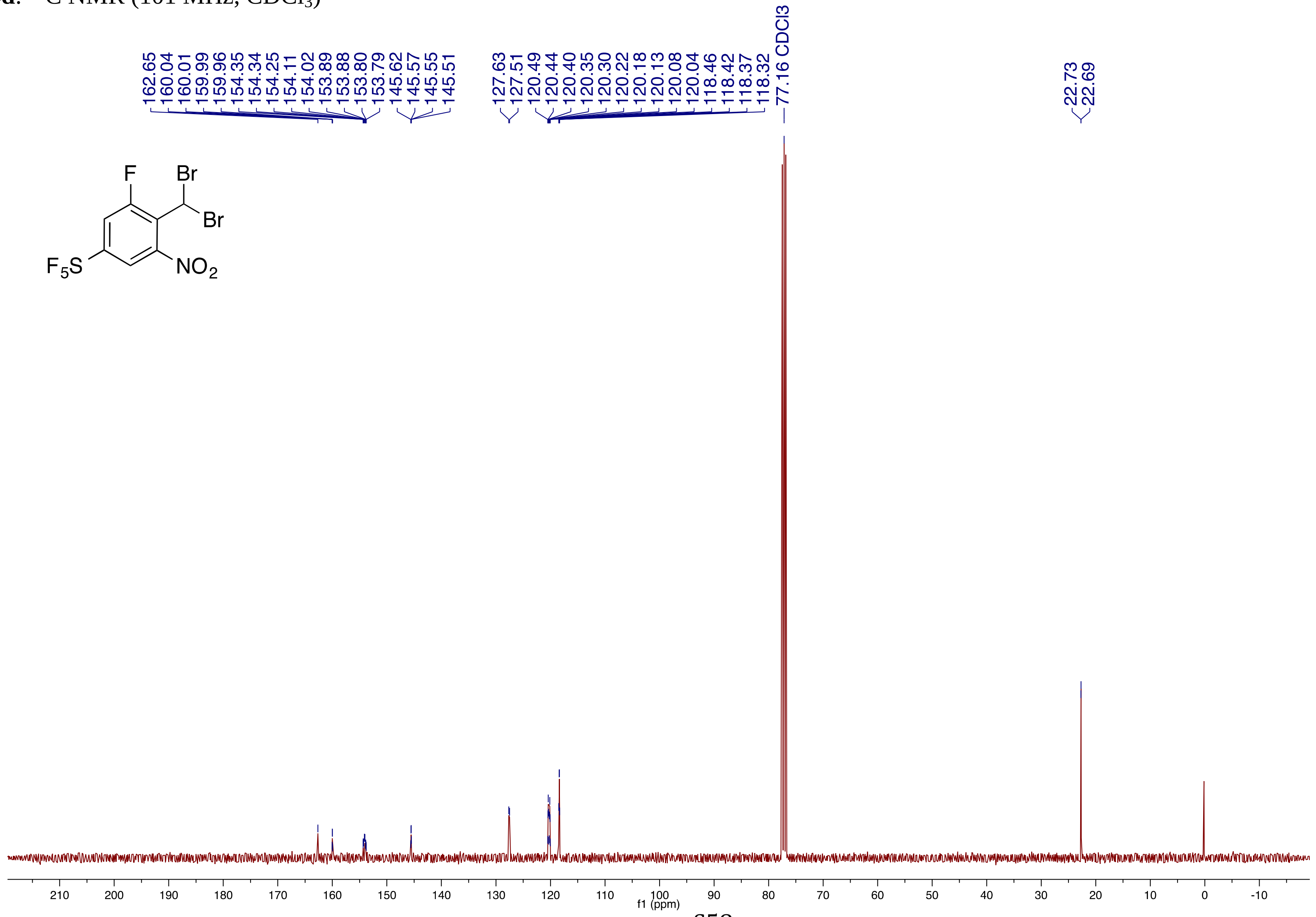

4d: ^{19}F NMR (377 MHz, $CDCl_3$)

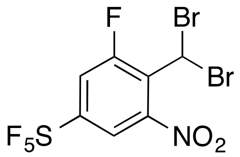

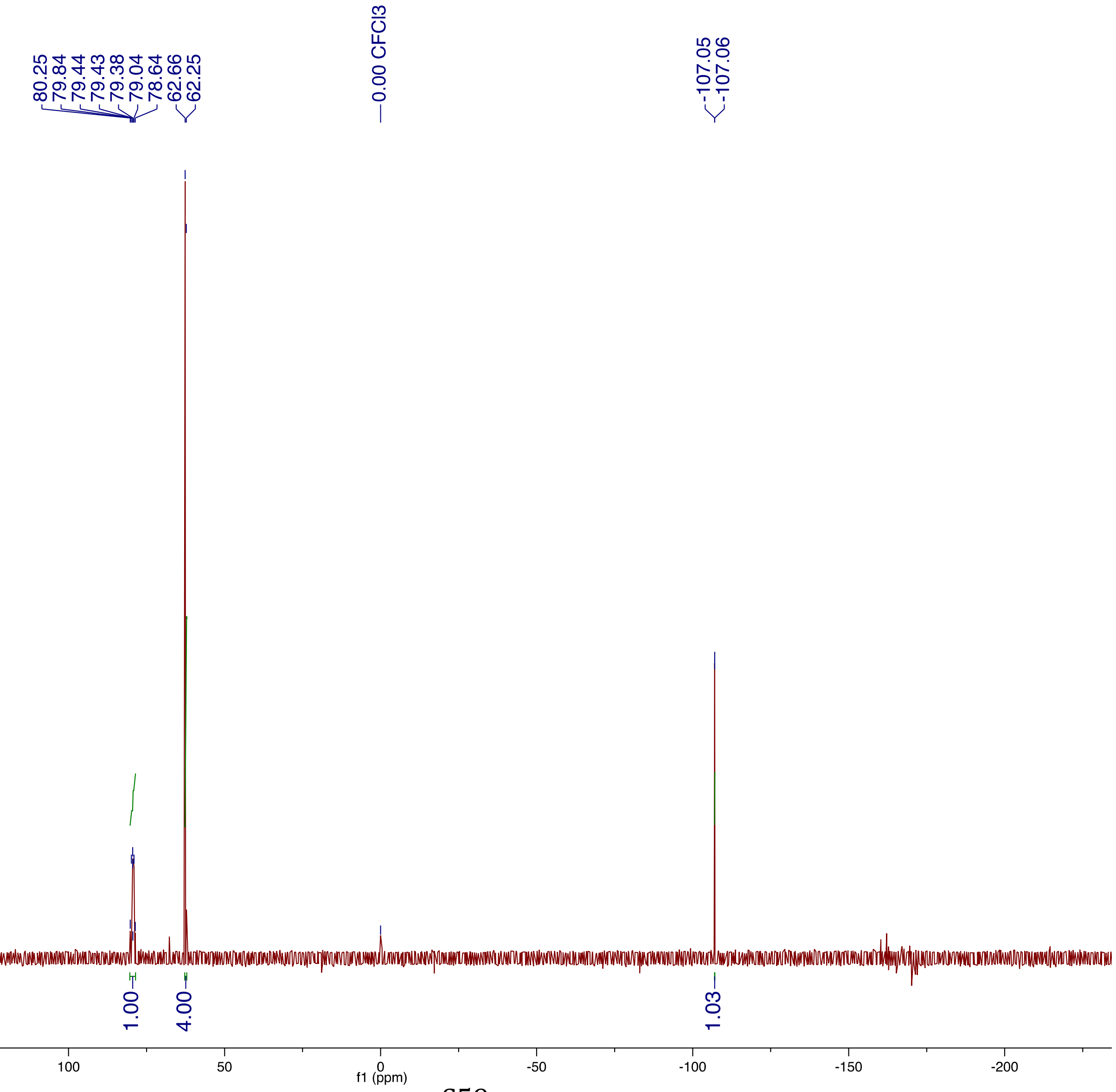

4e: ^{1}H NMR (400 MHz, acetone-d_6)

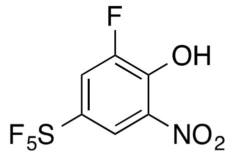

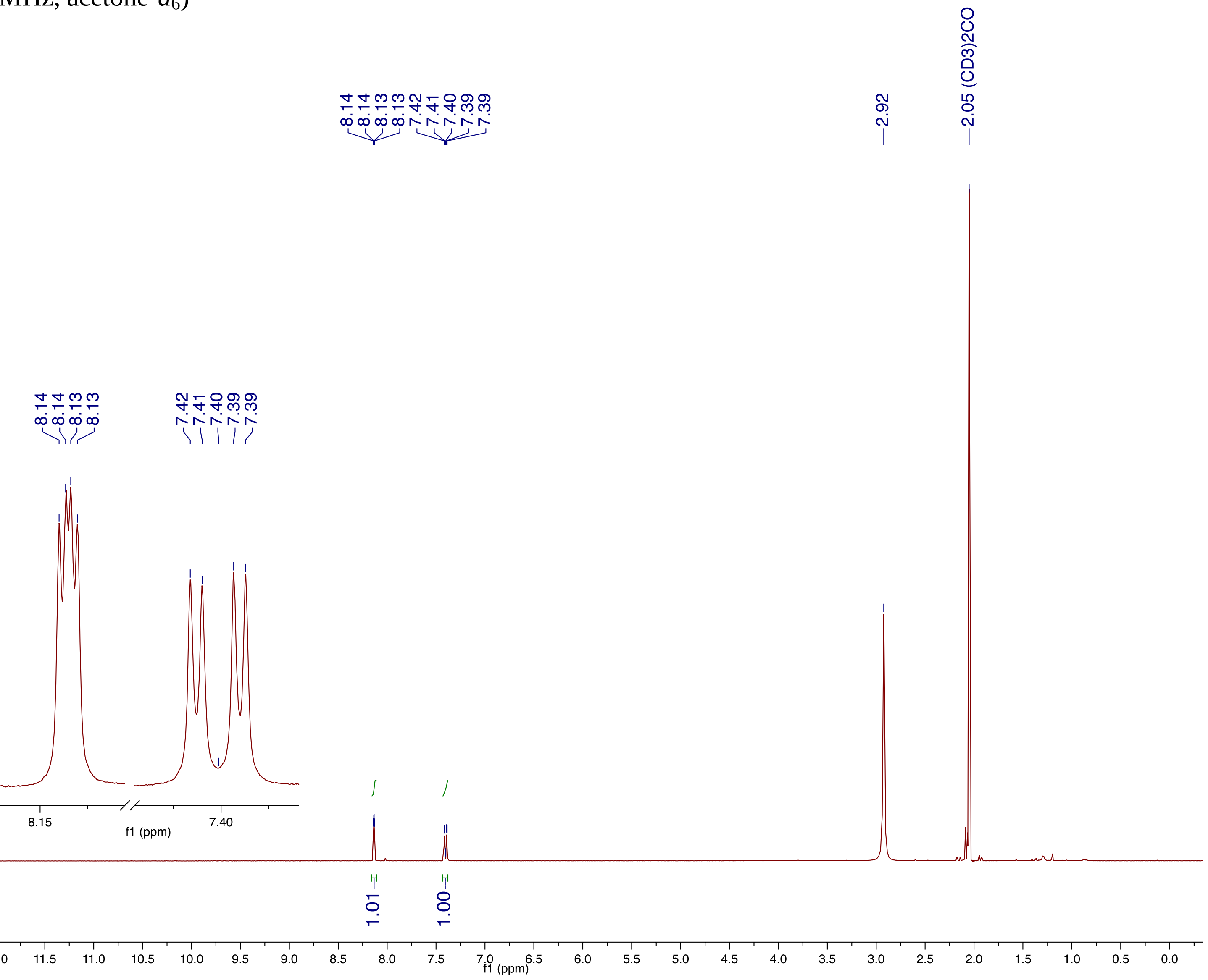

4e: ^{13}C NMR (101 MHz, acetone-d_6)

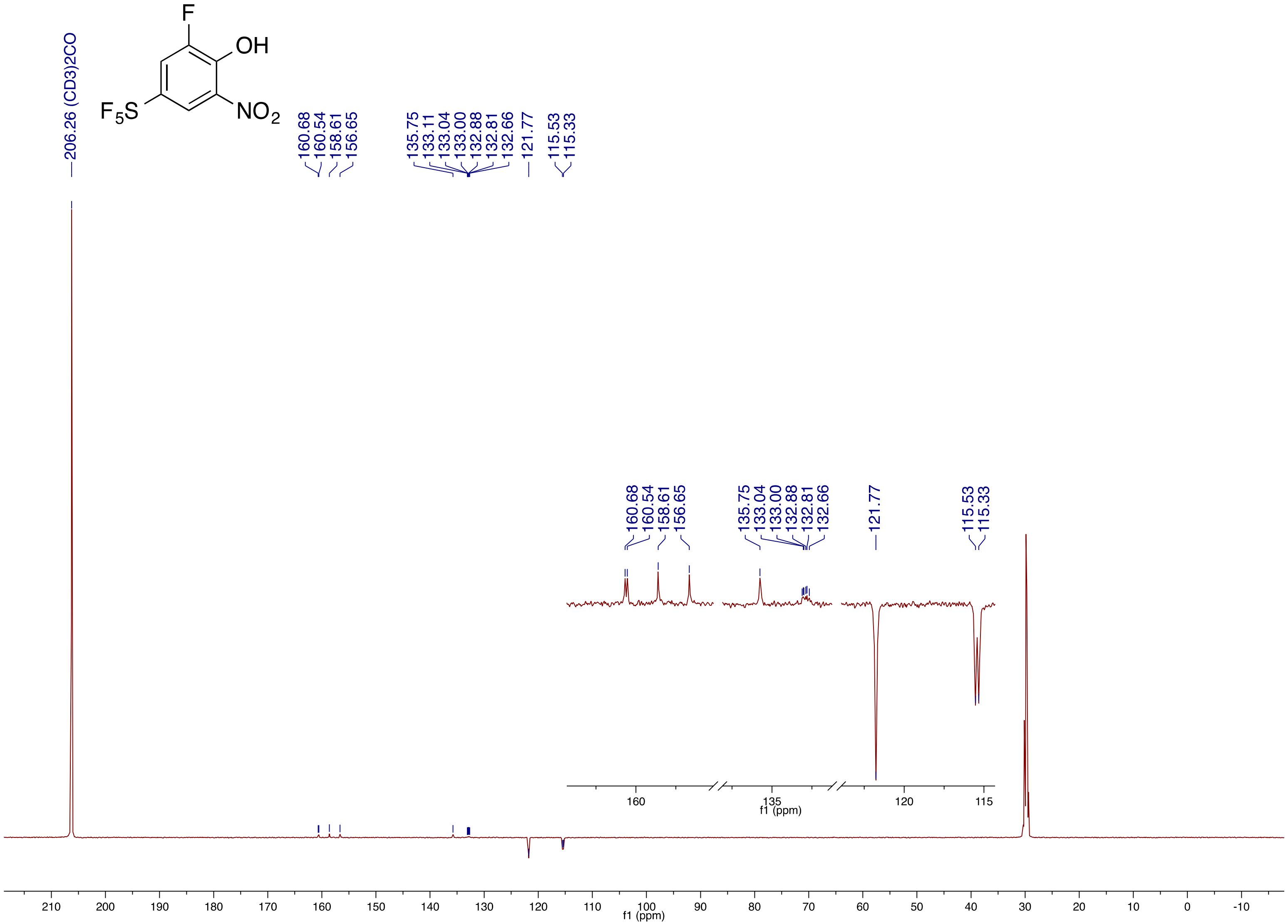

4e: ^{19}F NMR (377 MHz, acetone-d_6)

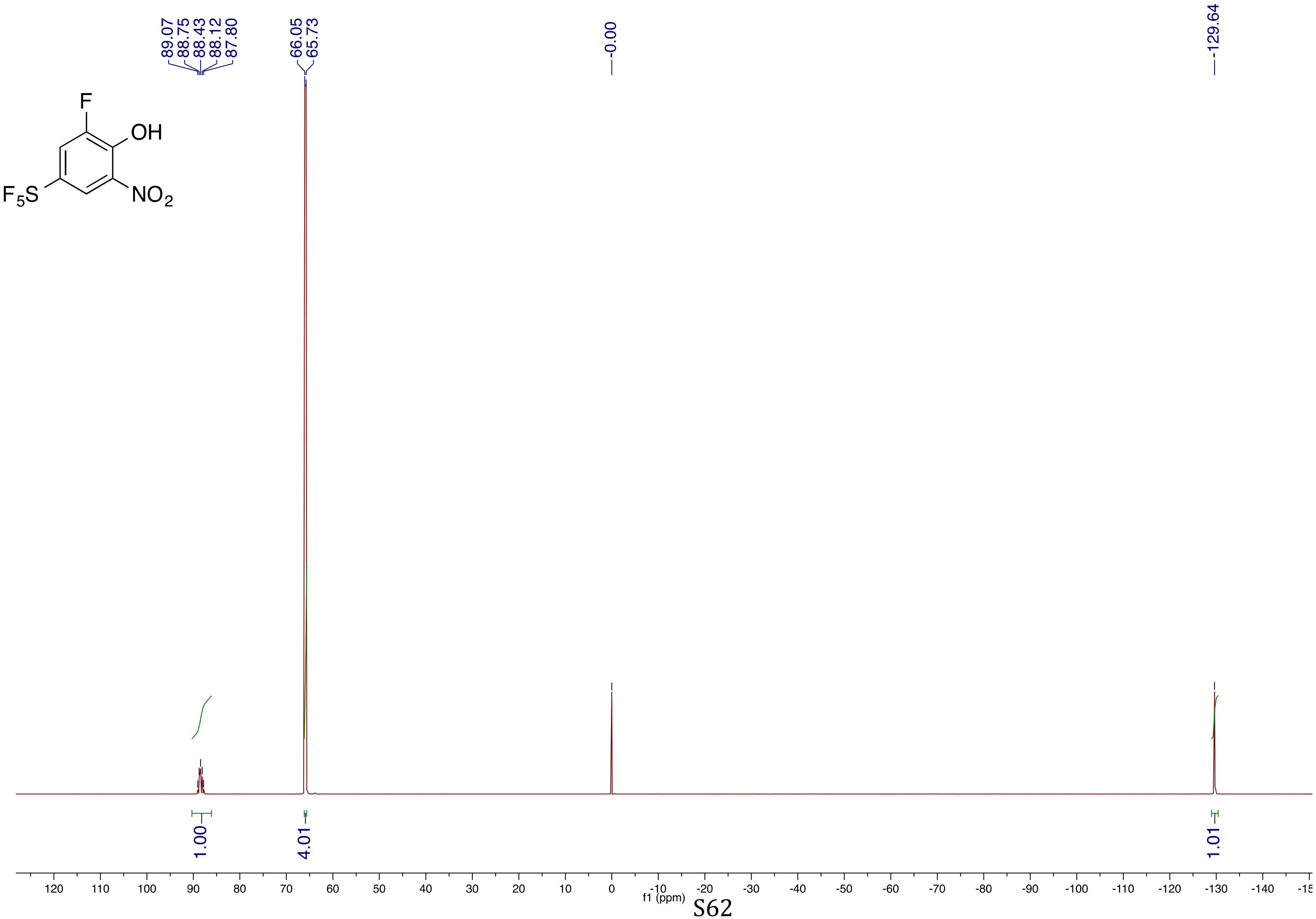

4f: ^{1}H NMR (400 MHz, $CDCl_3$)

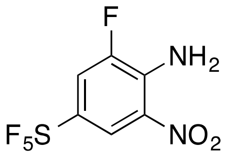

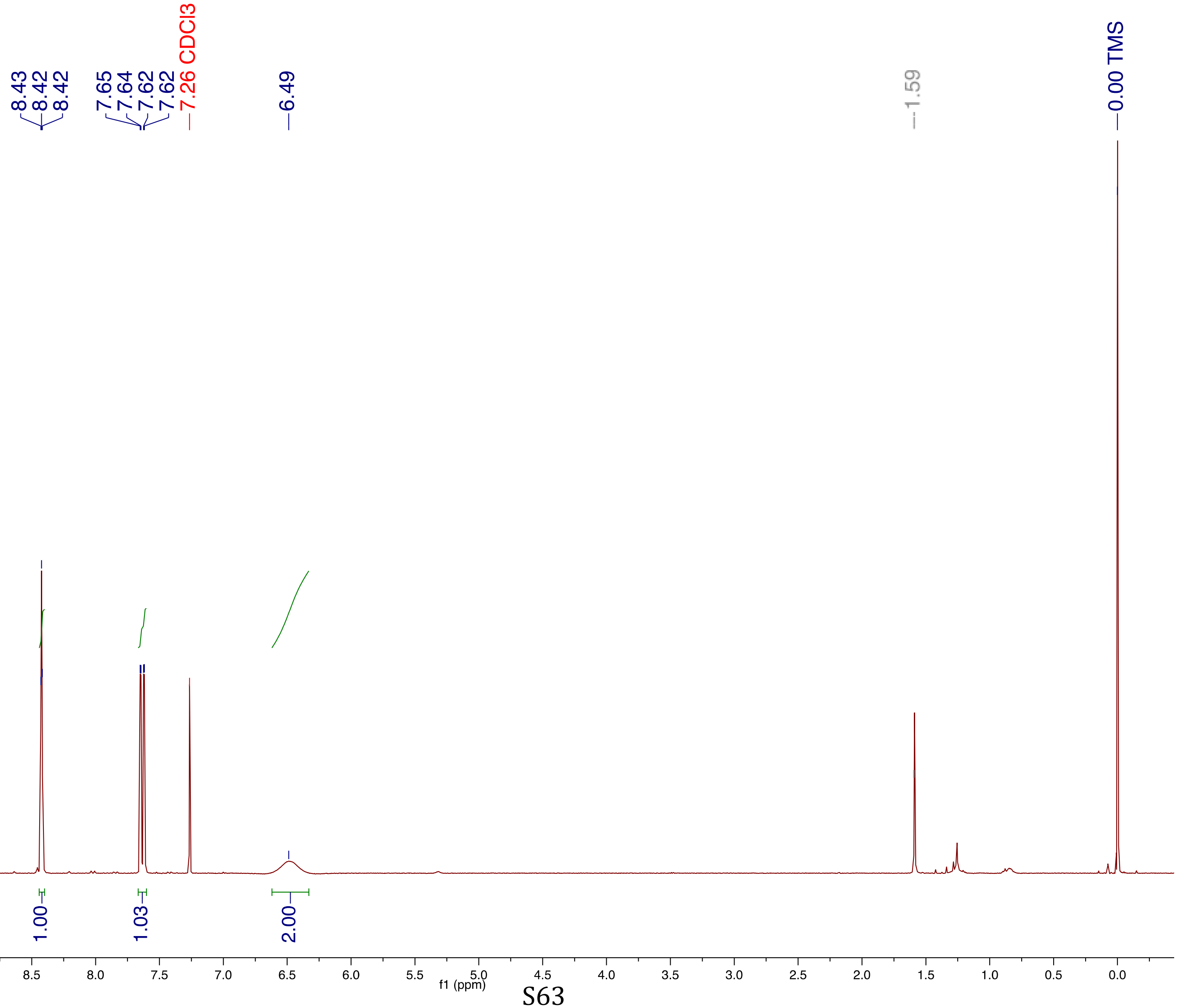

4f: ^{13}C NMR (101 MHz, $CDCl_3$)

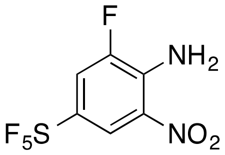

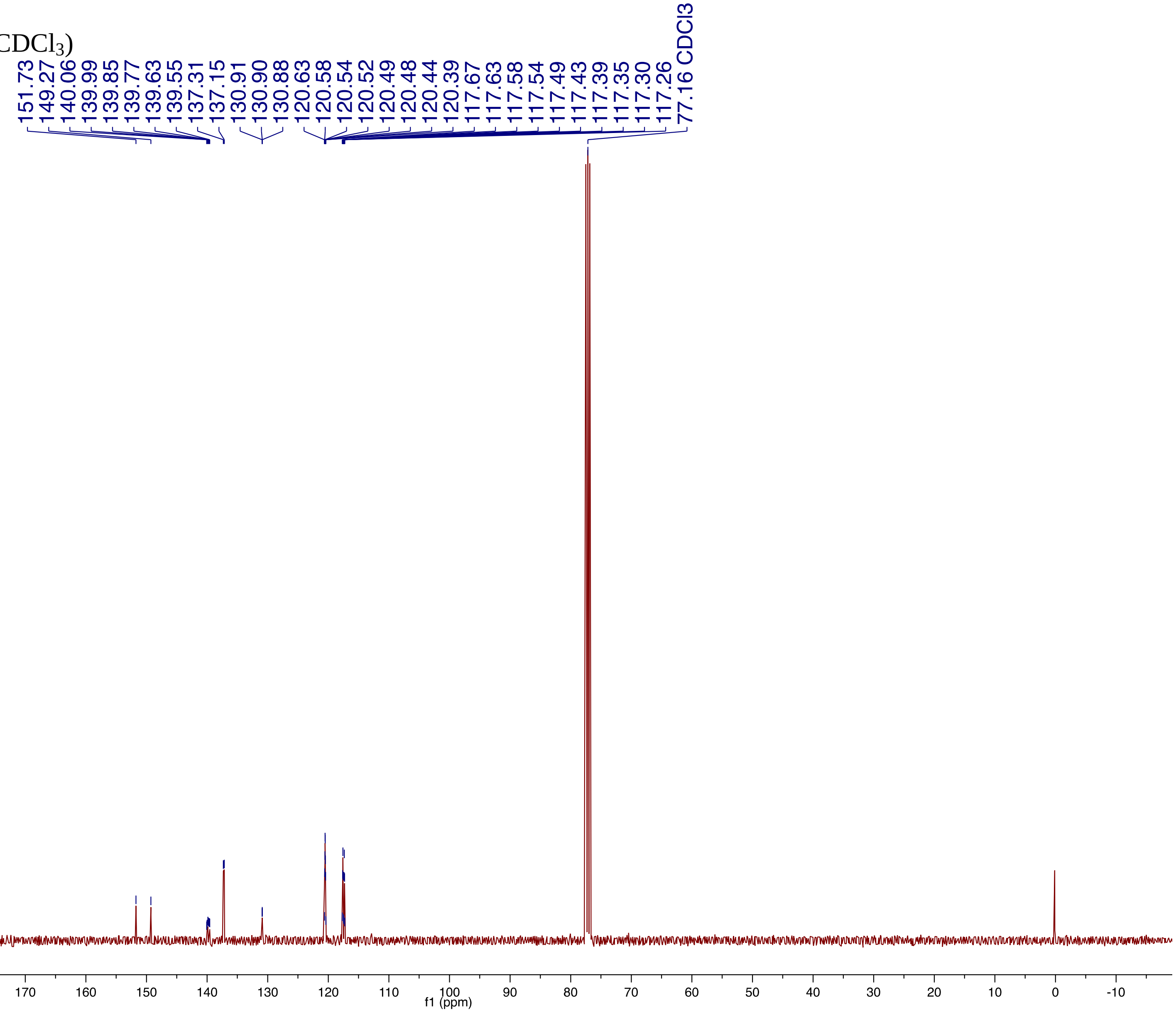

4f: ^{19}F NMR (377 MHz, $CDCl_3$)

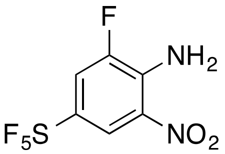

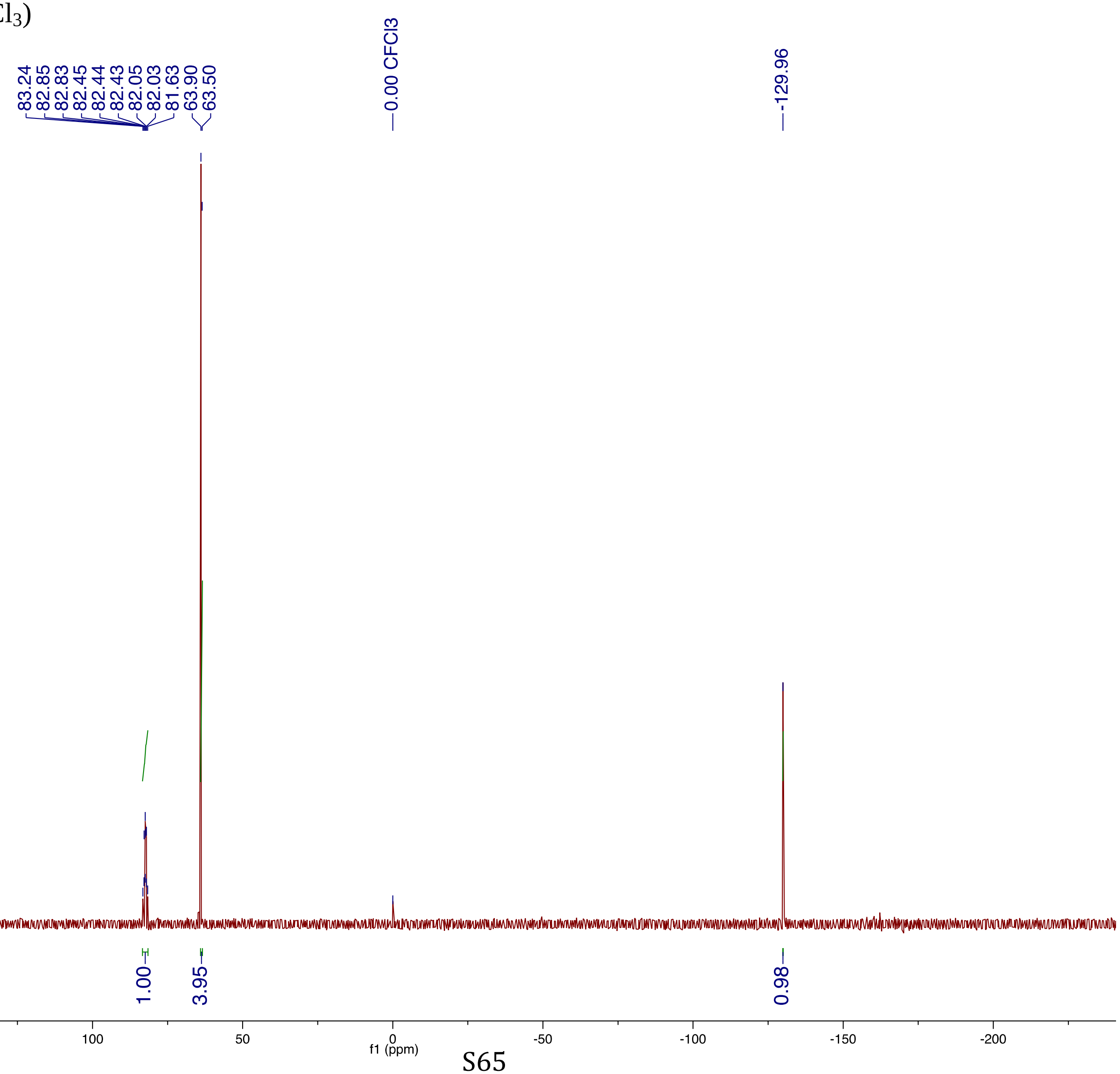

Supplement: File 1 — Experimental part and copies of NMR spectra. [file Beilstein_J_Org_Chem-12-192-s001.pdf]
